# Transcriptomic and Proteomic Analysis of the Tentacles and Mucus of *Anthopleura dowii* Verrill, 1869

**DOI:** 10.3390/md17080436

**Published:** 2019-07-25

**Authors:** Santos Ramírez-Carreto, Rosario Vera-Estrella, Tobías Portillo-Bobadilla, Alexei Licea-Navarro, Johanna Bernaldez-Sarabia, Enrique Rudiño-Piñera, Jerome J. Verleyen, Estefanía Rodríguez, Claudia Rodríguez-Almazán

**Affiliations:** 1 Departamento de Medicina Molecular y Bioprocesos, Instituto de Biotecnología, Universidad Nacional Autónoma de México, Avenida Universidad 2001, Cuernavaca, Morelos 62210, México; 2 Departamento de Biología Molecular de Plantas, Instituto de Biotecnología, Universidad Nacional Autónoma de México, Avenida Universidad 2001, Cuernavaca, Morelos 62210, México; 3 Unidad de Bioinformática, Bioestadística y Biología Computacional. Red de Apoyo a la Investigación, Coordinación de la Investigación Científica, Universidad Nacional Autónoma de México-Instituto Nacional De Ciencias Médicas y Nutrición Salvador Zubirán, Calle Vasco de Quiroga 15, Tlalpan, C.P. 14080, Ciudad de México, México; 4 Departamento de Innovación Biomédica, CICESE, Carretera Ensenada-Tijuana 3918, Ensenada, BC C.P. 22860, México; 5 Unidad Universitaria de Secuenciación Masiva y Bioinformática, Instituto de Biotecnología, Universidad Nacional Autónoma de México, Avenida Universidad 2001, Cuernavaca, Morelos 62210, México; 6 Division of Invertebrate Zoology, American Museum of Natural History, Central Park West at 79th Street, New York, NY 10024, USA

**Keywords:** sea anemone, *Anthopleura dowii*, venom, mucus, tentacle, transcriptome, proteome

## Abstract

Sea anemone venom contains a complex and diverse arsenal of peptides and proteins of pharmacological and biotechnological interest, however, only venom from a few species has been explored from a global perspective to date. In the present study, we identified the polypeptides present in the venom of the sea anemone *Anthopleura dowii* Verrill, 1869 through a transcriptomic and proteomic analysis of the tentacles and the proteomic profile of the secreted mucus. In our transcriptomic results, we identified 261 polypeptides related to or predicted to be secreted in the venom, including proteases, neurotoxins that could act as either potassium (K^+^) or sodium (Na^+^) channels inhibitors, protease inhibitors, phospholipases A2, and other polypeptides. Our proteomic data allowed the identification of 156 polypeptides—48 exclusively identified in the mucus, 20 in the tentacles, and 88 in both protein samples. Only 23 polypeptides identified by tandem mass spectrometry (MS/MS) were related to the venom and 21 exclusively identified in the mucus, most corresponding to neurotoxins and hydrolases. Our data contribute to the knowledge of evolutionary and venomic analyses of cnidarians, particularly of sea anemones.

## 1. Introduction

Sea anemones are among the oldest animals with the ability to produce venom, which is used in defense, depredation, and intra-specific competition [1,2]. Unlike other venomous animals, sea anemones concentrate their venom in structures called cnidae, distributed across the different regions of the polyp (tentacles, acrorhagi, actinopharynx, column, mesenterial filaments), but with major abundances in the tentacles [3,4,5]. Among the polypeptides there are neurotoxins that block either sodium (Na^+^) or potassium (K^+^) channels, type A2 phospholipases, metalloproteases, pore-forming proteins, and protease inhibitors [1,4,6,7]. Neurotoxins can modify or block the Na^+^ and K^+^ channels, respectively, resulting in the immobilization of prey, which could be useful for the study and treatment of nervous system channelopathies [8,9]. The most frequently studied sea anemone toxin is the ShK toxin from *Stichodactyla helianthus* [10]. This peptide has the ability to block the Kv1.3 channels of T lymphocytes, inhibiting their activation and therefore acting as an immunosuppressant [11,12]. Other toxins have shown activity on pain-related channels, such as the first sea anemone toxin specific for the HERG channel (APETx1), toxin from sea anemone *Anthopleura elegantissima*, which inhibits the acid-sensing ionic channel 3 (ASIC3), an acid pain sensor, and participates in inflammatory pathways [13,14]. The analgesic polypeptides 1-3 (APHC1-3) from sea anemone *Heteractis crispa* inhibit the pain vanilloid receptor (TRPV1), which is involved in conditions such as peripheral neuropathic pain, epilepsy and cancer pain [15,16,17].

Phospholipases (PLA) participate in diverse processes such as cell membrane metabolism, dietary lipid catabolism, and inflammatory processes [6]. PLA2 in venom has been associated with a variety of pharmacological activities with neurotoxic, hypotensive, proinflammatory, platelet aggregation, hemolytic, bactericidal, and myotoxic effects [18]. Protease inhibitors are a group of peptides and proteins that have potential medical applications in cardiovascular, neurodegenerative, inflammatory, and even immunological diseases, based on the control of proteolysis [19,20]. Several peptides with protease inhibitory activity have been isolated from the mucus and extracts of the tentacles, acrorhagi, and the total body of sea anemones [21].

The omics techniques, both proteomic and transcriptomic, have enabled further exploration of the components of sea anemone venom from a few species [22,23,24,25,26,27], as well as in another venomous animal species (scorpion, spider, conus snail, snake) [28,29,30,31]. Transcriptomic methods have been used to investigate the differential expression of components of venom among tissues or certain stages of development in a particular species of sea anemone [22,32,33,34]. In addition, they can be used to identify components poorly represented in the venom, which cannot be identified by proteomic and other methods requiring large sample concentrations. The amount of material necessary for these studies is minimal and sometimes does not require the organism to be sacrificed. Proteomic analyses provide information on the identification and quantification of the repertoire of overall proteins of different anatomical regions or even secretions (mucus) of a sea anemone. The proteomic profiles of secretions and cnidae isolated from sea anemones have increased the knowledge of the protein composition and toxins present in their venom [23,35]. Moreover, these kinds of analyses have identified the most representative components in mucus under stress conditions [24,27,36]. Transcriptomics and proteomics are both methods with limitations (e.g., transcriptions expression does not identify regulatory processes or post-transcriptional modifications) [37]. Proteomic techniques do not have the sensitivity to detect low abundant proteins [38]. However, transcriptomic–proteomic integrative analyses enrich the biological information of organisms. Only two studies of sea anemones have analyzed the transcriptome and proteome simultaneously [23,39]; this approach resulted in the discovery of sequences potentially representing new unidentified families of toxins in *Stichodactyla haddoni*, a species previously studied for a long time [23]. By using tandem mass spectrometry (MS/MS), it is possible to identify part of a protein by de novo sequencing, but if the protein has not been previously described, it is not possible to identify it correctly. Identification of the whole protein can be performed only if the transcriptome is available. The combined use of transcriptome and proteome is a powerful set of techniques for protein de novo identification.

In this work, we explored the venom of the sea anemone *Anthopleura dowii* Verrill, 1869 through transcriptomic and proteomic analyses of the tentacle and the mucus proteome. Although partial characterization of the venom from sea anemones has been reported, we are the first to report the transcriptome–proteome profiles of *A. dowii*. This species belongs to one of the most studied genera of sea anemones and lives in shallow waters along the Pacific coast from México to Panama. Only one study about the components of its venom has been completed using a total body extract [40]. The transcriptomic analysis and characterization of some toxins (pore forming toxins, Na^+^ and K^+^ voltage-gated channel toxins, protease inhibitors) of the genus *Anthopleura* have been reported [41,42,43]. Our results show a set of transcripts and proteomic data that reveal the presence of previously unreported diversity of polypeptides present in *A. dowii*, such as neurotoxins that could act on Na^+^ or K^+^ channels, protease inhibitors, cytotoxic components, phospholipases A2, proteases, the CAP superfamily (Cysteine-rich secretory proteins, Antigen 5, and Pathogenesis-related 1 proteins), lectins, and several hydrolases. Our results show the complexity of the polypeptides present in the venom of *A. dowii* and the versatility of the uses that these compounds could have in the pharmaceutical industry.

## 2. Results and Discussion

### 2.1. Venom Components Identified in the Transcriptome of the Tentacles of A. dowii

**Figure 1:**
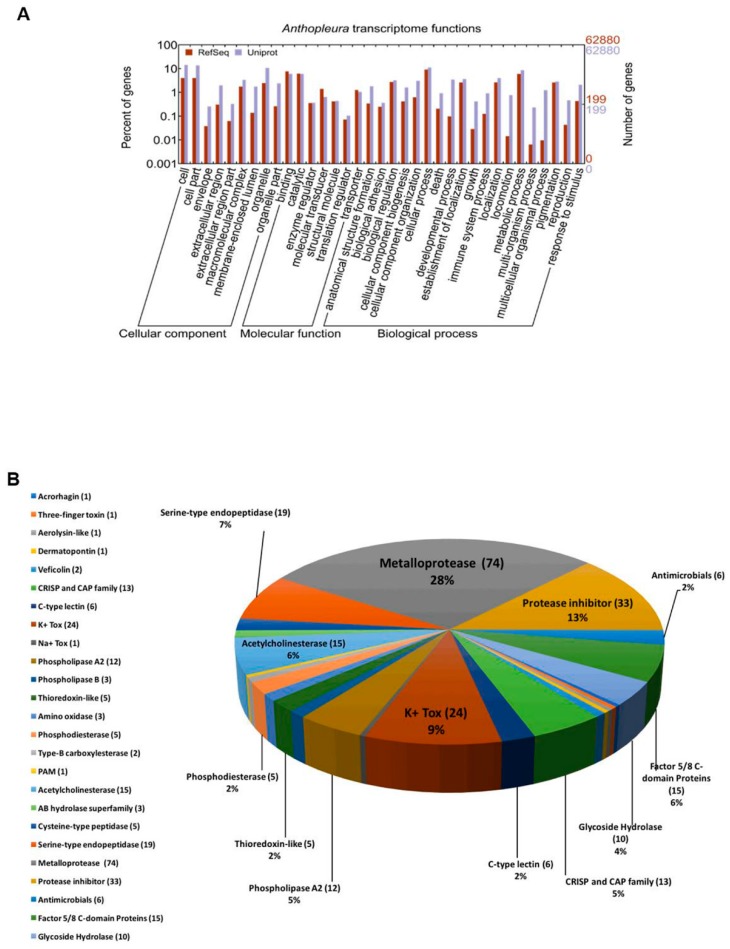
The transcriptome of *Anthopleura dowii* Verrill, 1869: (**A**) proportion and number of contigs assigned to the Gene Ontology (GO) term categories; (**B**) putative toxins identified in the transcriptome by the Protein Basic Local Alignment Search Tool (BLASTP) search against UniProtKB. The chart represents the relative abundance of different transcripts identified as venom components. The number of homologues identified for each putative toxin is shown in parentheses after the family name.

The sequencing and assembly of the tentacle transcriptome of the sea anemone *A. dowii* was generated and subsequently deposited at the National Center for Biotechnology Information (NCBI) database (BioProject: PRJNA329297, and SRA accession: SRP078992) [44]. We made the de novo annotation of 62,880 sequence transcripts, with lengths between 227 and 15,115 bp. A total of 35,832 transcripts were classified in three categories, corresponding to the Gene Onthology (GO) database using the program Blast2GO and the UniProt and RefSeq databases (Figure 1A): biological processes (37.1%), cellular components (19.4%), and molecular function (51.6%). Our results show that most of the transcripts were associated with biological processes (metabolic and cellular). Regarding molecular function, the transcripts were mainly related to catalytic and binding activities, while in terms of cellular components, most were classified as components from part of the cell or organelles, indicating that a greater number of transcripts are related to intracellular functions (Figure 1A). In order to explore the components of the venom from the transcriptome data and to generate a database to complement our proteomic mucus and tentacle data, we selected transcripts with higher identity with protein databases using a protein query (BLASTP) that were linked to components of the venom in our automatically generated annotation, and analyzed their corresponding amino acid sequences in detail one by one.

A total of 261 transcripts were identified and classified into 25 groups according to probable functions, which were estimated based on their sequence homology at the amino acid level with peptides and proteins from the UniprotKB databases (Figure 1B, Supplementary Table S1). Most of the components corresponded to metalloproteases (28%), inhibitors of proteases (13%), toxins acting on ion channels regulated by K^+^ (9%), proteases of serine (7%), prothrombin activators (Factor 5/8 C domain proteins) and acetylcholinesterases (at 6% each), type A2 PLA and members of the CAP superfamily (5% each), and glycosidases (4%) (Figure 1B). In general, the main components identified in the transcriptome corresponded to proteolytic enzymes. A similar trend has also been observed in the transcriptomes of other sea anemones [23,25], other cnidarians and even in other phyla of venomous animals [45,46,47,48].

### 2.2. Venom Components Identified in the Proteome of the Tentacle and Mucus of A. dowii

**Figure 2:**
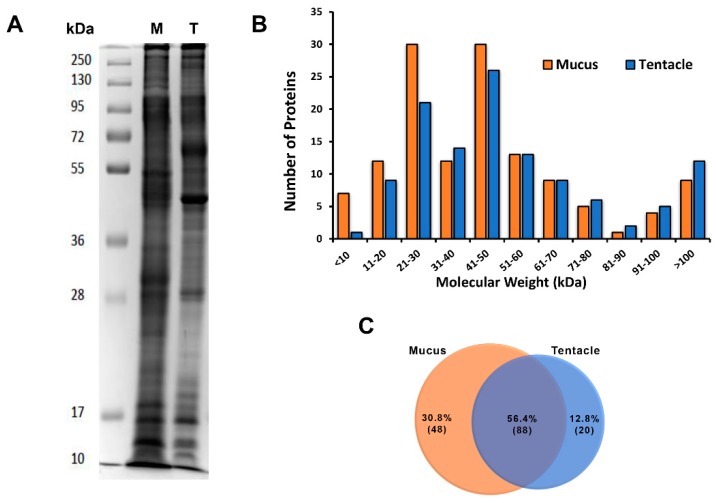
Protein complexity of the mucus and tentacle samples of *Anthopleura dowii* Verrill, 1869. (**A**) Electrophoretic profile of the mucus (M) and tentacle (T) samples analyzed with SDS-PAGE on 12% polyacrylamide gel. (**B**) Distribution of the components identified in the mucus and tentacle proteomes with respect to their molecular weight. (**C**) Venn diagram representing the distribution of the number of proteins identified (156 proteins) by LC-MS/MS in both biological samples.

Proteins obtained from the mucus and tentacles of the sea anemone were processed by triplicate using LC-MS/MS to obtain information on the venom components. The protein complexity of both samples was analyzed qualitatively in a 12% SDS-PAGE gel [49]. The electrophoretic profile of the tentacle and mucus samples showed polypeptides from 10 to more than 250 kDa (Figure 2A).

A total of 183,634 spectra were obtained and used for the identification of putative proteins related to the venom of *A. dowii*. A total of 141 polypeptides (6383 spectra) with molecular weights 5–531 kDa (Figure 2B) and two or more unique peptides in their sequence were identified in at least two of the three mucus and tentacle samples tested (Supplementary Tables S1 and S2). A manual inspection of the data was carried out by decreasing the restriction of the number of unique peptides identified by protein sequence to one, but only cases in which a single peptide showed high coverage of the identified protein were considered. This allowed us to identify a greater number of polypeptides with sequence similarity to neurotoxins that act on voltage-regulated sodium channels (Na_v_). The identification of these components was supported by the UniProt peptide search tool [50].

**Figure 3:**
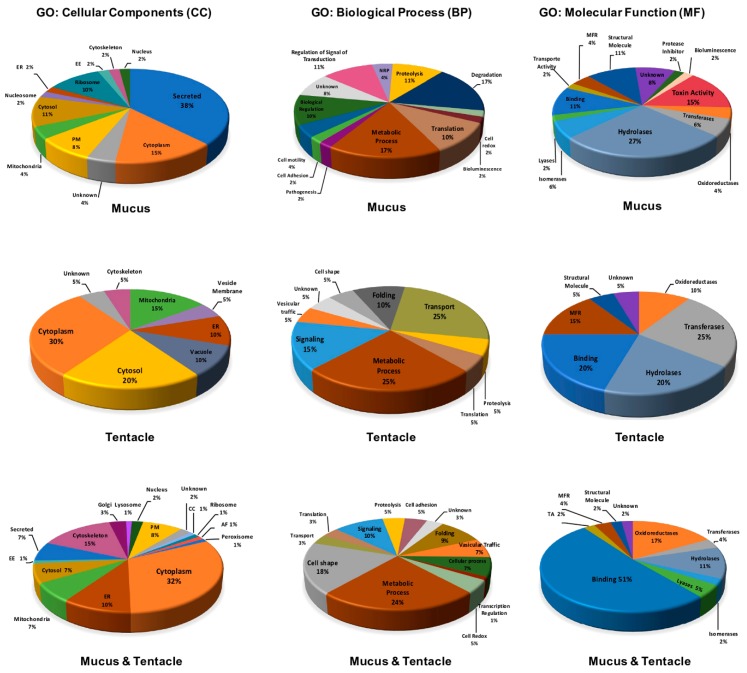
Annotation of the proteins identified in the mucus and tentacles by Shotgun-proteomics. The charts represent the relative abundance of proteins identified in the mucus (48 proteins), tentacles (20 proteins), or in both samples (88 proteins). They are classified in the three main Gene Ontology (GO) categories: cellular component, biological process, and molecular function. The annotation of the proteins is based on the amino acid sequence homology with respect to the proteins annotated in the UniProtKB database using BLASTP and the QuickGO tool.

Finally, a total of 156 proteins identified in the database were obtained, of which 56.4% were identified in both the mucus and tentacle, 30.8% were present exclusively in the mucus, and 12.8% only in the tentacle (Figure 2C). In order to associate our proteomic data with probable biological activities, the 156 previously identified proteins were compared with the protein database functionally described by UniProt/Swiss-Prot (the manually curated section) and UniProt/ TrEMBL (Computer Translation of the EMBL/GenBank nucleotide sequence data) using the BLASTP tools and peptide search [50]. In addition, we used our transcriptomic data as a database for the identification of venom components. All of the identified proteins were classified based on their amino acid sequence similarity in the GO categories “cellular components”, “biological processes”, and “molecular functions” (Figure 3).

Our results predicted a high proportion of the identified polypeptides being located in the cytoplasm, cytoskeleton, and cytosol. Several of these proteins correspond to metabolic processes, cell structure maintenance, signaling, and transport. A high proportion of different enzymes were identified in the tentacle and mucus. The highest number of proteins predicted to be localized in the extracellular space by secretion was identified in the mucus samples (38%). However, only 15% of all polypeptides identified exclusively in the mucus were related to a toxic function (Figure 3). Polypeptides, such as disulfide isomerase proteins, peptidyl-glycine, alpha-amidating monooxygenase (PAM), heat shock proteins of subfamilies 60, 70, and 90, peroxirredoxins, catalases, Cu-Zn superoxide dismutase, and Cu2+ monooxygenase, have been identified in the mucus of cnidarians [27,36,45,51] and other venomous animals, such as snakes and wasps [52,53]. The specific functions of these proteins in the venom are not known, but due to their ubiquitous character and their participation in similar processes in other non-venomous organisms, they are considered "housekeeping" proteins that participate in similar processes for many prokaryotic cells, including human cells. We identified some of these polypeptides in the tentacle and mucus proteomes, including some that were annotated as secreted proteins (Supplementary Table S1).

In order to identify the venom components, we performed a selection, considering the secreted proteins and their identification in other venomous cnidarians and non-cnidarians. Our results show 23 polypeptides related to the venom of *A. dowii*. Of the polypeptides identified as part of the venom, 19 were found in the mucus samples (Table 1); from these, 17 polypeptides were from the anemone Na^+^ channel inhibitory toxin family, the Kuniz-type/Kv2, family, protease inhibitors, chitinases, or proteases.

The tentacles represent a specialized anatomical region to capture prey and defense for sea anemones [5]. Therefore, we would expect to find toxic polypeptides in the proteome profile of the tentacles. However, none of the polypeptides identified in the tentacles were directly related to toxins. Two causes might explain the lack of toxins in our tentacle samples: (1) the greatest abundance of proteins unrelated to venom in the tentacle sample which would be hindering the identification of toxins and low abundant polypeptides by MS/MS; and (2) the tentacles might have discharged their cnida by stress manipulations [5] before the dissection. Our method to obtain the mucus was to stimulate the discharging of the cnida, identifying sequences of toxin polypeptides. Although our data showed a significant amount of “housekeeping” proteins in the mucus samples and a null identification of toxins in the tentacle proteome, our comparative strategy of protein profiles made it possible to differentiate between the proteins that could be components of venom and those that are not. This difference of polypeptides identified between one sample and another may also be due to the production of mucus with a high quantity of cnidae and diversity in interspecific competition conditions, as observed in the sea anemone *Haliplanella luciae* (currently *Diadumene lineata*) [54]. Despite the recent technological advancements in proteomic methods, transcriptomic sequencing is more efficient [55] and can identify gene sequences of toxins in the tentacles. 

The proteomic and transcriptomic profiles allowed us to obtain the first general analysis of the diversity of polypeptides in the tentacles and secretion of the sea anemone *A. dowii*. According to this information, we predict that the venom is composed of neurotoxins, enzyme inhibitors, and several hydrolase-type enzymes (Table 1), which are described below.

### 2.3. Polypeptides Related to the Venom and its Identification in the Proteome and Transcriptome of A. dowii

Here, we briefly describe the families of polypeptides directly related to the venom, its representation in the proteome and the transcriptome, and its homology with other toxins already described in other cnidarian and non-cnidarian organisms.

#### 2.3.1. Sodium Voltage-Gated (Na_V_) Channel Toxins

Among the components with probable toxic activity, there were five polypeptides identified exclusively in the mucus with a relationship in their amino acid sequence to members of the family of toxins inhibiting the Na+ channel of the sea anemone, Type I subfamily (Table 1). We identified in our transcriptome only one transcript with 100% identity with the mature toxin Delta-actitoxin-Axm1a from *Anthopleura xanthogrammica* and Delta-actitoxin-Ael1b of *A. elegantissima* (Supplementary Figure S1E). Both toxins bind specifically to site 3 of the Na_v_ channels, delaying inactivation [56]. A fourteen-residue peptide found in all of the mucus samples analyzed in this work showed 17% sequence coverage with the Delta-actitoxin-Avd1e sequence of *Anemonia viridis.* This toxin has a strong effect on crustaceans and insects (it inhibits the Na_v_ channel of *Drosophila melanogaster* (DmNa_v_1)) [57].

#### 2.3.2. Type 2 Potassium Voltage-Gated (K_v_) Channel Toxins Subfamily

Twelve transcripts encoding members of the Kunitz/Kv2 family of toxins (Supplementary Figure S1) were identified in the tentacle transcriptome, while only one member of this family was identified in the mucus proteome (Table 1). The precursors of this subfamily of K_v_ channel toxins are formed by a signal peptide and a region of the mature protein composed of 58–60 amino acid residues, including six Cys residues forming three disulfide bonds [6,9]. Among the discovered transcripts, five showed an identity in the region of the mature protein with KappaPI-AITX-Ael3a from *A. elegantissima*, ranging from 37.9–80.3%. This same group of transcripts was also compared to KappaPI-AITX-Avd3d from *Anemonia sulcata* and showed high percentages of identity in the region of the mature protein, ranging from 42.6–59.2%. Another bifunctional toxin within this family is KappaPI-SHTX-Shd2a from *S. haddoni,* to which our transcripts also showed between 54.0%–35.1% sequence identity. Protease inhibitors have been proposed to protect toxins from proteolytic degradation that could trigger a defense mechanism in their prey [58]. In the case of sea anemones, it has also been proposed that the inhibitors could function in both defensive and offensive processes [1]. Two serine protease inhibitors homologous to those in the snakes *Walterinnesia aegyptia* and *Daboia russelii* were also found in the transcriptome of *A. dowii* (Supplementary Figure S1). The PI-AITX-Axm2a and PI-AITX-Aeq3c peptides from *A. aff. xanthogrammica* and *Actinia equina*, respectively, have strong inhibitory activity on trypsin and plasmin. However, they have no activity on K^+^ channels [43,59]. We identified three transcripts with 54.2–78.3% sequence identity to the aforementioned toxins (Supplementary Figure S1). Three other transcripts showed 39.3–54.1% sequence identity to the precursors of protease inhibitors isolated from snakes, the Kunitz-type serine protease inhibitor DrKIn-II from *D. russelii* and the Kunitz-type serine protease inhibitor 1 (cVamTi) from *Vipera ammodytes ammodytes* (Supplementary Figure S1).

### 2.4. Protease Inhibitors

In addition to the inhibitors belonging to the venom Kunitz-type family, whether or not they were from the anemone type 2 K_v_ channel toxin subfamily, other types of inhibitors have been identified in sea anemones, such as elastase inhibitors and inhibitors with thyroglobulin domains type 1 [21]. Inhibitors of proteases from the serpin and Equistatin-like families were also identified in the mucus proteome and tentacle transcriptome of *A. dowii* (Table 1). Three transcripts corresponding to the family of serpins serine protease inhibitors of high molecular weight (300–500 amino acids) were identified and showed 38–41% identity to the mature protein, with members of this protein family present in the clawed frogs *Xenopus tropicalis* and *Xenopus laevis* (Supplementary Figure S2). Equistatin is a cysteine endopeptidase inhibitor that can be isolated from the total body extract of *Ac. equina* [60]. It has a sequence of 199 amino acid residues distributed in three domains of thyroglobulin type 1 [61], that are characterized by six conserved Cys residues and are related to the control of proteolytic degradation. Sequence analysis comparing Equistatin with other cysteine protease inhibitors suggests that the N-terminal domain is related to its inhibitory region, whereas the second domain is related to the inhibition of the aspartic protease and cathepsin D [62,63]. In this study, three Equistatin homologs were found in the transcriptome and one in the mucus proteome of *A. dowii* mucus proteome (Table 1 and Supplementary Figure S2): c17929_g1 only showed domains 2 and 3, c17929_g1 and c32564_g1 presented domains 1 (N-terminal domain) and 2, but domain 3 (which has an unknown function) was incomplete. None of the transcripts showed a sequence corresponding to an identified signal peptide, and the alignments showed low overall identity percentages, since they differed from Equistatin in the number of amino acids (Supplementary Figure S2).

An interesting amino acid sequence obtained from a transcript showed 40% identity with a membrane glycoprotein of 110 kDa (Supplementary Figure S2), which presented in its sequence as multiple repeats of epidermal growth factor-like and serine-protease inhibitor-like domains. This protein, called RECK (reversion-inducing cysteine-rich protein with Kazal motifs), has the ability to inhibit extracellular matrix metalloproteases (MMP-9) [64]. The negative regulation of this protein by oncogenic signals facilitates tumor invasion and metastasis. Other studies have shown that RECK can attenuate the migration of human mesenchymal stem cells (hMSCs) by mechanisms other than tumor cells [65]. The hMSCs are capable of differentiating into various cell types, representing promising tools for multiple clinical applications, including the regeneration of tissues damaged by endogenous or transplanted hMSCs.

A transcript that showed 75% identity in the region of the mature protein with PI-actitoxin-Avd5a (an elastase inhibitor) was found in our transcriptome (Supplementary Figure S2); the residues of Cys as well as those that form the reactive link were highly conserved. In addition, a peptide with Kazal domain identified in the transcriptome of *A. dowii* showed 41.5% identity with the turripeptide LoI9.1 of the sea snail *Lophiotoma olangoensis* (Supplementary Figure S2).

### 2.5. Proteases

Among the proteases identified in the proteome of *A. dowii* are metalloproteases, whose activities result from the presence of a bivalent ion, typically Zn^2+^. It has been proposed that the role of metalloproteinases in snake venom could be to facilitate the diffusion of neurotoxins through tissue damage caused by the degradation of the extracellular matrix of the prey. The venom has even been related to hemorrhagic, necrotic, inflammation, and tissue damage events observed in the prey [66,67]. We identified five families of metalloproteases in the proteome of *A. dowii.* From those, M12A (Astacin-like) and M14 (carboxypeptidase A) were only present in the mucus (Table 1), whereas M1 (aminopeptidase), M17 (aminopeptidase), and M20 (dipeptidase) were present in both the mucus and tentacles (Supplementary Table S2). Members of the M12A family have been reported in the venom of spiders of the genus *Loxosceles* [68], in the jellyfish *Stomolophus meleagris* [69] and *Chironex fleckeri* [70], and in the nematocyst content of *Nematostella vectensis* [35]. The A4 carboxypeptidases of the M14 family (with predominantly digestive functions) are secreted in soluble form after proteolytic activation, and some members of the family (M14 and M1) have been identified in the proteomes of the cnidarians *Chrysaora fuscescens* and *Hydra vulgaris* [45,71]. We suggest that M1, M17, and M20 are present in the mucus of *A. dowii* as a result of the autolysis of sea anemone cells during the process of obtaining the mucus, but are not secreted components [35].

The metalloprotease families identified in our transcriptomic data were M2, M10A, M12A, M12B, M13, M14, and M28; the most abundant enzymes were homologous to endothelin converters (ECE), which belong to the M13 family (Supplementary Table S1 and Figure S3). Another important group of transcripts were homologs of the nematocyst expressed protein 6 (NEP-6), a zinc-dependent metalloprotease of the astacin family (M12A) identified in *N. vectensis* [35]. Homologs to mammalian disintegrin and metalloproteases with thrombospondin motifs (within the M12B family) and carboxypeptidases within the M2, M14, and M28 families were also identified in the transcriptome (Supplementary Table S1 and Figure S3). Recently, transcripts encoding for members of the M12A family were identified in substantial proportions in the sea anemones S. *haddoni* and *S. helianthus* [23,25].

Members of the serine proteases family (S1 family) present in snake venoms act on components of the coagulation cascade, affecting the hemostatic system of their prey [72]. Although their role in the venom of cnidarians has not been described in detail, their proteolytic activity could be related to the activation of other proteases in the venom, the activation of toxin precursors, and the cleavage of proteins from the prey with pre-digestive purposes. Three serine proteases were identified exclusively in the mucus of *A. dowii,* and only one of them was identified in the transcriptome which showed similarity to the CUB domain (C1r/C1s, Uegf, Bone morphogenetic protein 1) and peptidase domain-containing protein 1 of the hard coral *Acropora millepora* (Table 1 and Supplementary Table S1). Other members of families S1, S8, S9B, and S10 were also identified in the transcriptome (Supplementary Table S1).

Cysteine proteases were also identified in the proteomes of *A. dowii*. Calpains are cysteine peptidases that act as heterodimers and are located intracellularly. We identified a protein like calpain-1 catalytic subunit in the mucus and tentacle, as well as in the transcriptome (Table S1).

### 2.6. Glycosyl-Hydrolases

Three members of the glycosyl-hydrolase 18 family (chitinase enzyme) and one of the glycosyl-hydrolase 20 family (beta-N-acetylhexosaminidase activity) were found exclusively in the mucus samples. Chitinases have been identified in wasp venom [73] and in the saliva of cephalopods [48,74]. However, the activity of these enzymes is related to digestive processes, and they show hemolytic activity in octopus’ saliva [74]. This could suggest that their presence in sea anemone secretions could primarily function in the extra-oral digestion of prey, with a secondary role in the mechanism of tissue or necrotic damage intoxication. Marine arthropods are constituents of the diet of sea anemones [27], and thus the role of metalloproteases and chitinases could be essential for pre-digestive purposes. No transcripts corresponding to the chitinases present in the mucus samples were identified. Another enzyme identified in the mucus and transcriptome of *A. dowii* was the beta-N-acetylhexosaminidase (Table 1 and Supplementary Figure S6). This type of protein has also been identified in the proteome of the venom of the jellyfish *Chrysaora fuscescens* [45].

### 2.7. Putative Venom Components Identified Exclusively in the Transcriptome of A. dowii.

RNAseq methods make it possible to identify putative toxins in cases in which it is difficult to obtain the venom or when some toxins are poorly represented in the venom (e.g., isoforms). Using existing information on the components of the venom of sea anemones, as well as that obtained from our data analyses, we now describe a set of sequences of peptides and proteins with pharmacological potential exclusively identified in the transcriptome of the tentacle of *A. dowii*. To reduce the potential error that could be generated by the analysis on the homology of the transcriptome, we performed a detailed analysis of the amino acid sequences predicted from the transcripts, using global alignments against functionally described protein sequences from the databases (Swiss-Prot and TrEMBL) to evaluate their percentage of global identity, rather than just local patterns. This strategy has been implemented in previous transcriptomic studies of venomous animals [75,76,77]. In the following sections, we describe these polypeptides and their homology with other toxins that have already been experimentally evaluated.

#### 2.7.1. Potassium Channel Toxins (KTx)

##### Type 1 KTx Family

Peptides belonging to the sea anemone type 1 KTx family have up to 34–37 amino acid residues and three disulfide bonds formed by six Cys residues [1,6]. Members of this toxin family have been characterized from the venom of sea anemones within the families *Actiniidae*, *Hormathiidae*, *Stichodactylidae*, and *Thalassianthidae*. The most representative member of this family is Kappa-stichotoxin-She3a (ShK), isolated from *S. helianthus*, which can block the activation and proliferation of memory T lymphocytes [78,79]. In the transcriptome of *A. dowii*, we identified a sequence (c23125_g1_i1) whose precursor showed a mature sequence of 36 amino acids, including six Cys residues, which is characteristic of this K_v_ family of toxins. Eight residues were identified between the second and third Cys, and thus this sequence can be classified as subtype 1b. The mature protein region showed only 31.6% identity to the sequence of Kappa-Styicotoxin-She3a, and 83.3% identity to the Kappa-actitoxin-Aeq4a toxins of *Ac. equina* (Table 2) and the Kappa-actitoxin-Avd6a from *An. sulcata* (Supplementary Figure S1); these proteins inhibit the Kv1/KCNA channel [80] and block the Kv1.2/KCNA2 channel [81], respectively.

##### Type 3 KTx Family

This family of toxins consists of peptides between 40 and 42 residues with three disulfide bonds. They do not show activity on the Kv1 type channel, but they block Kv3 type channels and ether-à-go-go channels (ERG, Kv10.1, hERG, Kv11.1). Some members of this KTx family even act on acid-sensitive ion channels (ASIC3), which are voltage-regulated neuronal Na^+^ channels [9,13,82,83]. The sea anemone type 3 KTx family contains a defensin-4 domain, suggesting potential antimicrobial activity. In addition to its activity, this toxin family is a blocker of the Kv3.4 channel [84] and it has also shown the capacity to decrease the cytopathic effects caused by the hepatitis virus (MHV-A59) in mouse liver cells. Another interesting feature of this toxin family is its activity on ether-á-go-go channels, which are overexpressed in most human tumors [8].

We identified 11 transcripts of this family in *A. dowii* (Supplementary Figure S1). The transcript c29930_g1_i1 showed 71.4% identity with Pi-AITX-Ael2b from *A. elegantissima* (Table 2), which potently blocks ASIC3 homotrimers and heterotrimers from the acid-sensitive ion channel containing ASIC3 (composed with isoforms of ASIC1 and ASIC2). Pi-AITX-Ael2b is in preclinical studies as a new analgesic for the treatment of chronic inflammatory pain [85]. The transcript c30503_g2_i3 showed 52.1% identity with the DeltaKappa-AITX-Avd4b from *An. sulcata* (Table 2), which inhibits Kv3 type channels (Kv3.1/KCNC1, Kv3.2/KCNC2, and Kv3.4/KCNC4) [84].

##### Type 5 Ktx Families

This family of toxins is composed of only three members: NvePTx1 from *N. vectensis*, U-MTTX-Msn2a from *Metridium senile,* and Kappa-AITX-Bcs4a from *Bunodosoma caissarum*, which has been functionally characterized. Kappa-AITX-Bcs4a inhibits the K_v_ channels Kv1.2/KCNA2, Kv1.6/KCNA6, and Kv1.3/KCNA3 in human cells [86]. Only one member of this family was identified in the transcriptome of *A. dowii* (Table 2). This sequence (c33344_g1) showed a 72% identity in the region of the mature protein with the Kappa-AITX-Bcs4a peptide and the sequence of the precursor showed a pre-pro-protein organization (Supplementary Figure S1).

#### 2.7.2. Phospholipases A2 (PLA2)

Five percent of the transcripts that we identified as components of the venom were precursors of probable PLA2 (Figure 1B). Three identified transcripts showed similarity to A2-AITX-Ucs2a from *Urticina crassicornis* [87]. The transcript c54261_g1_i1 presented 59.0% identity to A2-AITX-Ucs2a and a similar cysteine pattern (Table 3 and Supplementary Figure S7).

Three other transcripts showed similarity with the phospholipase A2-HRTX-Apt1a of the former sea anemone *Adamsia palliata* (currently *Calliactis palliata*, found in both the acontia and the tentacles) [88] and with acid phospholipase A2 D (svPLA2) of the cobra *Naja sputatrix*, which is classified as a Group I member of the vertebrate PLA2s (Supplementary Figure S7). The transcript c3686_g1_i1 showed 41.9% identity with the cobra svPLA2, which has PLA2 activity and the ability to inhibit muscarinic acetylcholine receptors (mAChR/CHRM) [89].

Interestingly, we discovered a transcript (c26312_g1) which coded for a probable PLA2, with 34.4% and 33.3% identity with members of the Group III expressed in the venomous glands of the lizard *Heloderma suspectum* and the bee *Xylocopa appendiculata circumvolans*, respectively (Table 3 and Supplementary Figure S7).

Another group of PLA2s identified was the group XII of secretory phospholipase A2; one transcript showed 31.8% identity and 64.1% similarity in the region of the mature protein with PLA2s of house mouse *Mus musculus* and *Homo sapiens* (Supplementary Figure S7). Homologs to human lysosomal phospholipase (LPLA2) with transacylase activity and PLA2, independent of calcium, were identified, showing 42% identity between their amino acid sequences and those corresponding to the mature protein [90] (Supplementary Figure S3). LPLA2 catalyzes the formation of 1-O-acyl-N-acetylsulfingosine and the concomitant release of a lysophospholipid, is classified within Group XV of PLA2, and may be present in lysosomes and in the extracellular region [91].

#### 2.7.3. Other Proteins Identified in the Transcriptome

We identified 15 transcripts that coded for probable acetylcholinesterases, some of which bore similarity to the acetylcholinesterase of the snake *Bungarus fasciatus* (Supplementary Table S1 and Figure S9). Other identified transcripts corresponded to probable phosphodiesterases, phospholipases B, lysozomal acid lipases (Supplementary Figure S9), alpha-amylases, and hyaluronidases (Supplementary Table S1).

Peptides and proteins with antibiotic properties have been identified in several species of venomous animals [92,93,94]. A group of five transcripts that coded for polypeptides sized between 232–500 amino acid residues showed similarities with members of the BPI (bactericidal permeability-increasing proteins)/LBP (lipopolysaccharide-binding proteins)/PLUNC (palate, lung, and nasal epithelium clone) family (Supplementary Figure S8). Members of the BPI/LBP family are proteins involved in host defense against bacteria and are homologous to the cholesterol ester transfer protein (CETP) and the phospholipid transfer protein (PLTP), both involved in the transport of lipids in blood plasma [95,96,97]. Our transcripts showed similarity to the human LBP, which binds to the lipid A moiety of bacterial lipopolysaccharides (LPS), a glycolipid present in the outer membrane of all Gram-negative bacteria [98].

A transcript encoding a polypeptide of 151 amino acid residues had 41.2% identity with the amino acid sequence of the bacteriolytic enzyme lysozyme c-1 from the mosquito *Anopheles gambiae* (Supplementary Figure S8) [99].

Transcripts corresponding to L-amino oxygenases (LAOO) were also identified in the transcriptome of *A. dowii* and showed 43.8% similarity to those identified in the venomous glands of the snakes *Bungarus multicinctus* and *Calloselasma rhodostoma* (Supplementary Figure S8). These enzymes catalyze the oxidative deamination of aromatic and hydrophobic L-amino acids, thereby generating hydrogen peroxide, which contributes to the various harmful effects of LAOOs, such as hemorrhage, edema, hemolysis, and apoptosis.

Other groups of peptides identified in venomous animals are type C lectins, veficolins, three-finger toxins, TCTP (translationally-controlled tumor protein), members of the CAP superfamily (Cysteine-Rich Secretory Proteins (CRISP), Antigen 5 (Ag5), and Pathogenesis-Related (PR-1)), and acrohargin I (Supplementary Figure S10). We identified 13 transcripts with close similarity to members of the CAP family. Six of them were homologous to lectin (a protein that is part of the organic matrix of the coral *Acr. millepora* [100], and the remaining seven transcripts bore similarity to five antigen allergens of the wasps *Microctonus hyperodae* and *Polistes dominula* (Supplementary Table S1).

A single transcript encoding a homologous translationally controlled tumor protein (TCTP) was identified in the transcriptome of *A. dowii* (Supplementary Figure S10). This protein is related to several biological processes, such as cell cycle progression, cell growth and differentiation, gene regulation, stress responses, and immune response [101]. The deduced amino acid sequence of one of our transcripts showed a 32–34% identity with probable three-finger neurotoxins acting on nicotinic acetylcholine receptors; however, it had two additional Cys residues, compared with Kappa-6-bungarotoxin and the Lynx1 toxins (Supplementary Figure S10).

Proteins with epidermal growth factor (EGF)-like domains and Factor 5/8 C-domain proteins have been identified in transcripts of sea anemones. Recently, it was reported that a significant number of transcripts corresponding to the 5/8 C-domain factor are present in *S. haddoni* [23], however, their presence in venom was not shown. We identified two peptides in the mucus of *A. dowii* corresponding to probable proteins with these domains; these peptides represented only 6% and 1% of coverage of the totally the amino acid sequences (Table 1). The transcriptomic data indicated the presence of transcripts that code for polypeptides with these domains (Supplementary Table S1); however, their presence in the venom and exclusively in the mucus still needs to be verified.

## 3. Materials and Methods

### 3.1. Venom and Tentacle Sample Preparation for Proteomic Analysis

Individuals of *A. dowii* were collected at low tide in the intertidal zone of the coasts of Ensenada Baja California, México. Identification of the species was previously carried out [40], examining the morphology of the polyp, cnidae, and two mitochondrial markers (partial 12S rDNA and 16S rDNA) were amplified and sequenced following standard protocols for the group [102,103]. Eight individuals of variable size were placed together in a beaker in the presence of a minimum volume of phosphate buffer saline (PBS) 1X, pH 7.0 buffer and protease inhibitors (Complete Roche) plus 1 mM dithiothreitol (DTT). The specimens were completely immersed in the buffer for 1.5 h at 4 °C, and with the help of a metal spatula, gentle rubs were made on the whole body of the anemones every 10 min (3 min of stimulation and 7 min of rest) to induce the release of secretion (mucus).

Because not all individuals exposed their tentacles, it is possible that only some of them discharged the nematocysts from this area. The agglomeration of the organisms by the restricted space, in addition to the mechanical stimulation with the spatula, generated stress, causing the anemones to discharge the nematocysts and release the venom. The secretion obtained (mucus) was frozen at −20 °C and thawed before use. The tentacles of three individuals were dissected (from 8–10 tentacles per individual) and used to extract total protein. The tissue was mixed in 2 mL of homogenization medium with protease inhibitors (400 mM mannitol, 10% (*w*/*v*) glycerol, 5% (*w*/*v*) polyvinylpyrrolidone-10, 0.5% (*w*/*v*) bovine serum albumin, 1 mM phenylmethylsulfonyl fluoride (PMSF), 30 mM Tris, 2 mM dithiothreitol, 5 mM EGTA, 5 mM MgSO_4_, 0.5 mM butylated hydroxytoluene, 0.25 mM dibucaine, 1 mM benzamidine, and 26 mM K^+^ metabisulfite, adjusted to pH 8 with NaOH) before the samples were frozen in liquid nitrogen. With the help of a pistil masher, the tissue was disbanded until a homogeneous extract was obtained. The samples were kept cold throughout the process.

The extract obtained was centrifuged at 6644× *g* for 10 min at 4 °C, and the supernatant was recovered. Part of the mucus sample was thawed and centrifuged at 8000× *g* for 10 min at 4 °C to remove the aggregates. The total protein obtained from the tentacle and mucus samples was quantified by the Bradford method [104], and the electrophoretic profile was analyzed by SDS-PAGE on 12% polyacrylamide [49]. Tentacle and mucus samples were prepared by triplicate for proteomic analysis by LC-MS/MS; each of the replicates with 40 μg of total protein was calibrated to 100 μL with sterile tetra-dialysate water and added to 100 μL buffer TE (10 mM Tris / HCl pH 7.6, 1 mM EDTA pH 8, sodium deoxycholate 0.1%), and precipitated with 100 μL TCA (trichloroacetic acid) at 72% for 3 h at 4 °C. The samples with TCA were centrifuged at 7705× *g* for 20 min at 4 °C, the supernatant was discharged, and the pellet was subjected to an additional precipitation step with 1 mL of 90% acetone and incubated for 12 h, −30 °C. Samples with acetone were centrifuged at 8436× *g* for 20 min at 4 °C, and the supernatant was discarded by aspiration. The residual acetone was removed from the pellets by vacuum drying for 20 min in a spin vacuum (Savant) without heat and then stored at −80 °C until use.

### 3.2. In solution Protein Digestion and LC-MS/MS Analysis

Previously precipitated protein samples were resuspended in 10 μL of 6 M urea buffer, reduced by the addition of reduction buffer (10 mM DTT, 100 mM ammonium bicarbonate) for 30 min at 40 °C, and subsequently alkylated by the addition of iodoacetamide, 55 mM and 100 mM ammonium bicarbonate for 20 min at 40 °C. The samples were diluted in 2 mM urea, and 10 μL of trypsin in solution was added (5 ng/μL trypsin, sequencing grade, Promega (Madison, WI, USA), 50 mM ammonium bicarbonate). The digestion was performed at 37 °C for 18 h and stopped with 5% formic acid (FA).

Before LC-MS/MS, the digested samples were resolubilized with 10 μL of formic acid (FA) 0.2% in with shaking for 15 min. Desalting of samples was carried out using C18 ZipTip pipette tips (Millipore, Billerica, MA, USA). The eluted samples were dried under vacuum and resolubilized under stirring for 15 min in 10 μL of acetonitrile 2%, formic acid (FA) 1%, and then were subsequently applied to the LC-MS system. The LC column was a C18 reversed phase column packed with a high-pressure packing cell. A 15 cm long, 75 μm i.d. Self-Pack PicoFrit fused silica capillary column (New Objective, Woburn, MA) was packed with the C18 Jupiter 5 μm 300 Å reverse-phase material (Phenomenex, Torrance, CA). This column was installed on the Easy-nLC II system (Proxeon Biosystems, Odense, Denmark) and coupled with the LTQ Orbitrap Veils (ThermoFisher Scientific, Bremen, Germany) equipped with a Proxeon nanoelectrospray ion source.

The buffers used for chromatography were 0.2% FA (buffer A) and 100% acetonitrile/0.2% FA (buffer B). During the first 12 min, 5 µL of sample was loaded into the column at a flow rate of 600 nL/min; subsequently, the gradient went from 2% to 40% buffer B in 30 min and then from 40–80% buffer B in 5 min at a flow rate of 600 nL/min. LC-MS/MS data acquisition was accomplished using a thirteen scan event cycle comprised of a full scan MS for scan event 1 acquired in the Orbitrap. The mass resolution of the MS was set to 60,000 (at *m*/*z* 400) and was used to trigger the twelve additional MS/MS events acquired in parallel in the linear ion trap for the top twelve most intense ions. The mass over charge ratio ranged from 360 to 2000 for MS scanning with a target value of 1,000,000 charges and from ~1/3 of the parent *m*/*z* ratio to 2000 for MS/MS scanning with a target value of 10,000 charges. The data-dependent scan events used maximum ion fill times of 100 ms and 1 microscan, respectively. Target ions already selected for MS/MS were dynamically excluded for 15 s. Nanospray and S-lens voltages were set to 1.5 kV and 50 V, respectively. The capillary temperature was set to 225 °C. The MS/MS conditions were a normalized collision energy of 35 V, an activation q of 0.25, and an activation time of 10 ms.

### 3.3. Peptide Identification and Functional Annotation of Proteomics Data

Tandem mass spectra were extracted using Mascot Daemon version 2.5.1. (Matrix Science, London, UK). Charge state deconvolution and deisotoping were not performed. All MS/MS spectra were analyzed using Mascot. Mascot was set up to search the NCBI_Sea_Anemone_txid6103 (Order Actiniaria) database (unknown version, 52295 entries), assuming the digestion enzyme trypsin. Mascot was searched with a fragment ion mass tolerance of 0.60 Da and a parent ion tolerance of 10.0 PPM. O+18 of pyrolysine and carbamidomethyl of cysteine were specified in Mascot as fixed modifications. The oxidation of methionine was specified in Mascot as a variable modification.

Scaffold (version Scaffold_4.8.3, Proteome Software Inc., Portland, OR, USA) was used to validate the MS/MS based peptide and protein identifications. Peptide identifications were accepted if established at a probability greater than 95.0%. Protein identifications were accepted if established at a probability greater than 99.0% and contained at least two or more unique peptides in at least two of three biological replicates, unless otherwise stated peptides. Protein probabilities were assigned by the Protein Prophet algorithm [105] with Scaffold delta-mass correction. Proteins that contained similar peptides and could not be differentiated based on MS/MS analysis alone were grouped to satisfy the principles of parsimony.

To improve the annotation of the identified proteins, the manual curation of each was carried out with the UniProtKB database (https://www.uniprot.org/; period June–July 2018). The functional assignment, the cellular location and the biological processes related to the identified proteins were obtained from UniProt using the QuickGO Gene Ontology online program (https://www.ebi.ac.uk/QuickGO/;GO version: 2018-08-05). For some sequences corresponding to peptide toxins, the restriction was modified to a single peptide present in two of the three biological replicates, thus maintaining the percentage probability of identification for peptides and proteins established for Scaffold. Additionally, the proteins identified in the proteomes that related to components of the venom were compared with the sequences of proteins obtained from the tentacle transcriptome of *A. dowii*.

### 3.4. Functional Annotation of Assembled Transcriptome and Identification of Genes of Putative Toxins

In a previous publication [44], we reported the assembly of transcriptome from the tentacles of two specimens of *A. dowii*. The length distribution from the assembly was analyzed, and sequences smaller than 227 bp were discarded. After this filter, 62,880 contigs with sequence lengths between 227–15,115 bp were maintained. These contigs were used to perform the functional annotation using the Trinotate software (https://trinotate.github.io/) [106], and their similar sequences searched with BLASTX (https://blast.ncbi.nlm.nih.gov/Blast.cgi) against the protein databases UniProt and RefSeq with an e-value cut-off of 1E-5. The prediction of the open reading frames (ORFs) and the amino acid sequences corresponding to the annotated transcripts were obtained with TransDecoder (http://transdecoder.github.io/). The amino acid sequences of the ORFs were used to identify protein domains in the Pfam database (http://pfam.xfam.org). The prediction of signal peptides, propeptides, and transmembrane domains was made with SignalP (http://www.cbs.dtu.dk/services/SignalP/), ProP (http://www.cbs.dtu.dk/services/ProP/) and tmHMM (http://www.cbs.dtu.dk/services/TMHMM/), respectively. Classification of the contigs into the different Gene Ontology (GO) categories was completed with the program Blast2GO and the information obtained from the UniProt/Swiss-Prot and RefSeq databases, and the results were plotted using the WEGO (Web Gene Onthology Annotation Plot) program (http://wego.genomics.org.cn/).

The transcripts that coded for putative components from the venom were selected from our functional annotation, considering the keywords of the BLASTX outputs, such as toxin, venom, and nematocyst. The amino acid sequences of these transcripts were manually reanalyzed with PSI-BLAST (Position-Specific Iterative Basic Local Alignment Search Tool), comparing them with the UniProt/Swiss-Prot database. Polypeptides with sequence similarity with components of the venom of cnidarians or other phyla were classified in families of putative toxins or components of the venom according to the prediction by sequence homology in the databases (UniProt/Swiss-Prot). The relative abundances of the transcripts calculated for each component of the venom were quantified and graphically represented in Origin (OriginLab Software). The sequences of the putative components of the *A. dowii* venom were aligned in ClustalX 2.1 against the sequences that showed the best score, the lowest e-value, the highest local identity percentage, and the highest percentage of coverage obtained in the alignments local with BLASTP. The global percentages of similarity and identity for the alignments made in ClustalX 2.1 were calculated with the Lalign program, considering only the mature region of the peptides and proteins encoded by the selected transcripts (https://embnet.vital-it.ch/software /LALIGN_form.html) [107].

## 4. Conclusions

In general, we observed that the quantity, and especially the diversity, of probable toxins in our transcriptome was considerably greater than that found in the mucus proteome, a similar result found in other species of sea anemones [23] and other venomous animals [45,75]. These differences can be a consequence, for example, of not all transcripts being translated into proteins and some toxins having a very low representation and/or proportion in the venom, preventing them from being detected by proteomic methods. This could explain the low presence of the Kuniz-type/Kv2 subfamily toxins and the null detection of subfamilies 1, 3, and 5 in the mucus. However, these four families of toxins, which act on K^+^ channels, were the most abundant group of neurotoxins identified in the tentacle transcriptome of *A. dowii* (Figure 1B, Supplementary Table S1 and Figure S1). Although several families of metalloproteases were identified in the proteomes, the most abundant group of proteases in the transcriptome (40.5%, 30 transcripts) was represented by members of the M13 family (Supplementary Table S1), which were not identified in the proteomes.

Some toxins identified in the mucus proteome (e.g., most NaTx and chitinases) were not identified in the transcriptome. Our transcriptomic data identified a single transcript encoding a member of the anemone NaTx inhibitory toxin family (the type I subfamily), which maintains 100% identity in the region of the mature peptide with the Delta-actitoxin-Axm1a of *A. xanthogrammica* (Table 1 and Supplementary Figure S1). Members of this family of neurotoxins have been identified by immunolocalization in secretory cells other than nematocytes [108], and transcripts corresponding to this toxin have also been identified in tissues other than the tentacles in other anemone species [23,109,110].

The differential expression of venom components depending on the function of each anatomical region has been well documented for some species of sea anemones [22,109,110] and could explain why the transcripts corresponding to the NaTx and chitinases of the mucus were not identified in the tentacle transcriptome. The complexity, per se, that represents the venom production system in sea anemones is a fundamental factor that must be considered in the design of experimental strategies and analyses. Consideration of this factor will allow us to differentially explore the components of the venom and will provide information on their role in venom production.

Bioprospecting of the components of sea anemone venom using proteomic or transcriptomic tools has increased in recent years, however, only in a few cases, both methods have been used to explore the toxic components of sea anemone venom [23,39]. Our strategy included a proteomic comparison of components in the tissue and secretions of *A. dowii*, which were compared with the information generated in the transcriptome of the tentacle, so that a better identification of the components of the venom could be made. Our data allowed us to suggest that the venom of *A. dowii* is made up of toxins that act on voltage-regulated Na^+^ and K^+^ channels, cytotoxins, and a diverse group of hydrolases (proteases, chitinases, and phospholipases). In addition, our transcriptomic data consisted of a set of transcripts that coded for peptides and proteins, which could be potential candidates in the design of therapeutic and biotechnological tools.

## Figures and Tables

**Table 1:** Venom components identified in the proteomes of the sea anemone mucus and tentacles.

Putative Conserved Domain Detected ^a^	Uniprot Accession ^b^	Protein Name ^c^	ID-Transcript	Sample M/T ^d^	Organism	% Coverage M/T ^e^
**Sea anemone sodium channel inhibitory toxin family. Type I subfamily**	P0DL52	Delta-actitoxin-Avd1e 1	Unidentified	*M/-*	*Anemonia viridis*	17/-
	P0C1F0	Delta-actitoxin-Ael1b	Unidentified	*M/-*	*Anthopleura elegantissima*	100/-
	P0C5G1	Delta-actitoxin-Axm1f	Unidentified	*M/-*	*Anthopleura xanthogrammica*	45/-
	P0C1F1	Delta-actitoxin-Ael1c	Unidentified	*M/-*	*Anthopleura elegantissima*	100/-
	P01530	Delta-actitoxin-Axm1a	c22149_g1	*M/-*	*Anthopleura xanthogrammica*	29/-
**Venom Kunitz-type family. Sea anemone type 2 potassium channel toxin subfamily**	P86862	KappaPI-actitoxin-Ael3a	c14874_g1	*M/-*	*Anthopleura elegantissima*	26/-
**Serpin family. Ov-serpin subfamily**	Q52L45	Leukocyte elastase inhibitor	c27265_g1	*M/-*	*Xenopus laevis*	3/-
**Thyroglobulin_1**	P81439	Equistatin	Unidentified	*M/-*	*Actinia equina*	9/-
**Aerolysin family**	P09167	Aerolysin-like	Unidentified	*M/-*	*Aeromonas hydrophila*	1/-
**Peptidase S1 family**	A7RW61	Predicted protein	Unidentified	*M/-*	*Nematostella vectensis*	5/-
	A7S5Y0	Predicted protein	c28983_g1	*M/-*	*Nematostella vectensis*	7/-
	B8V7S0	CUB and peptidase domain-containing protein 1	Unidentified	*M/-*	*Acropora millepora*	3/-
**Peptidase_M12A**	A7SQR7	Metallo- endopeptidase	Unidentified	*M/-*	*Nematostella vectensis*	3/-
**Peptidase_ M14**	T2M3L7	Carboxypeptidase A4	c39288_g1	*M/-*	*Hydra vulgaris*	7/-
**Glycosyl hydrolase 18 family**	A0A1T4JGY1	Chitinase-C enzyme	Unidentified	*M/-*	*Nematostella vectensis*	8/-
	A0A1T4JH12	Chitinase-A enzyme	Unidentified	*M/-*	*Nematostella vectensis*	15/-
	A0A1T4JH12	Chitinase-A enzyme	Unidentified	*M/-*	*Nematostella vectensis*	15/-
**Glycosyl hydrolase 20 family**	A7RSQ4	Predicted protein	c30078_g1_i1	*M/-*	*Nematostella vectensis*	3/-
**Factor 5/8 C-Domain Proteins**	A7RK24	Predicted protein	Unidentified	*M/-*	*Nematostella vectensis*	6/-
**Peptidase_M1**	A7RUV9	Aminopeptidase	Unidentified	*M/T*	*Nematostella vectensis*	3/3
**Peptidase_M17**	A7SGM8	Predicted protein	c27931_g1_i1	*M/T*	*Nematostella vectensis*	8/6
**Peptidase_M20**	A7RZC0	Predicted protein	c31795_g1_i2	*M/T*	*Nematostella vectensis*	11/9
**Peptidase C2 family**	T1E719	Calpain-1 catalytic subunit-like protein.	c27362_g1_i1	*M/T*	*Crotalus horridus*	5/5

^a, b, c^ Data obtained from the UniprotKB database. ^d,e^ M = mucus, T = tentacle, M/- = only present in mucus, and M/T = present in mucus and tentacle. % coverage = amino acid residues identified in a protein sequence that were detected by MS in the sample.

**Table 2:** Putative neurotoxins identified exclusively in the transcriptome of *A. dowii* Verrill, 1869.

ID-Transcript	Aminoacid SEQUENCE ^a^	E-value PSI-BLAST	Protein Identity (%) ^b^	Protein Family	UniProtKB Accession
c23125_g1_i1	MNSKLVIVFLLCAILVVSVTS *RRVRTWDDFERDQDYEEEPAPYGKR* **ACKDNYSAATCKDVKKNNNCGSEKYATNCAKTCGKC**	2e-14	Kappa-actitoxin-Avd6a (83.3)	Sea anemone type 1 potassium channel toxin family. Type 1b subfamily.	Q9TWG1
c29930_g1_i1	MSYQRFLFLVVVASLIATSLA *VPKDLEER* **GTTCSCGNTKGIYWFFLKTCPSDRGYTGSCNYFFGICCYPVD**	1e-17	Pi-AITX-Ael2b (71.4)	Sea anemone type 3 (BDS) potassium channel toxin family.	P61542
c30503_g2_i5	MAAKSVLMMLAIFMALLLLANG *EEAQGEVRIKAR* **ALSCNCGKEDNAPSGDWWLWRSSCPGGYGYTSSCNAGFGNICCLPRG**	7e-06	DeltaKappa-AITX-Avd4b (52.1)	Sea anemone type 3 (BDS) potassium channel toxin family.	P59084
c33344_g1	MKTLVVFLVVAVIVVNA *YRIKEEYEDEMAPELERRA* **CKKKWNECTRDSDCCDEKGWANQKLQCLQQCDEGGCLEYRQCLFHSGLQRK**	3e-20	Kappa-actitoxin-Bcs4a (72.0)	Sea anemone type 5 potassium channel toxin family.	C0HJC4

^a^ Amino acid sequence corresponding to the precursor, predicted from the cDNA sequence. The signal peptide region is underlined, the propeptide is shown in cursive letters, and the mature peptide in bold. ^b^ The percentage of identity was calculated with the LALIGN Server program, and only the region of the mature peptide was considered in the calculation.

**Table 3:** Putative phospholipases A2 identified exclusively in the transcriptome of *A. dowii* Verrill, 1869.

ID-Transcript	Aminoacid Sequence ^a^	E-value PSI-BLAST	Protein Identity (%) ^b^	Protein Family	UniProtKB Accession
c54261_g1	MMMMKKKSTTTLIVLLGMAFLVEG *LSLNNLEDDKRMNVKTGDGRAEKR* **NLWQFGNMIKCATGRDAGDYNGYGNYCGWGGSGVPVDGVDRCCQAHDRCYDNHDSCNPKTNYYSYSKSGKHPSCTISCGDSTQNDQCERNVCSCDKVAAECFARNNYNNANKH**	6e-57	A2-AITX-Ucs2a (59.0)	Phospholipase A2 family.	A7LCJ2
c3686_g1_i1	MGALKLLVLLAVVACVAC *TSLDLGKLKKKSLSKALKTQVHTRARR* **SLYEFYKMITCETGRSWQDYNLYGCFCGKGGTGTPVDALDQCCFDHDECYSQAAASVCPWPLQIYLDSYWHKNCSECDASKNSACEQALCECDSKAARCFKNNKWDPQYDDYPQEKCA**	3e-22	Neutral phospholipase A2 muscarinic inhibitor (41.9)	Phospholipase A2 family. Group I subfamily.	Q92084
c26312_g1	MDSYVSKIFVILAVILHASLCQA *MYDWKTKTFIR* **KDNSKLVVPGTKWCGKGNNAMSFDDLGEHRETDLCCREHDHCPTYILPFQRRFGILNLYPSHLSLCSCEMKLYNCLWNVTSHVAVAVGRMYFNVLRVPCFHLVEKKVCKERSFDWWKFKYVCKKYGVEVKGQTFMPKRFHKQLQVQPSNWNATANGTM**	1e-30	Phospholipase A2 isozymes PA3A/PA3B/PA5 (34.4)	Phospholipase A2 family. Group III subfamily.	P16354

^a^ Amino acid sequence corresponding to the precursor, and predicted from the cDNA sequence. The signal peptide region is underlined, the pro-peptide is shown in cursive font and the mature peptide is shown in bold. UniProtKB Accession numbers used for GO-analysis. ^b^ Percentage of identity was calculated with the LALIGN Server program, and only the region of the mature polypeptide was considered in the calculation.

## Supplementary Materials

The following are available online at https://www.mdpi.com/1660-3397/17/8/436/s1, Figure S1–S10: Multiple alignments of amino acid sequences corresponding to putative components of the venom of *Anthopleura dowii* Verrill, 1869, Table S1: Sequences and proteins and peptides annotation in the tentacle transcriptome, and in the tentacle and mucus proteomes of *Anthopleura dowii* Verrill, 1869, Table S2: Report of protein samples MS/MS data of the mucus and tentacle.
