# Supplementary material for: Transcriptomic and Proteomic Analysis of the Tentacles and Mucus of Anthopleura dowii Verrill, 1869
Source: Mar Drugs. 2019 Jul 25;17(8):436. doi: 10.3390/md17080436 (PMC6722582; doi:10.3390/md17080436)
Supplement: Supplementary file 1 [file marinedrugs-17-00436-s001.zip › Figure S1-S10.pdf]

# Supplementary information: Transcriptomic and Proteomic Analysis of Tentacles and Mucus of *Anthopleura dowii* Verrill, 1869.

**Santos Ramírez-Carreto [1], Rosario Vera-Estrella [2], Tobías Portillo-Bobadilla [3], Alexei Licea-Navarro [4], Johanna Bernaldez-Sarabia [4], Enrique Rudiño-Piñera [1], Jerome J. Verleyen [5], Estefanía Rodríguez [6] and Claudia Rodríguez-Almazán [1,*]**

[1] Departamento de Medicina Molecular y Bioprocesos, Instituto de Biotecnología, Universidad Nacional Autónoma de México, Avenida Universidad 2001, Cuernavaca, Morelos 62210, México; santos@ibt.unam.mx (S.R.-C.); rudino@ibt.unam.mx (E.R.-P.); claudiar@comunidad.unam.mx (C.R.-A.).

[2] Departamento de Biología Molecular de Plantas, Instituto de Biotecnología, Universidad Nacional Autónoma de México, Avenida Universidad 2001, Cuernavaca, Morelos 62210, México; rosario@ibt.unam.mx (R.V.-E.)

[3] Unidad de Bioinformática, Bioestadística y Biología Computacional. Red de Apoyo a la Investigación, Coordinación de la Investigación Científica, Universidad Nacional Autónoma de México-Instituto Nacional De Ciencias Médicas y Nutrición Salvador Zubirán, Calle Vasco de Quiroga 15, Tlalpan, C.P. 14080, Ciudad de México, México. tobias@cic.unam.mx (T.P.-B.)

[4] Departamento de Innovación Biomédica, CICESE, Carretera Ensenada-Tijuana 3918, Ensenada, BC C.P. 22860, México; alicea@cicese.mx (A.L.-N.); jbernald@cicese.edu.mx (J. B.-S.)

[5] Unidad Universitaria de Secuenciación Masiva y Bioinformática, Instituto de Biotecnología, Universidad Nacional Autónoma de México, Avenida Universidad 2001, Cuernavaca, Morelos 62210, México; jerome@ibt.unam.mx (J.J.V)

[6] Division of Invertebrate Zoology, American Museum of Natural History, Central Park West at 79th Street, New York NY 10024, USA. erodriguez@amnh.org (E.-R.)

## Alignments multiple of amino acid sequences corresponding to precursors from putatives venom components inferred from the transcripts obtained from the tentacles of *A. dowii*.

In all the alignments, the region corresponding to the signal peptide is highlighted in orange, in green is highlighted the region of propeptide and in blue is highlighted the region of mature protein. In some cases, the conserved Cys residues are highlighted in bold blue and in bold letters. The number of amino acids (aa) and the percentages of identity (I%) are calculated considering only the regions of the mature protein, this last value is calculated with the program LALIGN version 2.1.30 and with respect to the sequence indicated with 100% identity in the alignment. In some cases, the percentage of similitude (%S) also is showed, this value also calculated with the program LALIGN version 2.1.30 and considering only the regions of the mature protein and with respect to the sequence indicated with 100% similarity in the alignments. All alignments were made with ClustalX 2.1 and the characters (*), (:) and (.) are shown in the low region along the alignments to indicate positions with fully conserved residues, positions with conservative substitutions and positions with less conservative substitutions, respectively.

### A) Sea anemone type 1 potassium channel toxin family. Type 1b subfamily.

```
                                                                                                        PSI-BLAST
                                                                                           aa   %I      E-value
c23125_g1_i1     MNSKLVIVFLLCAILVVSVTSRRVRTWDDFERDQDYEEEPAPYG--KRACKDNYSAATCKDVKKNNNCGSEKYATNCAKTCGKC   36   100
Kappa-AITX-Aeq4a ---------------------------------------------------GCKDNFSANTCKHVKANNNCGSQKYATNCAKTCGKC   36   83.3    4e-14
Kappa-AITX-Avd6a ---------------------------------------------------ACKDNFAAATCKHVKENKNCGSQKYATNCAKTCGKC   36   83.3    2e-14
U-AITX-Avd11a    --------------LVVSVTSRRVRFWDDFERDENFEEERPYLRNVKRACNDYKSSSYCRSVGSRNECGIHKYRMYCRKTCGSC   36   47.2    8e-20
                                                                .*:*  ::  *: *  .::** .**   * ****.*
```

## B) Venom Kunitz-type family, Sea anemone type 2 potassium channel toxin subfamily.

### B.1)

| | | aa | %I | %I | %I | %I | %I | PSI-BLAST E-value | PSI-BLAST E-value | PSI-BLAST E-value | PSI-BLAST E-value | PSI-BLAST E-value |
|---|---|---|---|---|---|---|---|---|---|---|---|---|
| **c17454_g1_i1** | ---MMKTLFLMSLAMLVAVTMARPDS------FCFLAPEVGKCRGHIRRYYYSTETGKCQQFFYGGCGGNRNNFQSEDECRATCYPREK----------- | 61 | **100** | 42.9 | 44.3 | 37.5 | 50.8 | | ----- | ----- | ----- | ----- |
| KappaPI-SHTX-Shd2a | ---MAKLYFLLCLALVACLTMATEEMPA----LCHLQPDVPKCRGYFPRYYYNPEVGKCEQFIYGGCGGNKNNFVSFEACRATCIIPL------------ | 62 | 54.0 | 51.6 | 48.4 | 35.1 | 41.9 | 3e-21 | 6e-20 | 1e-17 | 5e-12 | 1e-12 |
| **c27319_g1_i5** | -MSLQKKLLFATVVITACLFMRTVDGKPSRKKLCYLEPDSGNCYGYFPSYYYNPSSGRCENFTYGGCGGNANNFSTLKSCKAVCRAKK------------ | 62 | 42.9 | **100** | 46.8 | 43.2 | 50.0 | ----- | | ----- | ----- | ----- |
| **c14257_g1_i1** | MKKMRSVTILLSIVVFSGAFAARPEY-------CNLPYETGKCMAYIPSYYFDPSTGTCDQFIYGGCGGNKNNFDTLAACEKTCMV-------------- | 58 | 44.3 | 46.8 | **100** | 36.1 | 48.3 | ----- | ----- | | ----- | ----- |
| **c14874_g1_i1** | MAKSATLMVFLLCFFLVADVSYGINS------ICLLPKKQGFCRARFPRFYYNSSTRRCEMFYYGGCGGNANNFNTLKECQEVCLGYREIVFPGDSPKEN | 71 | 37.5 | 43.2 | 36.1 | **100** | 52.1 | ----- | ----- | ----- | | ----- |
| KappaPI-AITX-Ael3a | -----------------------INS------ICLLPKKQGFCRARFPRFYYNSSTRRCEMFYYGGCGGNANNFNTLEECEKVCLGY------GEAWKAP | 65 | 37.9 | 48.5 | 42.4 | 80.3 | 55.4 | 1e-12 | 2e-14 | 2e-14 | 4e-36 | 4e-21 |
| KappaPI-AITX-Avd3d | -----------------------ING------DCELPKVVGRCRARFPRYYYNLSSRRCEKFIYGGCGGNANNFHTLEECEKVCGVR--------S---- | 59 | 42.6 | 51.6 | 50.0 | 59.2 | 50.8 | 4e-12 | 2e-15 | 2e-16 | 5e-23 | 4e-16 |
| **c10_g1_i1** | ---MKDIFLTCVILFLGLSLTSARED------ICLLPKKVGFCRALIPRFYFNSKTRRCEGFNYGGCFGNKNNFISIKDCQKACM--------------- | 56 | 42.6 | 38.7 | 48.3 | 52.1 | **100** | ----- | ----- | ----- | ----- | |
| KIn-II | ---MSSGCLLLLLGLLTLWAELTPVSG--RPRLCELPAESGLCNAYIPSFYYNPHSHKCQKFMYGGCGGNANNFKTIDECHRTCVG-------------- | 57 | 47.5 | 50.0 | 53.4 | 40.8 | 45.6 | 1e-13 | 9e-17 | 2e-19 | 6e-17 | 4e-16 |
| Kunitz inhibitor IV | ---MSSGGLLLLLGLLTLWAELTPISGQDRPKFCHLPVDSGICRAHIPRFYYNPASNQCQGFIYGGCGGNANNFETRDQCRHTCGGK------------- | 60 | 50.8 | 50.0 | 48.3 | 41.1 | 46.7 | 4e-17 | 1e-14 | 1e-16 | 9e-13 | 2e-11 |
| | * * * . : :*:. *: * **** ** *** : *. .* | | | | | | | | | | | |

### B.2)

| | | aa | %I | %I | PSI-BLAST E-value | PSI-BLAST E-value | PSI-BLAST E-value |
|---|---|---|---|---|---|---|---|
| PI-AITX-Axm2a | -----------------------------------APVNEDCLLPKKVGPCRAAVPRFYYNSDSGKCEGFTYGGCHANANNFKTKDECKNACH-- | 58 | **100** | 72.4 | 5e-31 | 8e-26 | 1e-20 |
| PI-AITX-Aeq3c | -----------------------------------YPANEGCLLPMKVGPCRAAWPSYYYNSKSEKCEEFTYGGCDANANNFQTEEECKKAC--- | 57 | 72.4 | **100** | 3e-27 | 5e-27 | 4e-19 |
| **c27319_g1_i1** | MQKSNGINVKTVLLLAALFCAFILILGLSLSKTAGAPANKDCLLPRMVGPCRAAIPRFYYNSKSEKCEGFTYGGCDANANNFKTEEECKNKACHAA | 60 | 78.3 | 75.0 | | ----- | ----- |
| **c27319_g1_i4** | ----------MSLQKKLLFATVVITACLFLSKISGAAANDDCHLPMKVGLCRASIPRFYFNSKSEKCERFTYGGCDANANNFKTEEECKEACHAT | 57 | 68.3 | 70.0 | ----- | | ----- |
| **c27319_g1_i3** | ----------MSLQKKLLFATVVITACLFLSNISGAAAN-VCLLPKVVGHCQAVIPRFYFNSKSEKCERFNYGGCGGNGNNFQTKDQCTNACTT- | 55 | 59.3 | 54.2 | ----- | ----- | |
| | ..* * ** ** *:* * :*:**.* *** *.**** .*.***:*:::*.:** | | | | | | |

### B.3)

| | | aa | %I | %I | PSI-BLAST E-value | PSI-BLAST E-value | PSI-BLAST E-value |
|---|---|---|---|---|---|---|---|
| cVamTi | MSSGGLLLLLGLLTLWAELTPVSGQDHPKFCYLPADPGRCKAHIPRFYYDSASNKCNKFIYGGCPGNANNFKTWDECRQTCGASAMGRPT | 61 | **100** | 57.4 | 1e-19 | 8e-17 | 2e-18 |
| DrKIn-II | MSSGGLLLLLGLLTLWAELTPISGHDRPTFCNLAPESGRCRAHLRRIIYNLESNKCEVFFYGGCGGNDNNFSTWDECRHTC----VGK-- | 60 | 57.4 | **100** | 8e-12 | 9e-18 | 2e-15 |
| **c10125_g1_i1** | MVKQMIFVLF--LAMVVGLS----MARPEFCLLPAKPGRCKAAFKRYYFDKSIGACRQFIYGGCKGNRNNFKSKEKCDRICGARR------ | 59 | 52.5 | 39.3 | | ----- | ----- |
| **c10208_g1_i1** | ------------------------MARPKYCLLPPKVGLCMASMRRYYYEKSIAKCSQFFYGGCGGNKNNFKTMDECVKTCALL------ | 58 | 50.8 | 53.3 | ----- | | ----- |
| **c10208_g1_i2** | ------------------------MARPKYCLLPAKIGRCRARILRYYYDKSNAKCSQFYYGGCGGNRNNFETRHECVKTCALL------ | 58 | 54.1 | 50.0 | ----- | ----- | |
| | :* :* *... * * * : * *:: * * **** ** ***.: .:* : * | | | | | | |

## C) Sea anemone type 3 (BDS) potassium channel toxin family.

### C.1)

| | | aa | %I | %I | %I | PSI-BLAST E-value | PSI-BLAST E-value | PSI-BLAST E-value | PSI-BLAST E-value | PSI-BLAST E-value |
|---|---|---|---|---|---|---|---|---|---|---|
| DeltaKappa-AITX-Avd4b | ----------------------------------AAPCFC--PGKPDRGDLWILRGTCPGGYGYTSNCYKWP-NICCYPH-- | 43 | **100** | 95.3 | 47.8 | 8e-06 | 2e-08 | 9e-07 | 7e-06 | 2e-08 |
| DeltaKappa-AITX-Avd4a | ----------------------------------AAPCFC--SGKPGRGDLWILRGTCPGGYGYTSNCYKWP-NICCYPH-- | 43 | 95.3 | **100** | 47.8 | 1e-05 | 2e-08 | 1e-06 | 2e-05 | 5e-08 |
| PhcrTx2 | ----------------------------------ALPCRC--EGKTEYGDKWIFHGGCPNDYGYNDRCFMKPGSVCCYPKYE | 46 | 47.8 | 47.8 | **100** | 2e-05 | 5e-08 | 5e-10 | 1e-04 | 2e-03 |
| **c30503_g2_i2** | MAAKSVLMMLAIFMALAFLANGEE---EVRVNVRAIRCNCIGKDQGPYGDWWFWRGSCPDGYGYKTSCMLGID-ICCFPRA- | 43 | 43.5 | 45.7 | 39.6 | | ----- | ----- | ----- | ----- |
| **c30503_g2_i4** | MAAKSVLMMLAIFMALLLLANGEEAQGEVRIKARALLCNCEGKASS--GDWWFWRGSCPDGYGYKTSCMLGID-ICCFPRA- | 44 | 47.7 | 47.7 | 43.5 | ---- | | ----- | ----- | ----- |
| **c30503_g2_i3** | MAAKSVLMMLAIFMALLLLANGEEAQGEVRIKARALLCNC--EGKASSGDWWFWRGSCPDGHGYTSSCSAGLGSICCLPKT- | 45 | 48.9 | 48.9 | 47.8 | ---- | ----- | | ----- | ----- |
| **c30503_g2_i5** | MAAKSVLMMLAIFMALLLLANGEEAQGEVRIKARALSCNCGKEDNAPSGDWWLWRSSCPGGYGYTSSCNAGFGNICCLPRG- | 47 | 52.1 | 46.8 | 37.5 | ---- | ----- | ----- | | ----- |
| **c22963_g1** | MAAKSILMMLAICMALVFLTSGEEAQEDARVKPRAVSCYCGRYDKAPYGDWWFMRGSCPGGYGYTRSCQTGMN-VCCFPKA- | 50 | 45.1 | 47.8 | 41.7 | ---- | ----- | ----- | ----- | |
| | * * * . ** *: :. **..:**. * :** *: | | | | | | | | | |

### C.2)

| | | aa | %I | %I | %I | PSI-BLAST E-value | PSI-BLAST E-value | PSI-BLAST E-value | PSI-BLAST E-value | PSI-BLAST E-value | PSI-BLAST E-value |
|---|---|---|---|---|---|---|---|---|---|---|---|
| Kappa-AITX-Ael2a | ------------------------------------GTTCYCGKTIGIYWFGTK-TCPSNRGYTGSCGYFLGICCYPVD- | 42 | **100** | 64.3 | 50.0 | 3e-10 | 2e-15 | 0.020 | 0.079 | 5e-03 | 0.58 |
| Pi-AITX-Ael2b | ------------------------------------GTACSCGNSKGIYWFYRP-SCPTDRGYTGSCRYFLGTCCTPAD- | 42 | 64.3 | **100** | 45.2 | 1e-17 | 3e-15 | 1e-03 | 0.075 | 8e-06 | 0.031 |
| Pi-SHTX-Hcr5d | ------------------------------------GTPCDCYGYTGVYWFMLS-RCPSGYGYNLSCHYFMGICCVKR-- | 41 | 50.0 | 45.2 | **100** | 1e-08 | 9e-09 | 1e-05 | 2e-05 | 3e-07 | 8e-05 |
| **c29930_g1_i1** | MSYQRFLFLVVVASLIATSLAVPKDLE-------ERGTTCSCGNTKGIYWFFLK-TCPSDRGYTGSCNYFFGICCYPVD- | 42 | 81.0 | 71.4 | 52.4 | | ----- | ----- | ----- | ----- | ----- |
| **c29930_g1_i2** | MSYQRFLFLVVVASLIATSLAVPKDLE-------ERGTSCRCGSIKGIYWFYLR-SCPSNHGYTGKCDYFMGICCYPAD- | 42 | 66.7 | 66.7 | 52.4 | ----- | | ----- | ----- | ----- | ----- |
| **c13634_g1_i1** | MSYERLFCLVMVAALVATSAVHSQEVEK-DDKLQERAVSCLCESRSGDYWFMKL-GCPDGYGYFKHCRSTLGICCLKWKG | 43 | 37.2 | 37.2 | 44.2 | ----- | ----- | | ----- | ----- | ----- |
| **c13634_g1_i2** | MSYERLFCLVMVAALVATSAVHSQEVEK-DDKLQERAISCLCESRSGDYWFMKL-GCPDGYGYFKHCSTAAGICCVKYP- | 42 | 35.7 | 33.3 | 47.6 | ----- | ----- | ----- | | ----- | ----- |
| **c5043_g1** | MSYQRVLCLVVAVGLIAFAVAFPKDAALMEDDMLMKRVSCSCYGNSGTYWLMR--GCPSGHGYKDSCRYLLGKCCVTVQL | 41 | 41.9 | 44.2 | 48.8 | ----- | ----- | ----- | ----- | | ----- |
| **c50300_g1** | MS-SRFLFLVVLTVLIAASMALDYDE---EKGFMVKRVGCECHGHSGTYWFLHQAGCPGGYKYKGGCMYLAGRCCYK--- | 40 | 37.2 | 34.9 | 42.9 | ----- | ----- | ----- | ----- | ----- | |
| | * * * **: ** . * * * ** | | | | | | | | | | |

## D) Sea anemone type 5 potassium channel toxin family.

```
                                                                                                                        PSI-BLAST
                                                                                                          aa    %I      E-value
c33344_g1          MKTLVVFLVVAVIVVNAYRIKEEYEDEMAPELERRACKKKWNECTRDSDCCDEKGWANQKLQCLQQCDEGGCLEYRQCLFHSGLQRK------------   50   100
U-MTTX-Msn2a       -----MKLQLIVVLLIALVIVSLVDGEML--EKR--CKNRLKKCTSNSECCDKKDRGGRKLRCLTXCDERHCLTYKQCLFYPGLQKVKRLCFTYCRCTI   50   52.0    2e-15
Kappa-AITX-Bcs4a   -----------------------------------GCKGKYEECTRDSDCCDEKNRSGRKLRCLTQCDEGGCLKYRQCLFYGGLQ--------------   50   72.0    3e-20
NvePTx1            MFSARLVLVFAVVLCIQLCNASWLDERAMTQEKR--CNGKYQKCTSNSQCCDQKDYAKRKLRCLTQCDEGGCMKYKQCMFYAGTQK-------------   62   43.5    3e-18
                                                    *: : ::** :*:***:*. . :**:**  ***  *: *:**:*: * *
```

## E) Sea anemone sodium channel inhibitory toxin family. Type I subfamily.

```
                                                                                                    PSI-BLAST
                                                                                        aa    %I     E-value
c22149_g1            -MNRLIILVIAAVFLTLAS----AEDDVDMAKRGVSCLCDSDGPSVRGNTLSGTLWLYPSGCPSGWHNCKAHGPTIGWCCKQ   49   100
Delta-AITX-Axm1a     ---------------------------------GVSCLCDSDGPSVRGNTLSGTLWLYPSGCPSGWHNCKAHGPTIGWCCKQ   49   100     5e-31
Delta-AITX-Ael1b     ---------------------------------GIACLCDSDGPSVRGNTLSGTYWL--AGCPSGWHNCKSSGQLIGACCKQ   47   77.6    3e-20
Delta-AITX-Ael1c     ---------------------------------GVPCLCDSDGPNVRGNTLSGTYWL--AGCPSGWHNCKSSGPLIGACCKQ   47   79.6    4e-32
Delta-AITX-Avd1e1    MMNRLLVFLMLGAAFMLVVSAIDQDANEDINKRGIPCLCDSDGPSVRGNTLSGIIWL--AGCPSGWHNCKKHGPTIGWCCKQ   47   83.7    4e-32
Delta-AITX-Axm1f     ---------------------------------GVPCLCDSDGPSVRGNSLSGIIWL--FGCPSGWHNCRDHGPTIGWCCKK   47   79.6    8e-23
                                                     *:.********.****:***  **   *********:  *  ** ***:
```

**Figure S1) Putative toxins that act on ion channels regulated by K⁺ and Na⁺.** The sequences used in the alignment in A) correspond to Kappa-AITX-Aeq4a (UniProt:P81897) from *Actinia equina*, Kappa-AITX-Avd6a (UniProt:Q9TWG1) from *Anemonia viridis* and U-AITX-Avd11a (UniProt:P0DN05) from *Anemonia sulcata*; in B.1) correspond to KappaPI-SHTX-Shd2a (UniProt:B1B5I8) from *Stichodactyla haddoni*, KappaPI-AITX-Ael3a (UniProt:P86862) from *Anthopleura elegantissima*, KappaPI-AITX-Avd3d (UniProt:Q9TWF8) from *Anemonia sulcata*, KIn-II (UniProt:C1IC52) from *Walterinnesia aegyptia* and Kunitz inhibitor IV (UniProt:Q2ES47) from *Daboia russelii*; in B.2) PI-AITX-Axm2a (UniProt:P81547) from *Anthopleura aff. xanthogrammica*, PI-AITX-Aeq3c (UniProt:P0DMJ2) from *Actinia equina*; in B.3) cVamTi (UniProt:P00991) from *Vipera ammodytes ammodytes* and DrKIn-II (UniProt:H6VC06) from *Daboia russelii*; in C.1) DeltaKappa-AITX-Avd4b (UniProt:P59084) from *Anemonia sulcata*, DeltaKappa-AITX-Avd4a (UniProt:P11494) from *Anemonia sulcata*; in C.2) Kappa-AITX-Ael2a (UniProt:P61541) from *Anthopleura elegantissima*, Pi-AITX-Ael2b (UniProt:P61542) from *Anthopleura elegantissima* and Pi-SHTX-Hcr5d (UniProt:C0HL54) from *Heteractis crispa*; in D) U-MTTX-Msn2a (UniProt:P0DMD7) from *Metridium senile*, Kappa-AITX-Bcs4a (UniProt:C0HJC4) from *Bunodosoma caissarum* and NvePTx1 (UniProt:A7RMN1) from *Nematostella vectensis*; in E) Delta-AITX-Axm1a (UniProt:P01530) from *Anthopleura xanthogrammica*, Delta-AITX-Ael1b (UniProt:P0C1F0) from *Anthopleura elegantissima*, Delta-AITX-Ael1c (UniProt:P0C1F1) from *Anthopleura elegantissima*, Delta-AITX-Avd1e1 (UniProt:P0DL52) from *Anemonia viridis* and Delta-AITX-Axm1f (UniProt:P0C5G1) from *Anthopleura xanthogrammica*. In B.1) and in E) where tryptic peptides were identified, the coverage region is underlined.

## A) Equistatin

```
c17929_g1    -----------------------------------------------------------------------------------------------------MQTIIVNVPGWCGPPSCKADGSFDEVQCCASTGECYCVN-
EQST_ACTEQ   ------------------------SLSKCQQLQASANSGLIGAYVPQCKETGEFEEKQCWGSTGYCWCVDEDGKEILGTKIRGSPDCSRRKAALTLCQ-MMQAIIVNVPGWCGPPSCKADGSFDEVQCCASNGECYCVD-
c33740_g1    ----------------------------------------MKNLHKPRCNPNGSYEEVQCDPRTEQCWCVDQKGRELTGTRTSGYVKCPSAGHSLTSCQKEYQNILRRFLLPHMVPQCTSEGRYEPVQCSEN--HCLCVN-
c32564_g1    MDDKGSEMPGTRAPIGQKIDCLAQGPHLCDTERKINKDKRFGGEYLPQCKQDGTYESIQCSPLTGHCWCVDPYGKEIPGTRTRAPLMCPTVDAQITRCQRQYIDASKNPGLGRLIPQCAEDGNFRIVQCQGPNSTCSCVDR
                                                                                                         .        *.*  :* :  ***      * **:
```

```
                                                                                                                  PSI-BLAST
                                                                                                    --------------------------------
                                                                                  aa    %I    %S    E-value    E-value    E-value
c17929_g1    EKGKELEGTRQ---KGRPTCEKHLS--PCEEARLQAHSNSLRVGMFVPECLDNGSYNPVQCWPSTGYCWCVDEAGVKVPGSDVRFKRPTC   124   N/D   N/D               -----      -----
EQST_ACTEQ   KKGKELEGTRQ---KGRPSCERHLS--PCEEARLQAHSNSLRVGMFVPQCLEDGSYNPVQCWPSTGYCWCVDEGGVKVPGSDVRFKRPTC   199   100   100    4e-83      3e-16      3e-22
c33740_g1    AYGVEIAYSRTSKGEERPVCQPVDIGPTLCQKQLQMYMRADDHRQLEPSCNPDGSFRDIQCYGA--------------------------   162   26.2  46.1   -----                 -----
c32564_g1    ETGEILSGSEITFNLGKPQCKTKGG-LTICQRMHKEAIKHPEMTQYVPQCNENGDF----------------------------------   196   26.0  45.0   -----      -----
               *  :  :.      :* *:       .  :   :   .        *.*  :*.:
```

## B) Two Kunitz

```
c24072_g1            ----------MRVFVCFLLVIYSSIVNCGDDRMRCSKAMITG-NCDEHIERYYYNKDEDSCKRFYYTGCNGNANNFETRDECKEACKGKGFCLFSPATGSCDEKITRYYYDSDSKKCKTFEYSGCGGNKNNFETKDECLTGC
A0A2P6KF00_NEPCL     MIDTKIYHQKKKNEVHDFVRYVLPTVFSKGSAAICYEPMRQGGPCLAHISMFYYDSELKTCQPFIYSGCNGNNNRFNSKEECEKECG-KDVCTMPSAKGLCHEEIWRWYYDYETRECKLFKYSGCHGNENNFETKKECDSKC
PCPI_SABMA           -------------------------------ISVCDLPADRG-QCTAYIPQWFFNKTTEDCEKFVYGGCQGNANRFETKDDCIANC----GCNLPSKVGPCRVSARMWFHNPETEKCEVFIYGGCHGNANRFATETECQEVC
XP_020615985.1       -------MAKRASLVDVFIIIIISGIMVAQGENICNLPSETG-LCKGYFPSYYFDKVARKCKKFIYGGCDGNANNFETLAACQTTCP--ENCQLPKVTGQCRAHILSYYFDKKTGECKKFVYGGCGGNANNFETLAACQETC
KCP_HALAI            -------------------LFVGLTSAKY---HDVCQLPRDPG-PCRAYIPLYYFNSRTCLCEKFVYGGCQGNANRFDTVEDCRRRCGGGDLCSLPRDSGPCEAAIPRWWYNKRTNRCQRFTYGGCEGNANNFKTLDECRFQC
VKT3_BITGA           -----------------PLRPGTEKHTFPFPAEFCNLPADLG-PCKNYTGRFYYDSASNKCEVFIYGGCPGNANNFKTREECRKTC--VEICILPAELGPCDEYTGRFYYDSASNKCEVFIYGGCQGNANNFKTRDECRKTC
                                                  *  .    *  *   :    ::::.     *: * * ** ** *.*.:    *    *      * :.     * *       ::.:   :  .*: *  *.** ** *.*  *     *     *
```

```
                                                                                                     PSI-BLAST
                                                                                              %I     E-value
c24072_g1            R------VHFI----------------------------------------------------       100
A0A2P6KF00_NEPCL     PA-------------------------------------------------------------       45.1    4e-19
PCPI_SABMA           DRYQKPGFCYQPSETGPCKGSFPRYYYDYEDGECKEFIYGGCEGNANNFETKESCENAC--       33.0    7e-23
XP_020615985.1       ---QNPQTCLLPKIIGECQANFPRYYFDKVTGKCKTFIYGGCGGNANNFETKRACNEKCLK       42.0    3e-27
KCP_HALAI            R-----KRSTY----------------------------------------------------       37.5    1e-23
VKT3_BITGA           V-----EICILPAELGPCDEYTGRLLLRLGIKQM-----------------------------       43.8    3e-26
```

## C) Nonclassical Kazal-type elastase inhibitor

```
                                                                                                         PSI-BLAST
                                                                                          aa    %I      E-value
c16642_g1            MKAIFVAFVLVALFACQQTEAKDCSLVCTMQYDPVCGSNGKTYGNACMLRAASCRSNKKIKLVHKGHC    47    100
PI-actitoxin-Avd5a   --------------------KPDCPLICTMQYDPVCGSDGITYGNACMLLGASCRSDTPIELVHKGRC    48    75       3e-21
                                          **.*:***********:* ******** .*****:. *:*****:*
```

## D) Turripeptide LoI9.1

```
                                                                                                          PSI-BLAST
                                                                                          aa     %I       E-value
c41184_g1      MRRFAIFIVLSSLLVVAFAKVGKLKKTQDCNKTCGRLNRPVCGTDGTTYANKCELRNAKCRASKLGKKLLLQYEGACGTPNKMVTVSNF    70     100
TU91_LOPOL     MKVYCLLLVL----LVGLVSQAHGKPTKRCLSVCSAEYEPVCGSDGKTYANKCHLMTEACWSP---TSITLVHEGKC------------    50     41.5     9e-06
               *: :.:::**    :*.:.. .: * *: * ..*.   .****:**.******.* .  * :.   ..: * :** *
```

## E) Metalloprotease Inhibitor

## E.1)TIMP1

```
c28108_g1      --------MALLETAMVFLSVITMTSACSCMPSHPQEHFCQAQFVVRGKVISGPVQVLPDKSLSSNVFGRFLAERVYTFRITKTYKGTAAIKKTGSLKVYGSRGRSFITKIHTPSK
TIMP_DROME     MDLRKHLGLLTLLLVAVFAFYGRPADACSCMPSHPQTHFAQADYVVQLRVLRKSDTIEPGR-------------TTYKVHIKRTYKATSEARR------MLRDGR-----LSTPQD
                       :  *   . **      :.********** **.**::**: :*:   .  : *.:              .*..:*.:***.*:  ::           **     : **..

c28108_g1      LNSCRVDLNVGKVYVIAGYFGRKKLFVSGCNFRMEWKKISKKQRSGFARKYGRSCECNHQKCYEDCGKESQAMACNFDISTNPANECRDRYQYCQLSSDG------RRCSWTKNAL
TIMP_DROME     DAMCGINLDLGKVYIVAGRMPT----LNICSYYKEYTRMTITERHGFSGGYAKATNCTVTPCFGERCFKGRNYADTCKWS--PFGKCETNYSACMPHKVQTVNGVISRCRWRRTQL
                  * ::*::****::** :      :. *.:  *:.::: .:* **:  *.:: :*.   *: :   :.:  *  .  .  *  * .:*. .*. *   .          ** * :.
```

```
                                               PSI-BLAST
                     aa     %I     %S          E-value
c28108_g1      YRKCMNKIP     208    100    100
TIMP_DROME     YRKCMSNP-     184    29.5   56.2        2e-20
               ******.:
```

## E.2)TIMP4

```
TIMP4_HUMAN    MPGSPRPAPSWVLLLRLLALLRPPGLGEACSCAP--AHPQQHICHSALVIR-----AKISSEKVVPASADPAD--TEKMLRYEIKQIKMFKGFEKVKDVQYIYTP----------
c29197_g1_i1   --------MKIIAILVVFCLLINEGFSFSCRCVPGNDHPQDKFCTSDYVLRGLVQSGPVTKTIEIPRPDNPENPWTRSVIVYRVQVQRIYKGENKVKNTTPLKDYPGKVPMPMEVA
                                 . : :* ::.**   *:. :* *.*   ***:::* *   *:*      . ::.    :* . :* :   *..:: *.::   :::** :***:.       :

TIMP4_HUMAN    --FDSSLCGVKLEANSQKQYLLTGQVLSDGKVFIHLCNYIEPWEDLSLVQRESLNHHYHLNCGCQITTCYTVPCTISAPNECLWTDWLLERKLYGYQAHYVCMKHVDGTCSWYRG
c29197_g1_i1   IYTDNNSCAAQLEQG--RDYVLGGYIDGE-DLEIFSCNWIQTWNSLSAGQRIGIRRYYGRNCACRTS-----LCSWMDPLGGMMDYYCGVNNLY-------CAMRNDQATCSWKDI
                  *..  *..:** .   ::*:* *  :  .:  .: *.  **:*:.*:.**   **  .:.::*   **.*:  :        *:    *    :    :    .:**          .*::  :.****

                                           PSI-BLAST
                        aa     %I     %S    E-value
TIMP4_HUMAN    HLPLRKEFVDIVQP----  195   26.2   52.4    6e-13
c29197_g1_i1   GRGYRECIKDATILGFYK  206   100    100
                   *:  :  *  .
```

## E.3)TIMP-LIKE

```
XP_020895880.1   ----MNLTLYLCLAAVCFVTVSKACSCVTRDTSTPEFLCCNDYVLRGTVVKSVVTGNKSRPESQERVFTVKVQEIFKGKEKIKDIAGDTSQVNISTAVQSATCGTNIP
c26215_g1        MFYERLLVFVSFLALLTLIPASYGCKCDVSGRDTPEFLCAFDYVLHGEVINETIIGSNASVKDQERVYTVKVLKVFKGLERIKNMSKDHNTSYVQIYTSTTSCGIYLS
                          *.:    ** :  ::..*  .*.*  .  . .******.  ****:*  *::..:  *.::   :.****:****  ::***  *:**:::  *  .     :.   ..:::**   :.

                                                                                        PSI-BLAST
                                                                                 aa    %I    E-value
XP_020895880.1   --SNKDYLISGHFWN----NKIRNSLCNSWNVEWSSVKQSFKDFTPKLKTCPCGVGCLLPLQALVALAIITCIINYF  155   100
c26215_g1        RLHQQSFVLSGSVDRREGQNRMESESCQDWHSSWEEVTTSYKDIFPMLNTCKCAANTNYVGRFFFLLSMLLTAWNMN  161   30.4    7e-24
                     ::.:::**.  .     *::...  *:.*:  .*..*.  *:**:  *  *:**  *...      :  :.  *:::     *
```

## F) Serpin

```
ILEU_XENTR   --------------------------------MENLSSACTHFSFDLFRKI---NENNATGNVFFSPISISTALAMVLLGARGNTAQQISRILHFDAVK--DLHSNFQTLNAEINKKNVSSYALNLANRLFGEKSFKFLPDFLSSVKKQYNADLGTVDFISA
ILEU_XENLA   --------------------------------MENLSSACTHFCFDLFKKI---NENNSTGNLFFSPISLSTALAMVFLGTKGKTAEQMSKTLHFDAVK--DLHSNFQTLNAEINKKDVSTYALNLANRLFGEKTFNFLPNFLSSVKKQYSADLGTVDFISA
c25417_g1    MKLIIALKIAVVLLLSEAFLCQGDEHDPESKTIKELVHSSNEFAFRLLNFLGTQNEKSKNENLFFSPTSISIALAMVYLGSRGNTATQISDALGWNEYNGEEIHTAFKTLHEAIHNSEDEVFELKLANRIWGHDSLKPLYEFSSAMKEFYGSEITLVDFIKG
c26903_g1    --------------------------------MSAVATAVNQFAVDLHAVLKS-QEKFLGDNLFYSPTSLSIALGMVYSGARGSTAEQIANGFHWKSLEVEKLNESMKSFSDALTSSNTASNELNTANRLFLQQGFEVIQEFTDCCKKFYDAETALVDYKKD
c27265_g1    MYIIVLNTFRSRSLLILTVSLVVASISPSTMAQADIKASSNKFSLKLHQVLRG-QAS-SAENLFYSPSSILVALAMTHLGARGNTAKQMSKSFHLEEIPEKQLNEEFQKFLQSLNQSNARGNEIAMANRLFVQMGFEVSKQFQEESEKFFNAEAALVDYQKN
                                              :  : ..*.. *    :    : .    *:*:** *:  **.*.  *::*.** *::  :  .    .::  ::.:   : ..:      :  ***:: .  ::   :* .  :: :.::   **: .

ILEU_XENTR   AEDARKEINTWVSEQTKGKIPEVLSAGAVNSFTKLVLVNAIYFKGDWAKKFKAEHTKDMPFQL--NKKEQKTVKMMYQMEKLPFNYIPEINCRVLELPYVDYELSMVIVLPDNINDDTTGLQQLEKELSLEKIN---EWTENMMPIDVHVHLPKFKLEDSYK
ILEU_XENLA   LEDARKEINKWVSEQTKGKIPEVLSTGTVDRSTKLVLVNAIYFKGDWAKKFNAEHTTDMPFQL--NKKEQKTVKMMYQKEKLPFNYIPDINCRILELPYVDYELSMIIILPDNINDDTTGLQQLEKELSLEKIH---EWTENMMPTDVHIHLPKFKLEDSYK
c25417_g1    SDEVRKDVNQWVHQQTNGKIRDFLPHGVLNSATKLALINAIYFNGAWKNEFDKEKTFHAPFYASGNREYKMEVNMMTKTSKNKYYFDQEHSCHVLELPYSGNELSMVLVLPEELD----GISKLEKSLDLENLD---RWMTLMVNTTVSVSLPRFKLNQQVD
c26903_g1    FEGARKLINTWVEEKTKDKIKELLPSGSLNSLTRATLVNAIYFKGIWEKQFEKAHTYDSTFFT--SLNKEMKIKMMFQKSKFKFLEARNLDCQLLEMPYTGGDLSMVILLPNEIE----GLSKLEEKLTYDALQGALTSVTKARPLEVEVSIPKFSMTRQFE
c27265_g1    SKGAREEVNKWVEEKTKNKIKDLIPEGMFGPDTRLTLVNAIYFKGSWMSQFDPNATQTGPFQL--TSSQSIEVPMMYQSKKFKYFENQDLKCKMLELPYAGEKMSMIILLPDEVE----GLSKLEESLSYSKLDDGINHLRMAREEEVEVTLPKFKLSEKFS
              . .*: :* ** ::*:.** :.:. * ..  *: .*:*****:* * .:*.   *   .*    . . .  : ** : .*  :    : .*::**:** . .:**:::**::::    *:.:**:.*  . :.                * : :*:*.:  . .
```

Underlined in c27265_g1: RLTLVNAIYFKG

```
                                                                                                                                                              PSI-BLAST
                                                                                                                                                 -------------------------------
                                                                                                                              aa    %I    %I    E-value   E-value   E-value
ILEU_XENTR   LKSQLAGMGMADLFEAGSADLSGMSGS-NDLYLSEVIHKSFVEVNEEGTEAAAAS-AGIAMMCLMRE-EEFNANHPFLFFIRHNATKSILFFGRYSSP-------------------  377   100   88.3   5e-95     2e-91     1e-90
ILEU_XENLA   LKSQLAGMGMVDLFNSGSADLSGMSGS-NNLFLSEVIHKSFVEVNEEGTEAAAAS-AGIAMMCMMRE-EEFNADHPFLFLIRHNATKSILFFGRYSSP-------------------  377   88.3  100    8e-91     2e-88     7e-93
c25417_g1    LKNVLPQLGIQDIFDASKADLSGITES-SGLFVSNVIHKAHVDVNERGTEAAAAT-GVTMMKRSLDLNEVFHADHPFIFFIRHHFSGSILFVGKVMQPSGLTEEESDLPQRTVADEL  409   41.3  38.4             -----     -----
c26903_g1    LKEILQRMGMGDMFDQVESNFTGLIKGPGKLYVSEVFHKAFVDVNEEGTEAAAATAAVMMTRMMVRPPPSFTADHPFLFLIRHCKSDAILFLGRVAKPESKE---------------  387   40.9  39.9   -----               -----
c27265_g1    LKEVLSKMGASDLFNPSKADLTGINKD-GQLFVSEVVHKAFVDVNEEGTEAAAAT-AVGVALMCMPMNPIFFADHPFLFLIRHNESGVILFLGRLANPN-----------------  381   41.0  42.1   -----     -----
             **. *   :*   *:*:   .::::*:   . . *::*:*.**:.*:***.*******: .        :      * *:***:*:***   :   ***.*:   .*
```

In the printed figure, "100" is set in bold where each query sequence is compared with itself (the 100 values in each aa/%I/%S table).

## G) Reversion-inducing cysteine-rich protein with Kazal motifs

```
RECK_HUMAN   MATVRASLRGALLLLLAVAGVAEVAGGLAPGSAGALCCNHSKDNQMCRDVCEQIFSSKSESRLKHLLQRAPDYCPETMVEIWNCMNSSLPGVFKKSDGWVG---LGCCELAIALECRQACKQASSKNDISKVCRKEYENALFSCISRNEMGSVCCSYAGHHTN
RECK_MOUSE   MASVRASPRSALLLLLAAAGVAEVTGGLAPGSAGAVCCNHSKDNQMCRDVCEQIFSSKSESRLKHLLQRAPDYCPETMVEIWSCMNSSLPGVFKKSDGWVG---LGCCELAIGLECRQACKQASSKNDISKVCRKEYENALFSCISRNEMGSVCCSYAGHHTN
c29119_g1    ---MRVLFSTLLLVQTVLS-----------NQPDILCCKQTSGFKQCRSACNKFVSQTDVTSRLHKLKELPNFCPGHLTGLWRCLNGSNPGTRQYVESKLPNKAGVCCLMASTSKCRNDCLNGK-KLALNRSCS---DMHLISCVTRHDASAKCC--HGATKS
               :*.     **:  . :           .... :**:::.. : **..*:::.*... :   * *:. *::**  :. :* *:*.* **. :  :. :      ** :*   :**: * :.. *  :.: *    :  *:**::*:: .: **   *  ..

RECK_HUMAN   CREYCQAIFRTDSSPGPSQIKAVENYCASISPQLIHCVNNYTQSYPMRNPTDSLYCCDRAEDHACQNAC-KRILMSKKTEMEIVDGLIEGCKT--QPLPQDPLWQCFLESSQSVHPGVTVHPP--PSTG---LDGAKLHCCSKANTSTCRELCTKLYSMSWGN
RECK_MOUSE   CREFCQAIFRTDSSPGPSQIKAVENYCASISPQLIHCVNNYTQSYPMRNPTDSLYCCDRAEDHACQNAC-KRILMSKKTEMEIVDGLIEGCKT--QPLPQDPLWQCFLESSQSVHPGVTVHPP--PSTG---LDGAKLHCCSKANTSTCRELCTKLYSMSWGN
c29119_g1    CVKACKLLFASQPIRKTNLLQRVKKNCKSGGRHVFECAKKHEPATKNPNDYKQLQCCSLAKEEPCKEGCRKELLISNRSISESMSRLISVCKNPFQQKLNNAMWNCFLRTHAKPTAEIPVNEEIRPITGTPKMDGAKLQCCPKASTPSCTWLCVKMFTSQWSG
             * : *: :* ::.    .. :: *:: * * . :::.*.:::  :    *   ..* **. *::..*::.* *.:*:*:::  * :. **. **.  *    ::.:*:***.:  .   . :.*:    * **   :*****:**.**.*.:*  **.*::: .*..

RECK_HUMAN   TQSWQEFDRFCEYNPVEVSMLTCLADVREPCQLGCRNLTYCTNFNNRPTELFRSCNAQSDQGAMNDMKLWEKGSIKMPFINIPVLDIKKCQPEMWKAIACSLQIKPCHSKSRGSIICKSDCVEILKKCGDQNKFPEDHTAESICELLSPTDDLKNCIPLDTYL
RECK_MOUSE   TQSWQEFDRICEYNPVEVSMLTCLADVREPCQLGCTNLTYCTNFNNRPTELFRSCTAQSDQGAMSDMKLWEKGSIKMPFISIPVLDIKTCQPEMWKAVACSLQIKPCHSKSRGSIICKSDCVEILKKCGDQNKFPEEHTAESICEFLSPADDLESCIPLDTYL
c29119_g1    SQKFAEFDMKCAYQPSQVKLMNCLADVTKPCQLGCSGLNYCTNFNNRHTELFRSCNAKADSYAKSHMQHWSKGIINVPGMPILVQDIKKCLPDTWKAIACLLQIKPCNVKSHRSTICKSDCIQILQKCGNSSSYSDLKKVEEVCTRLSPTES--NCISIKSYL
             :*.: ***  * *:* :*.::.***** :****** .*.******** *******.*::*. * ..*: *.** *::* : * * ***.* *: ***:** ******: **: * ******::**:***:...:.: :..*.:*  ***::.  .**.:.:**

RECK_HUMAN   RPSTLGNIVEEVTHPCNPNPCPANELCEVNRKGCPSG-DPCLPYFCVQGCKLGEASDFIVRQGTLIQVPSSAGEVGCYKICSCGQSGLLENCMEMHCIDLQKSCIVGGKRKSHGTSFSIDCNVCSCFAGNLVCSTRLCLSEHS----SEDDRR-------TFT
RECK_MOUSE   RPSALGNIIEEVTHPCNPNPCPANELCEVNRKGCPSA-DPCLPYSCVQGCKLGEASDFIVRQGTLIQVPSSAGEVGCYKICSCGQSGLLENCMEMHCIDLQKSCIVGGKRKSHGTSFTIDCNVCSCFAGNLVCSTRLCLSEHS----SDDDRR-------TFT
c29119_g1    KPSKYKDVTDEVTHPCNPNPCKKNKICDINRTCYAAALDGCEAHTCIPGCPLGDASTFLVRMGEYARVPVDSGREGCFRVCHCGGESKLTRCIILNCVYKKDCPIKDKKALKHGEQFMVDCNFCACFAGELMCTKRQCLQPPSQIPVVETEGRPKRNHTPMFT
             :**   :: :***********  *::*::**.  .:. * * .: *: ** **:** *:** *    :** .:*. **:::* ** .. * .*: ::*:  :.. * . *  .** .* :***.*:****:*:*:.* **.  *      : : *         **

RECK_HUMAN   GLPCNCADQFVPVCGQNGRTYPSACIARCVGLQDHQFEFGSCMSKDPCNPNPCQKNQRCIPKPQVCLTTFDKFGCSQYECVPRQLACDQVQ-DPVCDTDHMEHNNLCTLYQRGKSLSYKGPCQPFC-RATEPVCGHNGETYSSVCAAYSDRVAVDYYGDCQAV
RECK_MOUSE   GLPCNCADQFVPVCAQNGRTYPSACIARCVGLQDHQFEFGPCISKNPCNPNLCPKSQRCVPKPQVCLTTFDKFGCSQYECVPRQLTCDQAR-DPVCDTDHMEHSNLCTLYQRGKSLSYRGPCQPFC-RAKEPVCGHNGETYSSVCAAYSDRVAVDYYGPCQAV
c29119_g1    GLPCGCDSKHHPVCASNGQTYPNLCIARCAGFKDSQYQPGSCESIDPCKNNPCAKGQRCLIDRKTCLTPTES--CNQYFCVPESSYCKDMQLEPVCDNEGQQHPNLCSIHFKGKQLAYKGFCKKYCSQPTKPLCGINGETYNSECAAISSRVIVDYEGPCRAI
             ****.* .:. ***..**:***. *****.*::* *:: *.* * :**: * * *.***: . :.***. :.  *.** ***..  *.: : :****.:  :* ***::: :**.*:*:* *: :* :..:*:** *****.* *** *.** *** * *:*:

RECK_HUMAN   GVLSEHSSVAECASVKCPSLLAAGCKPIIPPGACCPLCAGMLRVLFDKEKLDTIAK--VTNKKPITVLEILQKIRMHVSVPQCDVFGYFSIESEIVILIIPVDHYPKALQIEACNKEAEKIESLINSDSPTLASHVPLSALIISQVQVSSS--VPSAGVRARP
RECK_MOUSE   GVLSEYSAVAECAAVKCPSLSAIGCKPIIPPGACCPLCAGMLRVLFDKEKLDTIAK--VTSKKPITVVEILQKVRMHVSVPQCDVFGYLSIESEIVILIIPVDHYPKALQIEACNKEAEKIESLINSDSPTLASHVPLSALIISQVQVSSS--LPSSAVVGRP
c29119_g1    GHQVG-SGEGRCKDVKCPALVPANCVGIYPPGACCPHCAAQIRILVSLTQLSINNEGLKDHSKPITLEHLLVALRRHVTTTECDLFGYQSIEGDLVVLIKAITNKPTALQIEACSLEAQRIEALINTESPSFNSHVILSTLKAADTQTPSLSLRQTQGSSRNH
             *     *. ..*  ****:* . .*  * ******* **. :*:*..  :*.   :     .****: .:*  :* **:..:**:*** ***.::*:** .: : *.*******. **::**:***::**:: *** **:*  ::.*..*     : .   .

                                     PSI-BLAST
                        aa    %I     E-value
RECK_HUMAN   SCHSLLLPLSLGLALHLLWTYN-   949   40.2    0.0
RECK_MOUSE   LFHSLLLLLSLGLTVHLLWTRP-   949   39.6    0.0
c29119_g1    LYIPHLIICLLQTFIILKWSLRS   960   100
               . *:    *    : * *:
```

## H) A.superbus venom factor 1

```
c31832_g1    MGPNVKLKCLLIFLAQGLSLVLCKSPGYVILAPNVFHVGLEETIAVTVTGVAKPVKVKLFLQEFPNRRSNFSHVEGFFRSDKSGLLKIKVNPRDVPSHDTEYNQYLYLVASCDDPQLVFHKEVKILLNYRD
c32153_g1    ----MKMLLSIILFAAAS---CASAQRYFISAPNVFHVGTPEKVSVTVFGINSPITVRLFLQDYPHRRKTFSPVQAVFQPDVPQFLKVRVDPKDIPDQQAVDKQYVYLVAKSDDQTLRFHKETKILLSFKD
VCO31_AUSSU  ---MEGMALYLVAALLIGFPGSSHGALYTLITPGVLRTDTEEQILVEAHGDSVPKQAVISIHDFPRRQKTLFQTRVDMNPAG-GMLVTPTIKIPAKELNKESRQNQYVVVKVSG--LPLELEKVVLLSYQS
                 :   ::            . .  * : :*.*::..  * : * . *   *  . : ::::*.*:..:  ..  :..    :*   .      . :   .*  *:*.. ..  * :. *  :**.::.

c31832_g1    KIVLLQTDKPIYTPKQTVKFRVIPLDFNLRPSKDKVNIFIKNPQGILAQRWANVSSETGFITKALHLGDHSLIGNWTIVITYGYLNSQNTTVKFEVKEYVLPRFSLRIIGPS--YILPSAKRIDIAIVAKY
c32153_g1    GVILIQTDKPIYTPKQRVKIRVIPLGFDLMPTTKKITVVVKNPQDIRVQQWKNLDTKTGFISKVLDLGDFVLNGNWTVQAFYGHLFIHNTSIKFEVREYVLPTFSVKISGPS--VILPTDKAISVGITSRY
VCO31_AUSSU  GFVFIQTDKGIYTPGSPVRYRVFSMDYNMHRMDKTVIVEFQTPEGVVVS--SNPVNPSSVLIRPYNLPELVSFGTWKAVAKYEHSPEESYTAYFDVREYVLPSFEVRLQPSDKFLYIDGNKNFHVSITARY
              .:::**** **** . *: **:.:.:::    ..: : .:.*:.: ..    *  . :..: :  .* :     *.*.     * :    .. :  *:*:***** *.:::  ..      :    * : :.*.::*

c31832_g1    TYGKPVIGSVITRLTITGKGEIE-VKFFVNSSKLNDGKVQLKIDTSLLKNHALRPWFPNGRRLRIDADVVEQATGTRESAVDSSIYFVKTPFHLNYENTAMYFKPAMPYAIKIDVKYPNKRPASHLPVQIS
c32153_g1    THLNSVQGTLAVRIKIVGMKQEP-ILISSFRMRLIDGFASITIKQGHIRDLPGNIWFPEGSRLQVEADVTESSTGTTEHAIENSIFFSSSKYRIKFYKTARYFKPGLPFSVKAIVSYPDDKPVPNIDVRIS
VCO31_AUSSU  LYGKKVEGVAFVLFGVKIDDAKKSIPDSLTRIPIIDGDGEAILKRDTLRSRFQNLNELVGHTLYASVTVMTESGSDMVVTEQSGIHIVTSPYQIYFTKTPKYFKPGMPYELTVYVTNPDGSPAANVPVVSE
              : : * *    . : :          :         : **  .  :. . ::.    .      *   *   .. *   .: .     : :..*.: .: ::: : :*. ****.:*: :.   *. *:   *..:: *   .

c31832_g1    AKGTSDAGKPVNVMQKLNDRIRKYMKGNTDGQGRVEFILDVPRSVKDIEIEVETKDARLG-KANAAVKFKAKAYNSP--SKSYLHIRPKNPKGRVGAVSMWDVFQN------GNISSLTFMVLSRGRILLQ
c32153_g1    AKAIKSDGKETEVGRHVGDIKDEANRDVTGHDGQAEFVIDIPRDVTTLKVKVQTAEIGLPPKQNAIKELITHVYTSI--KNEYLMLRQTRPVS-VGKTVFCDAFLSS-----GKVNKLSYMVVSRGQIVLH
VCO31_AUSSU  AIRSE---------------------GTTLSDGTAKLILNTPLNTQSLPITVRTNHRDLPSERQATKSMTATAYQTQGGSGNYLHVAITSAEIKAGDNLPVNFNVRGNANSLNQIKYFTYLILTKG-KIFK
             *    .                    . *  :* .::::: * ..  : : *.* .  *  : :*  .: : .* :   . .** :  . .   .*      :             .::. ::::::::*  :::

c31832_g1    VLTRPKDGVNVVSTITFTVTKEMSPACRVLAFYVTKDNEVVADSDVMEVEERFPNKVRFT-SPGKQVKKERPGMDYVIEVESTPGSRVGLLGIDESVYLLRNKNRLTKKKVFKDVKNLDLGCGVGPGKNNQ
c32153_g1    NVIVKK--FGVTATISFQVTSMMSPSARLIAYYISPTGEVIADSILLEVPDSLPNQIQIK-DNEHEAVTSFPALEYTVNVLGRKGTRVGILGVDQSVYLLRNDNRLTPKRVFNELEDLDLGCGVGSGKNTE
VCO31_AUSSU  VGRQPKGEGQNLVTMNLRITPDLIPAFRFVAYYQVGNNEIVADSVWVDVKDTCMGTLVVKGASSRDNRIQKPGAAMKIKLEGDPGARVGLVAVDKAVYVLNDKYKISQAKIWDTIEKSDFGCTAGGGQNNL
                 *        *:.: :*  : *: *.:*:*    .*::***  ::* :    . : ..     ::    . *.    ::: .  *:***::.:*::**:*.:. :::  :::. ::. *:** .* *:*.
```

```
c31832_g1     DVFKNSGLMVITNAMESMKERANFGCSAAETKKRRKRSLSYQEKN------------SCCLNGKLFS--RASCVMRARFMAMNGVS----LECREVFLNCCRIAWNEKAPVVDKRQRQFFEQNNGLFFGGE
c32153_g1     GVFKNAGIAVIAQSLT-VSERQDFKCGDHLARKKRSVIQNSDPQT------------FCCRLGNTTDDENRTCIRRVSDLDVPEYTGQELKECEKYFLKCCVRRFGAQDMGFLARNAGFDDFAG----PSD
VCO31_AUSSU   GVFEDAGLALTTSTNLNTKQRSVATCPQPTNRRRRSSVLLLDSKANKAAQFQDQNLRKCCEDGMHENPMGYTCEKRAKYIQE-------GDACKAAFLECCRYIKGIRDENQRESELFLARSDFEDELFGE
              .**:::*: : :.:    .:*    *     :::*.     : :              **  *    .    :*  *.  :            *.  **:**    . :       :  :          .:

c31832_g1     DNDIQSEQIRKYFPETWIFDEEIIGPD---------------GRMMLPVTLPDTITTWVLQAISISNTSGFAMTSPSRITTFKKFFVTLSLPYSVQRGEQLSVVATIFNYGEEMSQFDVSLFGSREFCSTE
c32153_g1     DELIEMTQVRSYFPETWIYEEKVIGDEVNHGDCEKYLKRRHCGRAMLEATLPDTITTWVVQAVAVSNTSGFGIAKPLHIKTFKPFFMRFTMPYSAHLGEQISIVATVYNYNKEETSIKFYLFGDEGFCSAA
VCO31_AUSSU   DNIIS----RSDFPESWLWLTEDLKEPPNS---------QGISSKTLSFYLRDSITTWEVLAVSIAPTKGICVAEPYEITVMKDFFIDLRVPYSVVKNEQVEIRAVLYNYADEDIYVRVELLYNPAFCSAS
              *: *.    *. ***:*::  : :                  .   *   * *:**** : *:::: *.*: ::.* .*..:* **: : :***.  .**:.: *.::** .*    . . *: .  ***:

c31832_g1     RPGD---KTKPITLSVKANDAKSVTFPIVPIQLGKIPIRVSAIRNMIGIGAVGSDIVQKDLLVVPEGVQRRFVHSLVLDPQGVLRDEKQTKQNVITP----APTLKSARIVQDGQ--QINTVMLTVPSRAI
c32153_g1     PAQERVLLTNKEGITVKAGEALSLPIAIVPIKYGLVPIKVAVVDSRT----FQIDTVEKKLLVVPEGVEKRKTQSYVLDPQGVLKDRKQDGEGVQDLNKAKNATFPSQRTFDRSKNTQYDNILVEVPRESI
VCO31_AUSSU   TEGQ----RYRVQVPVKALSSWAVPFVIVPLEQGLHDVEVKASVRGE----LASDGVRKKLKVVPEGERKNIVTVIELDPSVKGVDGTQEQT-----------------------------VIANKLDDKVP
                 :         :.*** .: ::.: ***:: *   :.* .          .  * *.*.* ***** .:. .    ***.    * .*                                   :   :  .

c31832_g1     PGSIKASIYLTGNLIGPVVTNLIKGG-LDNILRMPMGCGEQNMIFLAPNVYVLEYLTNT---RQVTAEVEARAYRFIQQGYQQELGFRRTDKSFSAFGETRPGSTWLTAFVVKVFCAARKFDGVAIDDHLV
c32153_g1     PESVGAMIYFTGNMIGPVVSNLINGG-LEKLLRMPDGCGEQTMIYMSPNVYVLQYLSNT---RQVTASVEAKAYKFIQSGYQRELNYRRTDHSFSAWGQSRPGSTWLTAFVLKVFCQASKFDGVQIDQDLL
VCO31_AUSSU   ETEIETKISVLGDPVAQIIENSIDGSKLNHLIITPSGCGEQNMITMTPSVIATYYLDATGQWENLGVDRRTEAVKQIMKGYAQQMVYKKADHSYAAFPN-RASSSWLTAYVVKVFAMAAKIV-KDIKHEII
                .: : * . *: :. :: * *.*. *::::  * *****.** ::*.* .  **  *   .:: .. .:.* : * .** ::: ::::*:*::*: : *..*:****:*:***. * *:    *...::

c31832_g1     CDSMKWLISN-QRLDGAFPEVHDLVHKSLLGG---VHGDVAMTAFVLTALLECKCSS----ENSEVSIRKAVLYLETELASLN-KAHVLALTTYSLALAKSTASSGANQRLFQMSSFDKDQNTRHWDVGGP
c32153_g1     CRSVDWLVQN-QRADGAFPEVRAVIHKEMVGGAYQSHSDIAMTAFVVTALLECKCNN----MLKTAAITRAITFLENNLATINDKPYHLALTTYALAMANSAARVRANDRLVQLALYNSVTSSRHWNAGGH
VCO31_AUSSU   CGGVKWLILNRQQPDGVFKENAPVIHGEMLGGTKGAEPEVSLTAFILTALLESRSVCNEHINILDSSINKAIDYLLKKYEKLQ-RPYTTALTAYALAAAERLN----DDRVLMAASTGRDRWEEHN---AR
              * .:.**: * *: **.* *   ::* .::**    . ::::***::*****.:.              :* :*: :* .:  .:: :.:  ***:*:** *:       ::*:.  :  .     .*    .

c31832_g1     ALNVETAGYALLAQLHLGRLKHGGPIVKWLTEQRNAEGGFVSTQDTCVALQALAKYSEKTAGAQLDLRVSITPER-DAVWRRTFHVEKRNALIRRKVDITPFLGGELFIDSSGTGVGQMQIEIVHNVPSSK
c32153_g1     ALNIETAAYALLAQVKMNRLKYAGPIVIWLTEQKNSGGGFQSTTDTCVALQALAVYSEQTAGSKLDLKVTIRSELGRVKLFRQLRINKNNALLQQSVDIFDYLGGEIFVDTVGTGVAQMQVDVRYNVPSTR
VCO31_AUSSU   THNIEGTSYALLALLKMKKFAEAGPVVKWLIDQKYYGGTYGQTQATVMVFQALAEYEIQIP-THKDLNLDISINLPEREVPLRYSINYGNALVARTAETK--LNEDFTVSASGDGKATMTILTVYNAQLRE
              : *:* :.***** ::: ::  .**:* ** :*:   * : .*  * :.:**** *. : . :: **.: *  :              ::  ***: :..:    *. :: :.: * * . * :   :*.   .

c31832_g1     NETCLFDLRVSVREEFDDNKVSDDGLFIVGRSARRTKRGLRSRRKKKTNKRCKRRKGKKSRHCRKPKSKKGSKKSIKKKKPRKSKKRRILKKKNEKKKEKEQKTQARAPDSLYVEVCTRYRKPGKSGMTIM
c32153_g1     KDRCDFMLSVTVSEVGSGRTLSR--LPLNDAPALAPTRKPRGKKNRKKNK--KNRKGK-NRKNKNKKNRKGKKD--KKKKP----KRKPTKKPKKKKKPNQQSKKGKP---LIIRVCTQYKKPGKTGMSII
VCO31_AUSSU   DANVCNKFHLDVSVEN-------------------------------------------AQLNSKQAKGAKDT-------------------------------------LRLKICTRYLGEVDSTMTII
              .      : : *                                               : *. ** *.                                          * :.:**:*    .: *:*:

c31832_g1     DIGIFTGFEPDEKSLIELKKNVQPTVDRFEISD-----RSIVLYISEVFSSKEFCAHFKVNRLFGIGAVQPVPIKVYDYYEPGDSCTKFYAPDLKSELRLGICKGEQCKCTQDGCSSCNIE-SLDVDLMLK
c32153_g1     DVGLLTGYQVDVNSVKEMMRKNNATIANYELSQ-----RSVVFYLDEIDSASQICVAFKVAPIIAIDAVYPVPVKVYNYYEPDEACTKFYSSE-KTSLIMGTCEGKSCKCTQDKCTAPNPS-IRNLDKLVR
VCO31_AUSSU   DVSMLTGFLADAEDLTRLSKGVDRYISKFEIDNNMVQKGTVVIYLDKVSHSEVECLHFKIHKHFEVGFIQPGSVKVYSYYNLDEQCTKFYHPDKGTGLLNKICHGNICRCAEESCSLLNQQKKIDLQLRIQ
              *:.::**:  * :.: .: :  :  : .:*:.:      ::*:*:.::  :.  *   **:   : :. : * .:***.**: .: *****  .:  : *    *.*: *:*::: *:  * .   :::  ::

c31832_g1     KACR-DHEFVFKGEIALVDEDSKGSQSWLNYFITVQKIFKQLDKSKLNVGDHIEYKKRAGCRCPELSERGEYLFMGQE------RNGKQYVLDRKTFVVPWRGKTRDEQSMIVAFRARMNRGHTCPLQ---
c32153_g1     QVCG-MYNYAIKVEVLLVDEQG----AWLTYDVVIKKVIKQG-KQKLERDQVIELRKRGMCNSPRLKKGDILLVMGRD------KSDK-FILDNSAFVKKWR-RGRDAR-QLEALAKRVVDGECS------
VCO31_AUSSU   KACAPNVDYVYKTKLLQIEEKD----GNDIYVMDVLEVIKGGTDRNPQAKARQYVSQRKCQEALNLKLNNDYLIWGLSSDLWPRKNDISYLITKNTWIERWPNEDECQDEEFQNLCDDFAQLSNTLTIFGC
              :.*    ::. * ::  ::*..    .   * : : :::*   . : :        :*   .. .*.  .  *. * .      :..  ::: ..:::  *  . .      :  :   .
```

| | | | | | PSI-BLAST | |
|---|---|---|---|---|---|---|
| | | aa | %I | %S | E-value | E-value |
| **c31832_g1** | -- | 1726 | 28.2 | 59.0 | | ------ |
| **c32153_g1** | -- | 1723 | 27.5 | 57.7 | ------ | |
| VCO31_AUSSU | PT | 1630 | **100** | **100** | 3e-157 | 1e-152 |

**Figure S2) Protease Inhibitors.** The sequences used in the alignment in A) correspond to Equistatin (UniProt:P81439, EQST_ACTEQ) from *Actinia equina*; in B) correspond to BPTI/Kunitz domain-containing protein (UniProt: A0A2P6KF00, A0A2P6KF00_NEPCL) from *Nephila clavipes*, Carboxypeptidase inhibitor SmCI (Uniprot:P84877, PCPI_SABMA) from *Sabellastarte magnifica*, Carboxypeptidase inhibitor SmCI-like (RefSeq: XP_020615985.1) from *Orbicella faveolata*, BPTI/Kunitz domain-containing protein (Uniprot: P86733, KCP_HALAI) from *Haliotis asinine* and Kunitz-type serine protease inhibitor bitisilin-3 (Uniprot: Q6T269, VKT3_BITGA) from *Bitis gabonica*; in C) correspond to PI-actitoxin-Avd5a (Uniprot: P16895, IELA_ANESU) from *Anemonia sulcata*; in D) correspond to Turripeptide Lol9.1 (Uniprot: P0DKM7, TU91_LOPOL) from *Lophiotoma olangoensis*; in E.1) correspond to Tissue inhibitor of metalloproteinase (Uniprot: Q9VH14, TIMP_DROME) from *Drosophila melanogaster*, E.2) Metalloproteinase inhibitor 4 (Uniprot: Q99727, TIMP4_HUMAN), E.3) Uncharacterized protein (RefSeq: XP_020895880.1) from *Exaiptasia pallida*; in F) correspond to Leukocyte elastase inhibitor (Uniprot: Q5I0S8, ILEU_XENTR) from *Xenopus tropicalis* and Leukocyte

elastase inhibitor (Uniprot:Q52L45, ILEU_XENLA) from *Xenopus laevis*; in G) correspond to Reversion-inducing cysteine-rich protein with Kazal motifs from *Homo sapiens* (Uniprot: O95980, RECK_HUMAN) and *Mus musculus* (Uniprot: Q9Z0J1, RECK_MOUSE) and H) correspond to A.superbus venom factor 1 (Uniprot: Q0ZZJ6, VCO31_AUSSU) from *Austrelaps superbus*. In B) the percentage of identity was calculated considering only the region of the sequence that contemplates the two Kunitz domains. In F) where tryptic peptides were identified, the coverage region is underlined.

**A) Angiotensin-converting enzyme. Peptidase M2 family**

```
ACE_THETS     MNLINFSYLNLLFGAGLFSVLESATILNTESDAKKWLTTYNDEAGKYIYDATEAEWNYNTNLTDHNLGISIKKSNDLATFTEQKAIEANKKFVWKNFTDPLLKREFSKITDIGTASLSDE
c22664_g1     ---MMEVYQSLLFKIYLLVLLSSLTPADEEVNAKAFLALYNERAAKETRDQETAMWNHATNITEENMRLRTAAVLKYSSFGDEARRNASRFDLSKLTED--TKRQISMIMES--ATLKDT
                :   * .***   *: :*.* *  : * :** :*: **:.*.*   *   * **: **:*:.*: :       . ::* ::   :*.:  : *   *   **::* * :   *:*.*

ACE_THETS     DFQK-MSGLNSDLTKIYSTAKVCNKPNDPSGKCYPLDPDLSDIISKSNDLEELTWAWKGWRDASGKHMPDKYDEFVQLLNKAANINGYEDNGDYWRSWYESPTFRKDCEDLWQEIKPFYE
c22664_g1     TKRKELAKLIGHMEEIYSSAKVSHP---LIGTNISLVPDLVKIMAESRNNKKLVTAWREWRRVTGPKLKPLYKKFVELSNEGARDNNWGDTGDWWRSWYKPANIANEVEKLWIDLKPLYM
               :* :: * ..: :***:***.:      *.  .* *** .*:::*.: ::*. **: ** .:* ::   *.:**:* *:.*. *.: *.**:*****:...: :: *.** ::**:*

ACE_THETS     QLHAYVRRKLQKKYPQIAFPKEGHIPAHLLGNMWAQSWENIEYLLRPAPDLPSMDITEELVKQNYTALKLFQLSDTFFKSLGLIQMPQPFWEKSMIEKPADRDVVCHASAWDFYNRK---
c22664_g1     ELHAYVRHRLQKQYP-VVLDKE-PIPAQLLGNMWSQSWANIFDLLVPYPGKSSLDLTESLKKKNYTARKIVELAESFFVSIGLEKLPATFYNDSMIERPKGREVVCHASAWDFYMKNSKN
              :******::***:** :.: **  ***:******:*** **  ** * *. .*:*:**.* *:**** *:.:*:::** *:** ::* .*::.****:* .*:*********** ::

ACE_THETS     --DFRIKQCTVVDMHWFMTTHHEMGHIEYYLHYKDQPISFRSGANPGFHEAIADIASLSVATPEYMQSVSLLPNFTDDPNGDLNFLMNQALTKVAFLPFGYLIDQWRWDVFSGDTPRPKY
c22664_g1     LPDVRIKQCISITHQDMVTTHHELGHIYYFLGYWNQPCVYRNGANPAFHEALGDTLSLSVDTPRHLVEIGLLDSYAEDEESGLNALMKTALNTIAFLPFGYLMDQWRWKVFKGEIKESEY
                *.*****  :  : ::*****:*** *:* * :**  :*.****.****:.*  **** **.:: .:.** .:::* :..** **: **..:*******:*****.**.*:  ..:*

ACE_THETS     NSKWWHNRCKYQGVYPPVIRSEQDFDAGSKFHVPNNTPYIRYFVAHIIQFQFHEALCKAANNSRPLHRCNIANSKEAGKKLAELMKSGSSIPWPKVLENLTGSEKMSAKSLMAYYKPLID
c22664_g1     NKRWWELRLKYQGIKPPVGRDESFFDPGAKYHIPANTPYLRYFVSNVLQFQFHKAACQAAEVKGPLYQCSIYKSKAAGAKISAMLEMGRSKPWPKALETMTGSSQIDVGPLKEYFRPLRS
              *.:**. * ****: *** *.*. **.*:*:*:* ****:****:::*****:* *:**: . **::*.* :** ** *:: ::: * * ****.**.:***.::.. .*  *::** .

ACE_THETS     WPEKR---------------------------------KPRAENWMGGKMSSWIV----------------------
c22664_g1     WLVKERCDNKYSIGWPGESGPSYDPCNPPSFVPTTDAPNPRSKAHVTDIYGIMIIVASMFAHLTSQFAVQGTIYGQP
              *  *.                                 :**::  :  .  .  *:
```

| | aa | %I | PSI-BLAST E-value |
|---|---|---|---|
| ACE_THETS | 593 | 40.4 | |
| c22664_g1 | 641 | **100** | **2e-178** |

**B) Nematocyst expressed protein 6. Peptidase M12A family**

```
c25970_g1     --MAQYLIHVILVASFIG----AINSGVIDKKIENNFEGSHEN-DDAARVEEK----AGLFEGDIDMDES-MKEALIGGKISQRDAILNPIYRWPNAKLYYKLHSSISYY--AMYQIHLA
c28588_g1     --MKYFITLLVLMQVFNGLSVPAKKRSNLQKLLEIIQLRSNDNPEEMAYMPEENIVSSNMFEGDIHLDRS-TSDIFNQLLSPSREALVNPVTRWPNARIPYVFG-AVSTL--VKNAFHAA
c7767_g1      MIHGLFASLLLAYFTHTAHLLEPDNTASFDIILQANLELHASN-FSSPPLPKD------VFEGDIALTEEQRRNIDIFGNALGKAVDRNKDIRWPGGVIPYEIDCSLQKMPNAKDAIKKA
VMP_NEMVE     ----------MKGFIFAGVLVSALICLAEGKPFDN---------------------LELVEDDMLMTKEQKEAYLAHQNGRVRRAALRDRYLWPQGKIPYTFSDDIDQAG--RELAERA
                         :     . .     .        ::                        :.*.*: : ..               :  .   .     ** . : * :    :.          . *

c25970_g1     MDHIEAETCVRFEYHHGQPDKNYVVFFKGRGCYSRIGRNPIGRSQKISIGRGCT--RVGTIVHEIMHALGFFHEQSRQDRDKFVKIMWNNIKRKHSHNFRKYPRLLGTDLGKPYDYLSVM
c28588_g1     VKEYEKHTCIKIVPRTS--ERDYIYIVSEGGCWSSVGRS--GYRQKLSIGRNCE--RKGIVMHELMHTLGFFHEQSRLDRDDFITVHWENIDPKRRFNFQKYDHGKADTLGFGYDYESIM
c7767_g1      MKEWESKTCLRFVPRTK--QKDYLWFFRQKGCWCNVGRI--GGKTSLSVGNGCE--FQHVMVHEIGHAVGFWHEQSRPDRDSYVRIVKANILPGFESAFAKYSRDKIDSLGLPYDYESIM
VMP_NEMVE     MNHWMSRTCLRFSPRRR--EHAYIEFQYDGRCRARVGYTG-EARQKVSIGSALDPCPLGSVIHELGHGIGFFHEHSRPDRDEYVNINVNNMREGAESNFRKDNGYFVDSRGQDYDYGSIM
              :..    .**:::  :     :: *: :      *  . :*          .:*:*          ::**: * :**:**:** ***.:: :    *:          * *          *  *** *:*

c25970_g1     HYSKYAFS-RNRKPTIVPRKKNAVIGQRYGLSYYDAYQINKLY-NCHT------------------------LCVDKIHPFICEYYKNRGYCHQTHRGKDVDSLCKRTCTHCGHIQTYK-
c28588_g1     HYPMYSFT-SNGRATIVPKDPKAKIGQRERFSDIDIGQLNKLY-KCPKKSATTPATTLAPTTTPAPTTKPKKPCVDKLS--ACPYYAWFGYCNYYFEFMTTN--CRKSCDRC--------
c7767_g1      HYPFTAFS-RNGKPTLKALRPLNGKKPYKRLSSLDAKQANIMYGNCKRAPEPLKRRGKRGVVEKNEFKSRQKRAICRDSDRYCQRRKRLNQCYTYRNYMSRY--CRATCGWCRNTNTANS
VMP_NEMVE     HYSKYQGNNAFNAVVMEPIQRGAEIGQRDGLSAGDIRQTNLMY-KC----------------------------NAQGDSELQPVNDEDEDKDGGDSKKKPDPKGPKPGEIEE-------
              **.    .      .: .           :*  *  * * :* :*                            :                                .
```

| | | aa | %I | %S | E-value | PSI-BLAST E-value | E-value |
|---|---|---|---|---|---|---|---|
| c25970_g1 | ---- | 245 | 29.1 | 52.5 | | ----- | ----- |
| c28588_g1 | ---- | 253 | 27.2 | 48.6 | ----- | | ----- |
| c7767_g1 | GCRD | 282 | 26.1 | 50.6 | ----- | ----- | |
| VMP_NEMVE | ---- | 267 | **100** | **100** | 2e-34 | 8e-31 | 1e-31 |

## C) Venom metalloproteinase (M12B) family

```
VM3E2_ECHOC   --MMQVLLVTICLAVFPYQGSSIILESGNVNDYEIVYPQKVTALPIEAILQPE--QKYEDAMQYEFEVNGEPVVLHLEKNKNLFTKDYSETHYSPDGREITT-KPLIED
VM3_BUNMU     --MIQALLVTICLAVFPYQGSSIILESGNVNDYEVVYPQKVPLLPKGGVQNPQPKTKYEDTVQYEFEVNGEPVVLHLERNKGLFSEDYTEAHYAPDGREITT-RPPVQD
c29916_g1     MFSLTLCVVLLWCCVLTTNGETHRPLSEYIKYYETLS-YSTSALTKQHDRHRRSLNPKSSPILLNFEAHGRKFKIRLRRHASIFSDDFVADNTEFDTSKVVEGEVLGHK
                :   :* :  .*:. :*.:     *  :: ** :     ... *.      : .      ...:  :**.:*. . ::*.:: .:*:.*:    :    *  ::.   .   ..

VM3E2_ECHOC   HCYYHGRIQN---DAHSTASISACNGLKGHFKLQGETYLIEPLKIPDSEAHAVYKYENIEKEDEALKMCGVKHTNWESDEPIKEASQLFATSEQHRFR----------E
VM3_BUNMU     HCYYHGYIQN---EADSSAAISACDGLKGHFKHQGETYFIEPLKLSDSEAHAIYKDENVEEENETPKICGLTETTWESDEPIRNASLLIYTPEQNRYLKV--------K
c29916_g1     KSLVHGFISDGIFEGKIHAGNDEFHVEPSHKYFQGKQLFHSVIFKSEDVQYPHEHGSGCGMRDKTKRWMEKVHAS-EIKEPKMQFQHSNYEPVLHRYRRAASQDPDPDK
              :.  ** *.:   :..   *. .   .   .*    **:   : . :  .:.  :.   :  ..      .::: :      .:. * .** :  .      .   :*:                :

VM3E2_ECHOC   RYIEFFIVVDQRMYNKHNN-DSAAIRTWIFEMLNTVNEIYLPWNIH-------VPLVGLEFWTQGDLINVVSSADKTLDSFGEWRRRDLLNRKAHDN----AHLITAMH
VM3_BUNMU     KYIEFYVAVDNRMYRHYKR-NKPIIKRRVYELVNILNTILRRLNFH-------IALIGLEIWSKRDKINVQSDVKATLKSFGKWREKKLLPRKRNDN----AQLLTRID
c29916_g1     KSCRLYMQADHLFTEHVGQGNKERALLKMTEHVQAINTIYPGIKFFGKAESDKIEFVIARMLANGSAEQSEASNPFSSANIGVEKLLELNSQQGRNKGYCLGYIFTYRD
              :  .::: .*: : .:  . :.       : * :: :* *     ::.        : ::  .: :: .   :   :.    :   .:*   :   .*    :: .::     . ::*  .

VM3E2_ECHOC   FDAQTLGLAYTGSMCHPKYSTGVFQDSSEINIFVAI-------------------TLAHELGHNLGISHDVP-SCT-CQTKA----CIMSPYLSDQPTKLFSNCSEIQK
VM3_BUNMU     FNGNTVGLAALGSLCSVKYSVAVIQDYSKRTSMVAS-------------------TMAHEMGHNLGINHDRA-SCTSCGSNK----CIMATKRT-KPASRFSSCSVREK
c29916_g1     FNDGVLGLAWVGSPTDAGGICEKERTYSDGTKILNTGVVTFINYGKAVPQRVSEITFAHEVGHNFGSPHDTSGECTPGGSDGNYIMFARATEGSKPNNRKFSPCSIKKA
              *:  .:***  **              :  *. . ::                   *:***:***:*  ** .  .**    :.          :.  :      ** **   :

VM3E2_ECHOC   PLRTDIISPPVCGNGLLEREEECDCGSPENCR---DPCCDAAS----CKLHSWVECES--GECCD--QCRFKRAG-TLCRPARDDCDMAESCSGHSADCPIDGFHANGQ
VM3_BUNMU     PLITDIVAPAICGNYFVEVGEECDCGSPRDCR---SACCNAAT----CKLKHEAQCDS--GECCG--KCKFKKVG-AKCRAAKDDCDLPERCTGRSAECPTDIFRRNGL
c29916_g1     KCCFEPSGAAICGNAVVEKDEQCDCGFEEDATCKSDKCCNGATEGGGCTLKPDKHCSPSQGLCCNGDTCKAQPGNSSFLCQNETECRNISYCNGVNASCPLAPEKPHHT
                :   ...:*** .:*  *:****  .:.     . **:.*:     *.*:    .*..  * **.    *: :   . :      . :*     . *.* .* **     : :

VM3E2_ECHOC   PCSHNLGYCYNGKCPLTLYQCRAFLGKDVVGVQESCFQYNRLGN---------TYAYCRKENGRKIPCAPKD---------EKCGRLYCS-------------------
VM3_BUNMU     PCQNKQGYCYNGKCPTLTNQCIALMGPNVKVSRDSCFTLNQRGK---------GCGYCRMQNGAKIPCAAKD---------IKCGKLFCK-------------------
c29916_g1     PCMEGRKLCENGSCTASVCKGKGYAECQCSEEEKMCDLCCDISTDPKKPDCKPTSKISGMTEMKQLPSAACDNFNGYCDVFLKCRKVDADGPLNRLRKKFFSKEALLGY
              ** .     * **.*.     :   .      :     .. *        ..              .    :  ::*.*. *             ** :: ..
```

```
                                                                                                          aa    %I     %S    PSI-BLAST
                                                                                                                             E-value
VM3E2_ECHOC   -------------------YKSFGDYISCLPCYRANEEDKGMVDEGTKCGEGKVCSNGYCVDLNVAY------------------------------   419   19.1   43.7    1e-12
VM3_BUNMU     -------------------KRNSG---VCNCLILPDDPNYGMVETGTKCGDGMVCSDRKCVKLQTVY------------------------------   426   22.9   43.2    5e-21
c29916_g1     LAWIKKYWWAVVLMGVALILLMAAFIKLCSVHTPSSNPNKKPARQLTLRRQQHLQEERNRRARQARDTEGYPGPSYGDEPPPYPGRGMEMQGGRYHR  556   100    100
                                   .    *     ..: :    .    *    :   :  .:       :.
```

## D) Endothelin-converting enzyme 2. Peptidase M13 family

```
c30734_g1_i2  ------------------MSNELPSETTTHVPLLKYEDQDSLVG------FS-GSREGMAD-SDSLEGHIITVVKPSS-------DNYLVRIIKKPTFWL---AVLIFILLFTCIILGALL
ECE2_HUMAN    MNVALQELGAGSNMVEYKRATLRDEDAPETPVEGGASPDAMEVGKGASPFSPGPSPGMTPGTPRSSGLFWRVTCPHLRSISGLCSRTMVGFQKGTRQLLGSRTQLELVLAGASLLLAALL
                                 * .* :...*:     . *::          ** *.    **:   :     .* :    *. *            .. :* : * .    *     : * ::*   :.::*.***

c30734_g1_i2  SRSKKEYKSLVLKE--DSVCLSEGCLDSAHYMLHAIDPKANPCEDFFQYSCGGWIKNHPIPDSQAFWGTFSSLWDDNMKEMKRLLTDPAIKNSSSSTIKLAKTFYDSCMNMDEINKQGAQ
ECE2_HUMAN    LGCLVALGVQYHRDPSHSTCLTEACIRVAGKILESLDRGVSPCEDFYQFSCGGWIRRNPLPDGRSRWNTFNSLWDQNQAILKHLLEN-TTFNSSSEAEQKTQRFYLSCLQVERIEELGAQ
                .         ::  .*.**:*.*:  *   :*.::*  ..*****:*:******:.:*:**.:: *.**.****:*     :*:** : :   ****.: : :: ** **::::.*:: ***

c30734_g1_i2  PMHNIIKDVGGWAVSENSTGTKWNKSSFNFNKQLKKLQLKYGESVFFTFNVEADAKNSSSNIIVIDQAGLGI-ERDYYLKN-VTDKNLVAYHKYMTTVGVLLGGQKDVVEKDMKDVLQFE
ECE2_HUMAN    PLRDLIEKIGGWNITG-----PWDQD--NFMEVLKAVAGTYRATPFFTVYISADSKSSNSNVIQVDQSGLFLPSRDYYLNRTANEKVLTAYLDYMEELGMLLGGRPTSTREQMQQVLELE
              *::::*:.:*** ::         *::.   ** : ** :   .*   : ***. :.**:*.*.**:* :**:** :  .*****:.  ..:* *.** .**   :*:****:    ..::*::**::*

c30734_g1_i2  MQIAKIFVPPNNRSQIDQMYRKMSIKDLGNICPEIPWLDFFQETFKGSATIQDSENVVVMATDYLKVLSPIIKKAGAKLLNDYMVWQIVRNSVGSLSRPFREAAHNLQKVLEGVSQPEE-
ECE2_HUMAN    IQLANITVPQDQRRDEEKIYHKMSISELQALAPSMDWLEFLSFLLS-PLELSDSEPVVVYGMDYLQQVSELINRTEPSILNNYLIWNLVQKTTSSLDRRFESAQEKLLETLYGTKKSCVP
              :*:*:* ** ::* :::*:****.:*  :.*.: **:*:.  :. .   :.*** *** . ***: ***  :* :*:::  ..:**:*::*::*:::..**.* *..* .:* :.* *..:.

c30734_g1_i2  LWKTCISDTDDSIGMALGNLFVKDKFEGSSRKDATKMINNIRAAFKARLPSLDWMDDKTRRAAVDKADAVINMIGYPEYIKDPKKLDEKYKGLTFSLTNYFANLHASTAFSMSKSKERLR
ECE2_HUMAN    RWQTCISNTDDALGFALGSLFVKATFDRQSKEIAEGMISEIRTAFEEALGQLVWMDEKTRQAAKEKADAIYDMIGFPDFILEPKELDDVYDGYEISEDSFFQNMLNLYNFSAKVMADQLR
               *:****:***::*:***.**** .*: .*:: *  **:*:***: *  **.:**:***:: ***:*:**:::* ***::*:: *:****:*::* :***:**: *.*   :*  .:* *:     **  .   ::**
```

```
c30734_g1_i2   KGVNKNKWLMTPQTVNAYYSPSRNQIVFPAGILQAPYYDKRYPKYCNYGSIGGAVGHELVHGFDNSGRMYDKNGNFGVQWWTNKSVEGFQVKADCLIKQYNQYSYYGKHEKGKFTLGENI
ECE2_HUMAN     KPPSRDQWSMTPQTVNAYYLPTKNEIVFPAGILQAPFYARNHPKALNFGGIGVVMGHELTHAFDDQGREYDKEGNL-RPWWQNESLAAFRNHTACMEEQYNQYQVNGERLNGRQTLGENI
               *   .:::* ********** *::*:***********:* :.:**   *:*.** .:****.*.**:.** ***:**:    ** *:*: .*: :: *: :*****.   *:: :*: ******
```

```
                                                                                                                                       PSI-BLAST
                                                                                                               aa     %I     E-value
c30734_g1_i2   ADNGGLKAAYLAYQHWVKQNGQEKTLPVLGKTNEQLFFIAYGQSWCASIRPEKARAMLEIDPHSPNKFRVLGTLSNLKEFAKAFNCPLGSNMNPSKKCVIW   781    100
ECE2_HUMAN     ADNGGLKAAYNAYKAWLRKHGEEQQLPAVGLTNHQLFFVGFAQVWCSVRTPESSHEGLVTDPHSPARFRVLGTLSNSRDFLRHFGCPVGSPMNPGQLCEVW   811    40.1      0.0
               ********** **: *::::*:*: **.:* **.****:.:.* **:    **.::  *  ***** :********* ::* : *.**:** ***.: * :*
```

## E) Peptidase M14 family

### E.1) Carboxypeptidase A2

```
CBPA2_HUMAN    -MAMRLILFFGALFGHIYCLETFVGDQVLEIVPSNEEQIKNLLQLEAQEHLQLDFWKSPTTPGETAHVRVPFVNVQAVKVFLESQGIAYSIMIEDVQVLLDKENEEMLFNRRRERSGNFNFGA
c31568_g1      MTALKLAVAVVMTVVAIGYGGKYSGDKILRIIPTSRQHIKMLTKLEEEQPMKLDFWKYPDNVGKPVDIHVQSQDFANFTSIMQSIGMKFKILAADLQVLMDQQND--IVHYADEHSN--WYVH
                 *::* : .   .  *    .: **::*.*:*:..::** * :** :: ::***** * . *:...::*   :.  .. :::* *: :.*:  *:***:*::*:  :.:   *:*.   :

CBPA2_HUMAN    YHTLEEISQEMDNLVAEHPGLVSKVNIGSSFENRPMNVLKFS--TGGDKPAIWLDAGIHAREWVTQATALWTANKIVSDYGKDPSITSILDALDIFLLPVTNPDGYVFSQTKNRMWRKTRSKV
c31568_g1      YHPLDEIKAKINNLVSQAGDRAELIPIGKSFEKRDQLAVKIKGAPQNNKPVLFINCGIHAREWVGPATCVYMLDQLVNEYNKDNTVKATLDKIDFIILPVLNPDGYVYSWTKDRMWRKNRKPT
               **.*:**. :::***::  . .. : **.***:*   .:*:.  . .:**.::::.********  **.::  :::*.:*.** ::.: ** :*:::*** ******:* **:*****.*. .

CBPA2_HUMAN    SGSLCVGVDPNRNWDAGFGG-PGASSNPCSDSYHGPSANSEVEVKSIVDFIKSHG-KVKAFITLHSYSQLLMFPYGYKCTKLDDFDELSEVAQKAAQSLR-SLHGTKYKVGPICSVIYQASGG
c31568_g1      SDPKCVGTDLNRNFAYHWGSDSGSSSLPCSEIYRGEGPMSEVEVRNVAEYLKALAPHVKGYLDIHSYSQLWMLPWGYKKEVTQDHLELDRVAKIGVQAIKNAGYNTDYKMGLSSNLLYRTSGS
               *.. ***.* ***:   :*. .*:** ***: *:* .. *****:.:.:::*: . :**.:: :****** *:*:***    :*. **..**: ..*::: : :.*.**:*  ..::*::**.
```

```
                                                                 PSI-BLAST
                                                      aa    %I    E-value
CBPA2_HUMAN    SIDWSY-DYGIKYSFAFELRDTGRYGFLLPARQILPTAEETWLGLKAIMEHVRDHPY   305   100    5e-97
c31568_g1      TKDYTYGALHIKYSYVLELRDKGDKGFLLPQSQILPTAMETFAGIKAMVASMDLTQE   269   38.2
               : *::*    ****:.:****.*  *****  ****** **: *:**::  :
```

### E.2) Carboxypeptidase A4

```
CBPA4_HUMAN    --------MRWILFIGALIGSSICGQEKFFGDQVLRINVRNGDEISKLS-QLVNSNNLKLNFWKSPSSFNRPVDVLVPSVSLQAFKSFLRSQGLEYAVTI
c27510_g1      MARGIGHLIIKTVVVLMISQSSVGRRKTYIGDRILRVTPKTEVDLKFLNGLVVDDKNYELDFWTFPTFVARSVDVHVSRSKCNQFERLLQLKNITFEVRL
                        :   :.:  :  **:  ::.::**::**:. :.  ::. *.  :*:.:* :*:**. *: . *.*** *.   . : *: :*: :.: : * :

CBPA4_HUMAN    EDLQALLDNEDDEMQHNEGQERSSNNFNYGAYHSLEAIYHEMDNIAADFP-DLARRVKIGHSFENRPMYVLKFSTGKGVRRPAVWLNAGIHSREWISQAT
c27510_g1      RNLQRLVNMEG-------MRLRSGRNFDS-SFHSYQRIVNEMRRLSRLHNNTIASVRSIGKTYEGRNMYLIRISANRTKNKPIIFLNCGIHSREWISIST
               .:** *:: *.         : **..**:  ::** : * :** .::  .   :*   .**:::*.* **::::*:.:  .:* ::**.********* :*

CBPA4_HUMAN    AIWTARKIVSDYQRDPAITSILEKMDIFLLPVANPDGYVYTQTQNRLWRKTRSRNPGSSCIGADPNRNWNASFAGKG-ASDNPCSEVYHGPHANSEVEVK
c27510_g1      CMYLARRLVNNYERDSQVRKLIEKLEWIILPVINVDGYLYTRNRDRMWRKNKSPNVNG-CMGTDLNRNFNYNWGGVGSAPNKPCSPIYPGSRAFSEVETQ
               .:: **::*.:*:**. : .::**:: ::*** * ***:**:.::*:***.:* *  ..  *:*:* ***:* .:.* * *.::*** :* *.:* ****.:

CBPA4_HUMAN    SVVDFIQKHGN-FKGFIDLHSYSQLLMYPYGYSVKKAPDAEELDKVARLAAKALASVSGTEYQVGPTCTTVYPASGSSIDWAYDN-GIKFAFTFELR--D
c27510_g1      NLAKFLFSRRKRIRGYVDFHSYGQLWMSPWGYKRQFPPNYRKQALAMNKVVKAIKHVHGTEFTYGPSSIMIYPTSGDATDWTFGVLGVTHSYGVELRPSL
               .:..*: .: : ::*::*:***.** * *:**. : .*: .:    . . ..**:  * ***:   **:.   :**:**.: **::.  *:..:: .***
```

```
                                                                                PSI-BLAST
                                                    aa     %I     %S     E-value
CBPA4_HUMAN    TGTYGFLLPANQIIPTAEETWLGLKTIMEHVRDNLY   308    100    100     8e-86
c27510_g1      MDLYGFVLPPSYITPVGKETFQGLKALAEVL-----   343    29.7   58.3
                . ***:**.. * *..:**: ***:: * :
```

**Figure S3) Metalloproteases.** The sequences used in the alignment in A) correspond to Angiotensin-converting enzyme (UniProt: Q6Q4G4, ACE_THETS) from *Theromyzon tessulatum*; in B) correspond to Nematocyst expressed protein 6 (Uniprot: K7Z9Q9, VMP_NEMVE) from *Nematostella vectensis*; in C) correspond to Zinc metalloproteinase-disintegrin-like EoVMP2 (Uniprot: Q2UXQ5, VM3E2_ECHOC) from *Echis ocellatus* and Zinc metalloproteinase-disintegrin-like BmMP (Uniprot: A8QL49, VM3_BUNMU) from *Bungarus multicinctus*; in D) correspond to Endothelin-converting enzyme 2 (Uniprot: P0DPD6, ECE2_HUMAN) from *Homo sapiens*; in E.1) correspond to Carboxypeptidase A2 (Uniprot: P48052, CBPA2_HUMAN) from *Homo sapiens* and in E.2) correspond to Carboxypeptidase A4 (Uniprot: Q9UI42, CBPA4_HUMAN) from *Homo sapiens*. In red, the residues that participate in the binding to the metal are highlighted.

**A) Cathepsin O-like cysteine peptidase protein. Peptidase C1 family**

```
c28869_g1        MMVMERYYGLHLAVLLLIYYSLVSIVIAEHEEEKEIRNLTQEEEIGYLFKQYVIRHNKTYLNDPKEYYKRREIFKQSLARQSKLNEREKELNGSAVYGVN
U6JMC8_TITSE     -MWLSHVY---IAFAVKICLFLFSLSVSNNEGD-----------ANELFDLYIKKFHKLYIPGTLEYDVRLSIFKESLRRIYSLN-KNRSHPDDAWWGLN
                  * :.: *   :*. : *   *.*: ::::* :            . **. *: :.:* *: .. **  * .***:** *  .** :::.  ..* :*:*

c28869_g1        KFSDWTPQEFKEFLR----LGYRKPSEIAINTDSDCLAVAPKLQLPVHQDWKKSGKVTAVKNQGKCGSCWAFTAVENIESQWAIKHDILKELSVQELISC
U6JMC8_TITSE     EYSDLTPEEFKQLMNKREIVRKNRNHKHVLRRSTEAINFKNITKLPKRVDWRQRGIVTPVKNQKECGACWAFSTVETLESMNALKTGKLVELSVQQVIDC
                 ::** **:***:::.    :  .:  : .:. .::.: .    :** : **:: * **.**** :**:****::**.:**  *:* . * *****::*.*

c28869_g1        GAGG--GCGGGNTCSALNWLKTHNYTVAPESEFPYQDKETTCIDKAKYSKEGVRIS-EFCCYENADEKSFMKPIIALIGPMAVNVDATLWHDYLAGIIQH
U6JMC8_TITSE     ATETNHGCDGGNTCNALHWMIENRVKILTDKEYPLTDKAEYC--KAIKPKEGVELKFNYTCEDLKESEGEMLKLLAFHGPLVASVDSSTWQDYLGGIIQY
                 .:    **.*****.**:*:  :. .: .:.*:*  **   *  **  .****.:. :: * :  :.:. *  ::*: **:...**:: *:***.****:

                                                                                  PSI-BLAST
                                                                      aa    %I    E-value
c28869_g1        HCTDTDINHAVQITGYDETGEVPFWIVRNSWGTDFGDKGYLYIKIGKNLCGLAGDPSYVIV-  326   100
U6JMC8_TITSE     HCED-NSNHAVQIVGYDLTGDIPYYIVRNSWGIEFGIDGYLKIAIGKNLCGIALEVSALDVV  313   40.1   8.9e-76
                 ** * : ******.*** **::*::******* :** .*** * *******:* : * : *
```

**B) Cathepsin B. Peptidase C1 family**

```
c28837_g1            MLSCVVLSLAIAFASA---MPANINPLSQEAIDYINSLEGMTWKAHMNFDDSIYTIDDIKAMCGALKSPEPLLKPRESLG-AMEVPDNFDSRVQWPDCAS
A0A1W7RAU0_9SCOR     MKIVLALFALLGFTSAKFVIPTSIDPLSQKMIDYINYMN-TTWKAGKNFEG--VSMKYIRGLMGVHPDSKKYRLPEYTIKVAANIPKTFDSRKQWPNCYT
                     *   :.*   :.*:**   :*:.*:****: ***** ::  ****  **:.   ::. *:.: *.   ..:    *. ::  * ::*..**** ***:* :

c28837_g1            IKEVRDQGSCGSCWAFGAVEAMTDRYCIHSENKVTPHISAEDLLSCCSSCGMGCNGGYPEAAWEYWKGTGIVTGGQYGTQEGCQPYLIAKCDHHVKGKLQ
A0A1W7RAU0_9SCOR     IREIRDQGSCGSCWAFGAVEAMSDRICIATNGKVSIEISAEDLLSCCTSCGNGCDGGFPSSAWEYWVDNGIVTGGLYNSQKGCQPYLIPSCDHHVKGHLP
                     *:*:******************:** ** ::.**: .*********:*** **:**:*.:***** ..****** *.:*:*******..*******:*

c28837_g1            PCKGIQPTPECKHKCEAGYNVTYPNDKRHGTSAYSVRSDPSEIQKEIMTNGPVEGAFTVYADFPTYRSGVYQHKTGGALGGHAIKILGWGVESGTPYWLV
A0A1W7RAU0_9SCOR     PCKGSVPTPNCVHLCEKGYNISFRNDKHYGKSSYQVSGDVQQIQSEIMKNGPVEGAFSVYGDFLTYKSGVYQHHTGEFLGGHAIRILGWGEENDVPYWLV
                     ****  ***:* * ** ***::: ***::*.*:*.* .* .:**.***.*******:**.** **:******:**  *****:***** *...*****

                                                                              PSI-BLAST
                                                                  aa     %I    E-value
c28837_g1            ANSWNPDWGDKGFFKILRGSDECGIESGVVGGMPKFS-----   317    100
A0A1W7RAU0_9SCOR     ANSWNSDWGDKGYFKILRGSDECGIEGEISAGLPDIDASGRL   323    59.5   3.2e-146
                     *****.******:************. : .*:*.:.
```

**Figure S4) Cysteine proteases.** The sequences used in the alignment in A) correspond to Cathepsin O-like cysteine peptidase protein (Uniprot: U6JMC8, U6JMC8_TITSE) from *Tityus serrulatus* and in B) correspond to Cathepsin B (Uniprot: A0A1W7RAU0, A0A1W7RAU0_9SCOR) from *Hadrurus spadix*.

**A) Retinoid-inducible serine carboxypeptidase. peptidase S10 family**

```
c25035_g1    MASVLH---ITLSFILAFSALCLTSTQAKRLKADIPDEKWSYVKVRDNAFMFWWLYGAQVDPSQRETKPLIMWLQGGPGGSSTGFGNFEELGPLDVNLKPRNTSWTSVANVLFVDNPVGT
RISC_HUMAN   MELALRRSPVPRWLLLLPLLLGLNAGAVIDWPTEEGKEVWDYVTVRKDAYMFWWLYYATNSCKNFSELPLVMWLQGGPGGSSTGFGNFEEIGPLDSDLKPRKTTWLQAASLLFVDNPVGT
             *   .*:    :.   ::*     * *.:   .     ::   .* *.**.**.:*:****** *   .  .: .   **:*******************:****  :****:*:*  ..*.:*********

c25035_g1    GFSYVTDKSAYTTNVSGIADDLLTMFKSFLDTMPVFQNVPFYIFCESYGGKMTSAFGVTLYKAILAKEIKCDFKGVALGDSWISPVDSVLSWGPYLYSVSLLDEDSLAKVNALANATAKA
RISC_HUMAN   GFSYVNGSGAYAKDLAMVASDMMVLLKTFFSCHKEFQTVPFYIFSESYGGKMAAGIGLELYKAIQRGTIKCNFAGVALGDSWISPVDSVLSWGPYLYSMSLLEDKGLAEVSKVAEQVLNA
             *****....**:.::: :*.*::.::*:*:.     **.******.*******::.:*: *****     ***:* ***********************:***::..**:*. :*: . :*

c25035_g1    VAMKQYKEATDLWSQTENMIAETTDNVDVYNILIHNAPPFPMMF-QSLGDKHLDSLYATHVGRLYTQSLSDLMNGPIRKKLGIIPPNVSWGGQSGDVFTYQIIDFMKPVIDDVGLLLDGG
RISC_HUMAN   VNKGLYREATELWGKAEMIIEQNTDGVNFYNILTKSTPTSTMESSLEFTQSHLVCLCQRHVRHLQRDALSQLMNGPIRKKLKIIPEDQSWGGQATNVFVNMEEDFMKPVISIVDELLEAG
             *    *:***:**.::* :* :.**.*:.**** :.:*. .*     .: :.** .*    ** :*   ::**:********** *** : *****: :**.     *******. *. **:.*

                                                                                                                                    PSI-BLAST
                                                                                                              aa    %I       E-value
c25035_g1    LQVVIYQGQLDMICDTPGAEEWIKKIKWSFLPDFLKSPRVPLYPPSGVKTRNTGAFLKKYSTLELYYILKAGHMVPSDAGEMALEMVKRVISE-   424   100
RISC_HUMAN   INVTVYNGQLDLIVDTMGQEAWVRKLKWPELPKFSQLKWKALY--SDPKSLETSAFVKSYKNLAFYWILKAGHMVPSDQGDMALKMMRLVTQQE   426   53.3     9e-156
             ::*.:*:****:* ** * * *::*:**. **.* :     .**  *. *: :*.**:*.*..* :*:*********** *:***:*:: * .:
```

**B) PC3-like endoprotease variant B. Peptidase S8 family. Furin subfamily**

```
c29821_g1    MDPG----VIFLFVWTCFLAVISSGFA------------------------------------------EVYTNTWVVYTDK-GKEYVDEIAEKKGFINHGQQGGLEGYYLLEHKNLG-RRTRRSLDRHTTDFLQETHITYAEQQ
NECB_HYDVU   MNYRGIYRRRYVFVLLLLVAVVNISYGWTVLKNKDYKRRYLSPSGVEKVRKHLSRKYVASRNNTQTFKKHYFSNTWAVHIDPPDNDVADRIAKKHGFTNIGKIGNIEGHYHFKHEEIG-ERELEKARHKTALLNLEDEVKFAEQQ
c31737_g2    -------MKLFHLWFPLAFILVSKVHSLEELEEDD---------------------------------DQELTNFWAVELDQDDESLAKEVAKKHGYSYGGRIGSLPNHFLLIREEKKSEGDNKRGDIPYPSLREHPNVKWHEQQ
                       : :     . ::.  .                                         .  :* *.*  *  .:. ...:*:*:*:   *: *.: .:: : :::   .   .      . :  . .:.: ***

c29821_g1    KVLRRQKR------EAF---------------NDPYYGDQWYIHNTGQTPGPKGLDINVLPVWKKNITGKNVVVTILDDGIEYTHPDLKDNYEASASWDFNSKDSDPLPRYSSDNINKHGTRCAGEVAAKSKNNVCGVGVAYNARI
NECB_HYDVU   KILERVKR------DGIP--------------NDPYFKDMWYLLNTGQASGPAGVDMNVVPVWKKNITGRGIVISVLDDGLDWTHPDLEANYDQTASIVLNDNDNDPMPRDS-DADNCHGTRCAGEAAAIANNGICGTGVAYNAKI
c31737_g2    RTLTLEKRGNFEQCDSLPGHLAEDEIKDMKYCNDPMFCKQWYIASYGQNSEPRGSDMGILEAWKMGYTGKGIKVTVMDDGLDHTHPDLKQNYDPKASRDLNDNDGDPKPRHEYDQY--HGTKCSGEIGAQANNSICGVGLAPHVSL
             : *   **      :.:               *** : . **: . ** . * * *:.:: .** . **:.: ::::***:: *****: **: .**  :*.:*.** ** . *    ***:*:** .* ::*.:**.*:* :. :

c29821_g1    GGVRMLDGDVTDIVEGGSLSLKTNFIDIYSSSWGPDDDGRTVDGPGTFAKKAFRDGIRKGRKGLGSIFVWATGNGGKYKDYCNCDGYITSIYTISIGAVDDHGKSPWYAEPCPATLAVTYSSGAANVKVPDLKIVTTDLNHKCTKD
NECB_HYDVU   GGVRMLDGQATDALEASALGFRGDHIDIYINCWGPKDDGKTFGKPGPMAAKALRLGAEQGRNRLGSIFVWATGNGGLTDDDCNCDGYTTSIFTISIGCIGDHGLSAYYTEKCSSTLAVTFN-GASHKEGRENKMVTTDLYHQCTEE
c31737_g2    GGVRMLDGKVTGITESAAFGLRPDYIDIFSASWGPKDNGKTLAGPSTLGKKALEHGAKHGRKGKGSIYVWASGNGGGSHDDCDCDGYASSIYTIAVGAISASGHSTFYDEKCASTMAVVYSGDSGRRFEGSHDLVTTGMDHSCIKH
             ********..*.  *..::.:: :.***:  .***.*:*:*.   *..:. **:. * .:**:  ***:***:****  .* *:**** :**:**::*.:.  * *.:* * *.:*:**.:. .:..    . .:***.: *.* :.

c29821_g1    HSGTSAAAPLAAGIFALVLEANPKLTWRDLQHLVVNTSKKNDPGDSEWIINGAGHHVNNKYGFGVLDTAALVDLATHPKWKTVNKQHVCRELGDIS----DKVIPRKGTFTSSLYSNGCMGKTNCITRLEHVRVYVTLSHARRGAL
NECB_HYDVU   FKGTSASAPLAAGIIALTLEANPLLTWRDVQALIVHTAQITSPVDEGWKRNGAGFHFNHKFGFGRLDANAMVNAAQS--WKNLPAQRKCTAASGFD----HQDIPRGDSLFINIPTVACESSSAQIAKVEHVVLTVSFVHRRRGDV
c31737_g2    FGGTSAAAPLAAGVFALVLQANKDLTWRDLQHIITKTSKVNDLQDVNWKTNGAGFKVNHKYGFGLLYAPALIKAAKT--WKTVPKQVKCEIQVTLPSDKRKRAIRKAGSVSFTVEADACKGTPNFVKKLEHVQAVINLRHRRRGVL
             . ****:******::**.*:**  *****:* ::.:*:: ..  *  *  ****.:.*:*:*** * : *::. *    **.:  *  *     :     .: * : .:.  .: : .* ...  : ::***   :.: * *** :

c29821_g1    SITLISPSGTRSELLKQRDEDYSSDGFQNWPFMTVFSWGENPRGIWTIKVTDHGNYQGQFKRWSLRLYGTCVETNYNITATEKQSCKEHCKVNCPNKPFAYVCKNCSQYCHCETGECVSLCDEDDEVDEKAKHCLPAKTLTIDEKE
NECB_HYDVU   SIDLISPKDTKSQMLSPRKYDDSDEGLDEWSFMTVYNWGENPKGIWRLKITDNPNQD-------------DVMNLFNGDNTDDVESLEERVIDTQTK-------------------------QNKAEWEKMR------------KE
c31737_g2    SIDIGSPSKTSSRLLAVRPLDQSTVGVKNWPFTTVHMWGENPAGEWKLTIRDNTKLHEKR----------LSKNDYFDDKQPEMQVRDEPEMTFESE-------------------------NMDSDDLPVS-------------N
             ** : **. * *.:*  *  * *  *..:*.* **. ***** * * :.: *: : .             . :      . .  :.  :   .:                         : .                      :

                                                                                                                                            aa    %I
c29821_g1    NCSKNCPRKCPSKKYCNCLTGRCMSMCGSGNKVDEKQRHCLAPAGSHPGDDTEDYSTGELSTFVKWLIIFSLLAILLATVLVMYLFKASGKFCWARPDMNKKSVQRNVSYAPVALNNPEGHNITIIEQEKRSLVL   686   39.4
NECB_HYDVU   NPYFDVP-------YPNGVRKNKVTIEGS----------------------TQDHVK----------------------------PKEGAKEPWGN--------YRNT-------NNINNNSSTAFKRKKKQ---   558   100
c31737_g2    DEAFEVPR-----PFRHEIELEDESEDEG----------------------RQEFAR----------------------------EEDEGKLNIFD--------VRSN-----IFGALDTWTLVLYGTAE-----   586   41.8
             :   : *      : : :  .  :   .                       ::.                             :  .*               *.        .  :  . .    :
```

| | PSI-BLAST E-value | E-value |
|---|---|---|
| c29821_g1 | | ------ |
| NECB_HYDVU | 0.0 | 4e-163 |
| c31737_g2 | ------ | |

**Figure S5) Serine Peptidases.** The sequences used in the alignment in A) and B) correspond to Retinoid-inducible serine carboxypeptidase (Uniprot: Q9HB40, RISC_HUMAN) from *Homo sapiens* and to PC3-like endoprotease variant B (Uniprot: P29145, NECB_HYDVU) from *Hydra vulgaris*, respectively.

## A) Beta-hexosaminidase subunit beta

```
HEXB_HUMAN   -MELCGLGLPRPPMLLALLLATLLAAMLALLTQVALVVQVAEAARAPSVSAKPGPALWPLPLSVKMTPNLLHLAPENFYISHSPNSTAGPSCTLLEEAFRRYHGYIFGFYKWHHEPAEFQ
c30952_g1    MAELCNTLL----VIFGLFCFALPSSTTKPSLSDPLNLQRYGGKQQILAEDYVKGSIWPKPQGQTPTGVKFSLLPKSFSFS-----IKGKTSDVLTDAVKRYMKLTF-------PDSGVT
               ***.  *    :::.*:  :* ::       . .* :*    . :    ..      ::** *  .  .  *     :  * *:.* :*         * :.  :* :*.:**     *           : .

HEXB_HUMAN   AKTQVQQLLVSITLQSECDAFPN-ISSDESYTLLVKEPVAVLKANRVWGALRGLETFSQLVYQDSYGTFTINESTIIDSPRFSHRGILIDTSRHYLPVKIILKTLDAMAFNKFNVLHWHI
c30952_g1    TKDDKLAEITSLEINVVNDYKPITLESDESYTLTINAPQSTLYAYEVWGALRGLETFSQVVYQDENDLYYAQENKIIDYPRFHYRGFMIDTSRHYLKPSIILKFLDAMSYGKFNVLHWHV
             :* :     :.*: ::    *   *   :.******* :: * :.* * .*************:****. . :   :*..*** *** :**::********   .**** ****::.********:

HEXB_HUMAN   VDDQSFPYQSITFPELSNKGSYS-LSHVYTPNDVRMVIEYARLRGIRVLPEFDTPGHTLSWGKGQKDLLTPCYSRQNK-LDSFGPINPTLNTTYSFLTTFFKEISEVFPDQFIHLGGDEV
c30952_g1    VDDQSFPFVSKTFPSLSGQGAYNNKTHIFSDDDVKNVIEYARMRGIRVIPEFDTPGHTFSWR-SIENLLTKCCDKQGKPTGGLGPIDPTIDSNYDFLKTFFGEVGSRYPDQYLHLGGDEV
             *******: * ***.**.:*:*.   :*::: :**: ******:*****:*********:**    . ::*** *  .:*.*    ..:***:**:::.*.**.*** *:..  :***::*******

HEXB_HUMAN   EFKCWESNPKIQDFMRQKGFGTDFKKLESFYIQKVLDIIATINKGSIVWQEVFDDKAKLAPGTIVEVWKDSAYPEELSRVTASGFPVILSAPWYLDLISYGQDWRKYYKVEPLDFGGTQK
c30952_g1    SFDCWKSNPNITAWMQKHGMGSNYSLLEQYYEQKLLNIVGGLKKQYIIWQEVVDNNVKVLPDTVVNVWKG-GWQAELAKVTKLGLKTILSSCWYLDGISYGLDWNKYYQCEPTNFTGSDA
             .*.**:***:*   :*:::*:*:::. **.:* **:*:*:. ::*   *:****.*::.*: *.*:*:***. .:    **::**   *: .***: **** **** **.***: ** :* *::

HEXB_HUMAN   QKQLFIGGEACLWGEYVDATNLTPRLWPRASAVGERLWSSKDVRDMDDAYDRLTRHRCRMVERGIAAQ-PLYAGYCNHENM----
c30952_g1    QKELVMGGTGCMWGEWVDGTNILPRTWPRALAIAERLWSSKATTDLGDATNRIWEQRCRYLRRGIPAENAVQSKYCRHEWPADEQ
             **:*.:** .*:***:**.**: ** **** *:.******* . *:.** :*: .:*** :.***.*: .: : **.**
```

| | aa | %I | PSI-BLAST E-value |
|---|---|---|---|
| HEXB_HUMAN | 435 | **100** | 1e-167 |
| c30952_g1 | 441 | 51.7 | |

## B) Beta-hexosaminidase

```
c30078_g1_i1   MELKPSNLSPAVLGLAFMLCLQSALVKTDMTQADVDNMASKLELRYKVISNS----------GQEYTAQIKLTNKGQLPIKKGEWTIYFCSIRLIVPQAGNQPVKFEHINGCLHKFQPTA
HEX_VIBVL      -------------------------MASDIDQKDVDYAAKNLKLTTSLVANKPKDCPPEAPWGACYRVEINLENTGSKSLNEN-VEIYFSSIHRTLGSKSEE-FKVEHINGDLHKITTTE
                                        : :*: * ***  *.:*:*  .:::*.           *  * .:*:* *.*. .:::.   ***.**:  : . .:: .*.***** ***: .*

c30078_g1_i1   NFPDLSPNNSVNIDVTAKHWVAARTDIMPNWYVSAPGLKPKAIASTVG-ESLDFVEPFL--------TKEQWKRFPGDTYDPYTAQKRYDEV-EIDVINSQDVPP-ILPQPLSMTGLDKG
HEX_VIBVL      KFKGLKGGKTKSFQVDFMNWIVSNSDFMPNYYVASEHLEGRNILNTVPIDAVHITEEVSGFTTGIKHTPNQLKRTANDLLPAATATTRYEQYSKVKDLGADAVSAHILPTPLETS-VHEG
               :* .*. .:: .::*   :*:.:.:*:***:**::  *: : * .**  :::.:.* .          * :* ** ..*    . ** .**::  ::. :.:: *.. *** **. : :.:*

c30078_g1_i1   KRMTIDREWKLYADNGLDKETKLLTDKFSATVVTSAPSESTKVLRIAIGNITITGSNSNSLEAYTLDIKVPTQTINIVGRSVRGVFYGIQSLL-AVTDDHNTVPEVSIQDAPRYSYRGMH
HEX_VIBVL      -SLNIAQGINIVSDALPADQVEALNFRFETLGVNTGTG--------VPVNVTIKADSSKKSGSYTLDVTS--SGIRIVGVDKAGAFYGVQSLAGLVTVGKDTINQVSINDEPRLDYRGMH
                 :.* :  :: :*    .:.: *. :*.:  *.:...         .  *:**....*:.  :****:.    . *.*** .   *.***:***    ** .::*: :***:* ** .*****

c30078_g1_i1   LDVGRNFMPKSAVLRLLDVMATYKMNKFHFHLTDDEGWRLEIPDLPELTQIGSKRCYDPTGLKCIQTDLGSGPDEPTSRT-YYTVNEYKEILQYANDRHIQVIPEFDMPGHGYAAVKSME
HEX_VIBVL      MDVSRNFHSKELVFRFLDQMAAYKMNKFHFHLADDEGWRLEINGLPELTQVGAHRCHDVEQNKCMMPQLGSGAELPNNGSGYYTREDYKEILAYASARNIQVIPSMDMPGHSLAAVKSME
               :**.*** .*. *:*:** **:**********:********* .******:*::**:*    **: .:****.: *.. : *** ::***** **. *:*****.:*****. *******

c30078_g1_i1   GRFKKLNST-NSLGAQKYLLTEAGDPSEYLSIQYFKDDAINPCLESTYAFIDHLVKEVVLMHRDK-QPLTIYHFGGDEVAHGAWTKSNACKNLAKQRGLNFSASDIVEQLKDYFVQRVAN
HEX_VIBVL      ARYRKFMAEGDVVKAEMYLLSDPNDTTQYYSIQHYQDNTINPCMESSFVFMDKVIDEINKLHKEGGQPLTDYHIGADETAG-AWGDSPECRKMFVAP---ESGVKNAKDINGYFINRISH
               .*::*: :  : : *: ***::..*.::* ***:::*::****:**::.*:*:::.*:  :*::  **** **:*.**.*  ** .*  *:::          *. . .::::.**::*:::

c30078_g1_i1   ITNAQVLNLAGWEDGLLGKGDIPYDRNFITNPEVYAYAWNNIWEWGGGKRAYELANAGYKVVMTQATHLYFDHPYEPDPEERGYYWATRFTDVRKTFGFMPDDLFANVENKINGEPLTRE
HEX_VIBVL      ILDAKGLTLGAWNDGLSHK---ALDASSLAGNPPKAWVWG-TMFWGGVDQYNSFANKGYDVVVTPPDAYYFDMPYENDPEERGYYWATRFNDTKKVFSFMPENVPANVEWMTDR------
               * :*: *.*..*:***  *    . * . ::.    *:.*.    *** .:  .:** **.**:* .   *** *** ************.*.:*.*.***::: ****    :

c30078_g1_i1   ELCKEENKCVDLKKKDNIIGMAGALWTETVRTADQMDSMIYPRLLALAERAWHKALWEDIGDETERDKK-----------------TTEDWEKFANLLGYKELKRLDRLGIKYRIPVPGA
HEX_VIBVL      --MGAKISATTGEKTHDFLGVQGALWSETIRTDAQVEYMVLPRMIAVAERGWHKASWEEEHKEGITYTSNVDGHEGTTHLNDNIATRDADWAHFSNILGYKEMPKLDKAGITYRLPVLGA
                   : ...  :*..:::*: ****:**:**  *:: *: **::*:***.**** **:   .*     ..                                ** :*:*:*****: :**: **.**:** **

c30078_g1_i1   KSSGDKILTKTAIPGLTVQYSDDNGQTWRDIPDKVHGKVKLATRSADGRRISRSVEYQGSTIKSSSSIPSAIQCLVFISVISYLFSKVDF
HEX_VIBVL      VIKNNILDVVTEFHGVAIQYSLD-GKTWHKYDDTKK-------PQVSTKALVRSVSTNGRTGRAVEVLAK--------------------
                 ..: : . * : *:::*** * *:**:.  *. :        ... : : ***. :* * :: . :..
```

| | aa | %I | PSI-BLAST E-value |
|---|---|---|---|
| c30078_g1_i1 | 862 | **100** | |
| HEX_VIBVL | 847 | 37.4 | 2e-178 |

**Figure S6) Glycoside Hydrolase Family 20.** The sequences used in the alignment in A) and B) correspond to Beta-hexosaminidase subunit beta (Uniprot: P07686, HEXB_HUMAN) from *Homo sapiens* and to Beta-hexosaminidase (Uniprot: Q04786, HEX_VIBVL) from *Vibrio vulnificus*, respectively.

## A) $PLA_2$

```
A2-AITX-Ucs2a   ---MKNN--IILVILLGI------SVFVDCLPLN-DQEEDKSLNAQESEVSAVQKRDILQFSGMIRCATGRSAWKYFNYGNWCGWGGSGTAVDGVDSCCRSHDWCYKRHDSCYPKIIPYIASTSGS----HPSCSITC--
c54261_g1_i1    MMMMKKKSTTTLIVLLGM------AFLVEGLSLN-NLEDDKRMNVKTGDGRAE-KRNLWQFGNMIKCATGRDAGDYNGYGNYCGWGGSGVPVDGVDRCCQAHDRCYDNHDSCNPKTNYYSYSKSGK----HPSCTISCGD
c18858_g1_i1    ---MFFK--TILLVIVAA------FLLTEIQGKRRNIKADESTKAVR---PAVMKRNLVQFGIMVYCMTGRFALDYNNYGNYCGVGGSGTPVDGLDRCCKVHDDCYGRYENCSPYYISYYVYRTGW----HPTCSLTCG-
c28522_g1       ---MKGAALLWMLVFAAASLQFVFSMEEEDESIQDLHKVESDSDLQEDEQSPVQKRNLIQFADMVRCATRRNPFDYNDYGNWCGVGGRGIPKDGVDRCCRAHDKCYERYHGCNPKWRTFRYRAKKRRRRFRSRCRITCK-
                   *       :::: .         .  :     .    : :.  .         .  **:: **. *: * * * . .* .***:** ** * . **:* **: ** ** .:..* *     :     .         :. * ::*
```

```
A2-AITX-Ucs2a   HSANNRCQRDVCNCDKVAAECFARNTYHPNNKH--------
c54261_g1_i1    STQNDQCERNVCSCDKVAAECFARNNYNNANKH--------
c18858_g1_i1    --DTDACKKAVCTCDKEAAECFARHKYNSKNNNSWFLWFRK
c28522_g1       -NKWGSCGRAVCECDRVCALCFARNRYHRKYKK--SWWR-
                  . * : ** **: .* ****: *:   ::
```

| | aa | %I | E-value | PSI-BLAST E-value | E-value |
|---|---|---|---|---|---|
| A2-AITX-Ucs2a | 111 | **100** | 1.1e-57 | 3.4e-39 | 1e-35 |
| c54261_g1_i1 | 117 | 59.0 | | ------ | ------ |
| c18858_g1_i1 | 139 | 40.7 | ------ | | ------ |
| c28522_g1 | 204 | 40.1 | ------ | ------ | |

## B) $PLA_2$ / Group I

```
c25131_g1_i1    -MTVKRLVKTVILLCFVLLAVAG-------------HTDSRRSKRSIAQLTTMIACATKN-NWVYYSNYGCYCGPGGKGKPVDEIDRCCQKHDMCYGRIMSSWKCMLS-LEVYFLSYEVTGCYNCKPS-N
c3686_g1_i1     MGALKLLVLLAVVACVACTSLDLGKLKKKSLSKALKTQVHTRARRSLYEFYKMITCETGR-SWQDYNLYGCFCGKGGTGTPVDALDQCCFDHDECYSQAAAS-VCPWP-LQIYLDSYWHKNCSECDASKN
PA2AD_NAJSP     -----MNPAHLLILAAVCVSPLG-------------ASSNRPMPLNLYQFKNMIQCTVPNRSWWDFADYGCYCGRGGSGTPVDDLDRCCQVHDNCYGEAEKISRCWPY-FKTYSYECSQG-TLTCKGG-N
A2-HRTX-Apt1a   -MQLYTY-FFTFSLVLILALADQENKSLDFTQEGGIA------KRGAFQFSYLIKKYTGR-NPLDYWGYGCWCGLGGKGTPVDGVDWCCYHHDMCFNSITQGPRPTCSKNAPYHKNYYFSGLKCSTGWLT
c513_g1_i1      -MGFLKLTCFLVVLNLSLSQAAFISAEADYEQDSGLSRGSYRSKRNLIQFARMITCATGR-DWSDYNEYGCWCGYGGSGRPVDGTDRCCQIHDRCYDSVVASNVCTIGQLQIYTEIYTRVNCDGCSYRRN
                           .                                 .  ::  :*    . . .    :  ***:** **.* ***   * **   ** *:.               *               .    .
```

```
c25131_g1_i1    SPCALAICKCDSSLSKCLKNKPLHK-RYKLYDQTKCSHNFLGS
c3686_g1_i1     SACEQALCECDSKAARCFKNNKWDP-QYDDYPQEKCA------
PA2AD_NAJSP     DACAAAVCDCDRLAAICFAGAPYNDNNYNIDLKARCQ------
A2-HRTX-Apt1a   SKCGRAICACDIAAVKCFMRNHFNNKYQNYKKN-IC-------
c513_g1_i1      NRCEQAICSCDSHAVRCFRSNRYNSRYANYNKQRLCNYN----
                . *   *:* **     *:      .     .    :   *
```

| | aa | %I | %I | E-value | PSI-BLAST E-value | E-value |
|---|---|---|---|---|---|---|
| c25131_g1_i1 | 124 | 37.0 | 33.1 | | ----- | ----- |
| c3686_g1_i1 | 118 | 41.9 | 34.7 | ----- | | ----- |
| PA2AD_NAJSP | 119 | **100** | 37.8 | 1e-23 | 3e-20 | 5e-19 |
| A2-HRTX-Apt1a | 119 | 37.8 | **100** | 2e-15 | 1e-19 | 2e-27 |
| c513_g1_i1 | 116 | 35.2 | 37.4 | ----- | ----- | |

## C) $PLA_2$ / Group III

```
c26312_g1       MDSYVSKIFVILAVILHASLCQAMYDWKTKTFIRKDNSKLVVPGTKWCGKGNNAMSFDDLGEHRETDLCCREHDHCPTYILPFQRRFGILNLYPSHLSLCSCEMKLYNCLWNVTSHVAVAV
PA23_HELSU      ----------------------------------------GAFIMPGTLWCGAGNAASDYSQLGTEKDTDMCCRDHDHCENWISALEYKHGMRNYYPSTISHCDCDNQFRSCLMKLKDGTADYV
PA2_XYLAI       MHALRSSVLALWLCLHVSVRAWMTYRSANGLDEYEPEDRIIFVGTKWCGNGNVAEGPEDLGSLKETDACCREHDMCPDLIEAGQSKHGLTNTASYTRLNCACDEKFYNCLKNSSETGSGAV
                                                        . ::. ** *** ** *  .  .:**  ::** ***:** *    *  . : :.*: *   .      * *: :: .** :  ..   :   *
```

```
c26312_g1       GRMYFNVLRVPCFHLVEKKVCKERSFDWWKFKYVCKKYGVEVKGQTFMPKRFHKQLQVQPSNWNATANGTMT
PA23_HELSU      GQTYFNVLKIPCFELEEGEGCVD-----WNFWLECTESKIMPVAKLVSAAPYQAQAETQSGEGR--------
PA2_XYLAI       RFTYFTLLGTMCYRNEHPLICVK------KGWFSCSKYELDQS----QPKRYQWFDVSSNFAFPRMLT----
                   **.:*   *:.  .   * .      :    *.:  :         .  ::     .
```

| | aa | %I | %S | PSI-BLAST E-value |
|---|---|---|---|---|
| c26312_g1 | 159 | **100** | **100** | |
| PA23_HELSU | 143 | 34.4 | 61.9 | 1e-30 |
| PA2_XYLAI | 140 | 33.3 | 54.1 | 2e-21 |

## D) $PLA_2$ / Group IX

```
c23289_g1       --------------------------MR--DPMLRVYLLLLMI--------HHTTAHQCR--VQVNGCSIPGNLPFLYKKKFTPACNKHDVCYSCGQYYHWSRKSCDVSFQNDMYYLCTL
X2G7F0_9CNID    --------------------------MQTYKGAILVFVTLGCF--------IGGVLSTCPADVHVNGCSIPLNAPFPYKELFKPVCNRHDVCYICGNRYGWTREICDVAFKRDMEQKCTD
c38288_g1       ---------------------MLPRMLLTRTISFTEFLIVLMFGFYSCSWCHTNSTRQCAADGYSNGCSVPLGMEAPYKASFTPACNKHDICYGCGHHYNWTRDLCDDAFLADMKNTCKN
A0A2B4RAB8_STYPI --------------------------MGNVSVLTIAWLLLLSMS-------WEVISAGSDCTIQTNGCSVPLGLKAPYQTILTPACVKHDVCYYCGELYRWNQEQCDKAFKRDMYKLCER
COMA_CONMA      -----------------------------------------------------QXPSTAELCKINSNACSVPFSX-IPCQKXFLAACDRHDTCYHCGKHFGFKQDDCDDAFFRDMTALC--
A7T072_NEMVE    MARVTSTKQYKNGMFSRYNGITPWNFEGIMSPVLVIFVIALPLSALASEGVTVDELDQDVCPFESNGCSVPGQLPMLYKRLFTPACHMHDVCYRCNKVHDWEKIDCDTKFWENMYFLCEG
                                                                      *.**:*         :  :  ..*  ** ** *.. . : :   **  *  :*   *
```

```
                                                                                                                                        PSI-BLAST
                                                                                                               aa    %I    %I     E-value   E-value
c23289_g1          PHN----KGFVFEILKGVKKVLKTKEEA-----------HCHTIAYLYYKAVDLLGGDRYDYISPEWCRDRCVEKIGSPNRRIGYSKNI  136   100   33.1               ------
X2G7F0_9CNID       RYNNR--KRFFSSIWNGITGATKVATEIAKWPFIRGDLNHCKHGAKIYYEAVKYFGKSYFVSSSPEWCKEECAKNAGNPN--I------  142  40.4   36.9   3.9e-31
c38288_g1          LHGNVRRKRFLSALLEVWYFIQGISKDK-----------RRCNLFAEMYYKGVRTFGKSHFEIRDHDYCWNTCADKHGSPFEDVPREFF-  147  33.1   100    -------      N.D
A0A2B4RAB8_STYPI   KGK----RWFIGDLLTKEKRCKSLGADA--------------------YYTAVRIAGHLHWEKNSPPWCQKACAKDLGDPNILIRH----  125  37.7   36.1   1.2e-26    6.1e-26
COMA_CONMA         --------------------AHGTDDE----------------------------GX--------------CPX---------------   77   N/D   N/D    1.4e-08    2.9e-09
A7T072_NEMVE       QYGTPDDHLDKRFLWNKQKMCNKVADG--------------------YHTAVQMFGESHWDTAKPDWCKKKCVKKIGDPRNHIQL----  138  36.1   31.2   2.2e-29    4.3e-18
                                                                   *                *
```

## E) PLA$_2$ / Group XIIA

```
PG12A_MOUSE   -MVTPRPAPARSPALLLLLLLATARGQEQDQTTDWRATLKTIRNGIHKIDTYLNAALDLLGGEDGLCQYKCSDGSKPVPRYGYKPSPPNGCGSPLFGVHLNIG-IPSLTKCCNQHDRCYETC
PG12A_HUMAN   MALLSRPALT----LLLLLMAAVVRCQEQAQTTDWRATLKTIRNGVHKIDTYLNAALDLLGGEDGLCQYKCSDGSKPFPRYGYKPSPPNGCGSPLFGVHLNIG-IPSLTKCCNQHDRCYETC
c25921_g1     -----------MNCYLLSLCFLATVLELVSSSDLSGGLGDLAK----------ILAELSGGNQ--CAFKCPRGFKPTPNPSHTPSS-NGCGS--MGLQIDTTNLPGFTECCNIHDKCYDTC
                         ** *    ..  :    .::*  . *  : :            :* **::  * :**. * ** *. .:.**. *****  :*::::   :*.:*:*** **:**:**

                                                                                                  PSI-BLAST
                                                                                 aa    %I    %S   E-value
PG12A_MOUSE   GKSKNDCDEEFQYCLSKICRDVQKTLGLSQN-VQACETTVELLFDSVIHLGCKPYLDSQRAACWCRYEEKTDL  167  31.8  64.1   4e-24
PG12A_HUMAN   GKSKNDCDEEFQYCLSKICRDVQKTLGLTQH-VQACETTVELLFDSVIHLGCKPYLDSQRAACRCHYEEKTDL  167  31.8  64.1   8e-24
c25921_g1     NGDRNQCDEDFKSCLDNVCLIEGLGRRLSEKELKSCETSSELFYSGTLALGCMAFKEAQRNACLCNGRTI---  146  100   100
              . .:*:***:*: **.::*        *::: :::***: **::...: *** .: ::** ** *. .
```

## F) PLA$_2$ / Group XV

```
c26388_g1     -------------MKREFCLFNLIFLIISAYFITICDAKPPVVIVPGDGGSRLQARLNKKTAPHIWCEKHSKGFFGIWLSVEELLPEVIDCWSDNMRLVYNTTSKQLTNPEGVEIRVPYFGTTEGIE
PAG15_HUMAN   MG--LHLRPYRVGLLPDGLLFLLLLMLLADPALPAGRHPPVVLVPGDLGNQLEAKLDKPTVVHYLCSKKTESYFTIWLNLELLLPVIIDCWIDNIRLVYNKTSRATQFPDGVDVRVPGFGKTFSLE
c28942_g1     MANFIFLVFFCAVFAWSTIENVSSSEISRGRERRSSSIKSPVVIVPGTGGSQLEAKLNKPSTLHWYCHRSSSSFFTLWLQKSSLLPWALDCWVDNMRLVYDKATNTVHNAPGVETRVPGFGQTNTIE
                             :  .         :  .     .. :.***:***  *.:*:*:*:* :. *   * : :..:* :**. . ***   :*** **:****:.::.    . **: *** ** *  :*

c26388_g1     YLDSSIDHPGEYFAAFVDALVKIGYVRGKTLRAAPYDFRHAPNMNKDYLNNLQKLIEETATQNNGDRVVLISHSLGCPYSKYFLDHIDQAWKDKYLHSWITIAGAWGGAAKLFRVISSGTYLGVPKY
PAG15_HUMAN   FLDPSKSSVGSYFHTMVESLVGWGYTRGEDVRGAPYDWRRAPNENGPYFLALREMIEEMYQLYGG-PVVLVAHSMGNMYTLYFLQRQPQAWKDKYIRAFVSLGAPWGGVAKTLRVLASGDNNRIP--
c28942_g1     YLDTN--NLIKYFKPMVDALVSWGYQRGTTVRAAPYDFRYAPDSANDYFTKLKSLVEETYKTNGNKQVTLLSHSLGCPYTLVFLNKQTNEWKNTYIKHWITLSGIWGGSAQLLRLYASGDSFGVP--
              :**..     .** .:*::**  ** **  :*.****:* **:    *:  *:.::**     ..  *.*::**:*  *:  **::  : **:.*:: ::::.. *** *: :*: :**    :*

c26388_g1     VLEPIKLRASTQTFGSSAFLLPSEKFWKANEPIVHTPTKNYSISNLDQFYKDLQFEVGQDIRN-LVAPVW--SDHPPNITLHCLYGTKVPTPEKFYFGPGEFPDTFPKTIMGDGDGTVNRQSLEGCM
PAG15_HUMAN   VIGPLKIREQQRSAVSTSWLLPYNYTWSPEKVFVQTPTINYTLRDYRKFFQDIGFEDGWLMRQDTEGLVE--ATMPPGVQLHCLYGTGVPTPDSFYYES--FPDRDPKICFGDGDGTVNLKSALQCQ
c28942_g1     IANPLTVRGEQRSCTSNNYMLPSDELWTADEVLLKTSTRSYTVKDYDAFFKNIGYPEGSFMRRNVQGLTSPLAQHSPNVTLYCLHGSGVQTPESYTYAAGDFPDGTPSVANGDGDGTVNARSLKACA
              :  *:.:* . ::  *. ::** :  *..:: :::*.* .*:: :   *:::: :  *  :*.    . .   :  .*.: *:**:*: * **:.: : .  ***  *.   ******** :*   *

                                                             PSI-BLAST
                                                  aa    %I   E-value  E-value
c26388_g1     QYKRTQFQNVTVQDFPAAEHMKIIGDKRLIDYVVKLLESIK  381  42.9            ------
PAG15_HUMAN   AWQSRQEHQVLLQELPGSEHIEMLANATTLAYLKRVLLGP-  379  100   1e-109   4e-114
c28942_g1     NFR--QMPKITIKDYSGVNHNGILSNEQAQSFVKSVLFQ--  380  42.3  ------
               ::  *  :: ::: .. :*  ::.:    ::  :*
```

**Figure S7) Phospholipases A2 (PLA2).** The sequences used in the alignment in A) correspond to A2-actitoxin-Ucs2a (Uniprot: A7LCJ2, PA2_URTCR) from *Urticina crassicornis*; in B) correspond to A2-hormotoxin-Apt1a (Uniprot: Q8WS88, PA2_ADAPA) from *Adamsia palliata* and Acidic phospholipase A2 D (Uniprot: Q9I900, PA2AD_NAJSP) from *Naja sputatrix*; in C) correspond to Phospholipase A2 isozymes PA3A/PA3B/PA5 (Uniprot: P16354, PA23_HELSU) from *Heloderma suspectum* and Phospholipase A(2) (Uniprot: I7GQA7, PA2_XYLAI) from *Xylocopa appendiculata circumvolans*; in D) correspond to Phospholipase A2 (Uniprot:X2G7F0, X2G7F0_9CNID) from *Rhopilema esculentum*, Conodipine-M alpha chain (Uniprot: A0A2B4RAB8, A0A2B4RAB8_STYPI) from *Stylophora pistillata*, Predicted protein (Uniprot: A7T072, A7T072_NEMVE) from *Nematostella vectensis* and Conodipine-M alpha chain (Uniprot: Q9TWL9, COMA_CONMA) from *Conus magus*; in E) correspond to Group XIIA secretory phospholipase A2 (Uniprot: Q9EPR2, PG12A_MOUSE) from *Mus musculus* and *Homo sapiens* (Uniprot: Q9BZM1, PG12A_HUMAN); in F) correspond to Lysosomal phospholipase A2 (Uniprot: Q8NCC3, PAG15_HUMAN) from *Homo sapiens*. Based on the similarity of the sequence, the residues that participate in the binding to calcium are highlighted in yellow and the residues involved in the catalysis are highlighted in magenta.

## A) BPI/LBP/Plunc superfamily. BPI/LBP family

```
BPI_HUMAN    MRENMARGPCNAPRWASLMVLVAIGTAVTAAVNPGVVVRISQKGLDYASQQGTAALQKELKRIKIPDYSDSFKIKHLGKGHYSFYSMDIREFQLPSSQISMVPNVGLKFSISNANIKISG
LBP_BOVIN    ----MVTSTGTLP--SLLLGTLLTFTSGALGANPGLVVRITDQGLEYVAQEELLALQSKLHKVTLPDFNGDVRIKHFGSVDYRFHSLNIQSCKLLGSALKLLPNQGLHFSISDSFIQVTG
c30974_g1    ----MKS--------CLIFVMLVLVIGVSLAKNPGIRIRLTNNGLAYAAKITKDILHEKIKILHIKDINGRADTP-IGHISYSLSSIHVNSFSIPTLTLKTKPGVGLELSVSGVNIDMGG
c24158_g1    ----MKN--------LLTLLVLVTLAVFSLATNPGIRLRLTDKGLSYVAKIAVEVLNEKLKTIKIPDIKGDEDTP-VGHISYSLSSIKVSSLSIHTYSLKTKADVGLELSVSSVSISMSG
c28966_g1    ----MAKMLLFFC-VAVAVFSGVARSSLPSPSEANVRLFLTKKGMDYANEAMMSSIVSEVRGLRIPPVS-ITKSIWWNRIDLSLYNSKVKNVNLGSSSLSFGYG-HIKLSLQLSSAEIVG
c25445_g1    ----MVRMWLIFCLVFIGVCFDGGRSSSPPPSKANVGLYLTKKGADYANVVMVNKTKSTMEGQKVPPVS-ISKSIWWNTIRLSLAKSKIRSVSLGDTSIKFQNG-FVKVLINIKSAEVAG
                 *              .         .    :.:  : ::.:*  *.           . :.    :    .             .      :  . .: . .:      :.    .   :.. :.      .: *

BPI_HUMAN    KWKAQKR---FLKMSGNFDLSIEGMSISADLKLGSNPTSGKPTITCSSCSSHINSVHVHISKSKVGWLIQLFHKKIESALRNKMNSQVCEKVTNSVSSELQPYFQTLPVMTKIDSV--AG
LBP_BOVIN    DWKVRKR---ILRLDGSFDVKVKGITISVNLLLDSEP-SGRPKVAVSSCSSHIRDVEVHIS-GDLGWLLNLFHNQIESRFRRVLESKICEIIEDSVTSELQPYLQTLPVTTEIDHL--AG
c30974_g1    DWHYRKDHWPHISDSGSFSLKGNGISFDALVKIGANP-QGHPTIAAAGCSSSVGGVDIHFS-GGASWLYNLFAGKVEHSIKKSLQSEMCKAANSAITDQGAKSFARFPIKKKLDKW--TF
c24158_g1    NWHYREDHWPHISDSGSFTLTASGISFDVSIKIGATA-DGHPTIAAAGCSASIGGVSIHFS-GGASWLYNLFASKIEGVIKSSLNSELCKAATSAINDQGAKAVAKFPVKKQLDKY--SL
c28966_g1    DYKYAGG--FLGRGRGTYEVKLKRVSLTKYLSLEANT-KGKPYVVNRECNTWIGDIDPTFR-GESSFILNLLVQLVKRSVQRDVQKMICKSITDGINERGKKLPKLYQDKFPIPQVPSAK
c25445_g1    EYNYNGG--ILGSGRGTYIVKLTNVWITKELSLGVNA-KRRPFLINGKCSNWIQDTSPEFT-GGSSFIINLLVALVRSSVNNEVRKKICTSINEAVDKQGKMLPTYYPENYNVSRVQCAQ
             .::             *.: :.   : :   : :    . . :* :     *.   : .     :   .   .:: :*:    :.   ..   :.. :*      ..: ..                :      :

BPI_HUMAN    INYGLVAPPATTAETLDVQMKGEFYSENHHNPPPFAP---PVMEFPAAHDRMVYLGLSDYFFNTAGLVYQEAGVLKMTLRDDMIPKESKFRLTTKFFGTFLPEVAKKFPNMKIQIHVSAS
LBP_BOVIN    LDYSLMGAPQATAQMLDVMFKGEIFSRDDRFPVAFLA---PVMNLPEEHSRMVYFAISDYAFNTASLVYHKAGFLNFTITDDVIPPDSSIRQNTKSFRAFVPRIARLYPNTNLELQGAVI
c30974_g1    IDYSLVSSPNSTVSFIDIFLKGEFESLAHPSEAPFSP---EPLPIDSESSSMVYGWITDYMFNTAGFAYMKAGFLNHTFVPDDFPKNSSYTPNTKTFKSLIPPLYDKYPNRPMTFKVYAT
c24158_g1    IDYALVKSPNSTFSFLDIFVKGEFESVAHPTEAPFSP---ASLPIDS-------------------------------------------------------------------------
c28966_g1    IHFPILESPSIQRGSVEFVIEGGIYSRTYTLDSSSRGQGISHIRYYPSHSKMLSIVLSDRMFNSLAELYFKAGVVQINEMQMDRVNPP------------------------MSIKVDLA
c25445_g1    INIPILQQPLIMTSYIEFLMEGGVNSSCYQLNNSSRLRGIRHIKYIPNLSKMISVVLSDRVINSMTDLYYKAGLMKLIEIQSTDLLDP------------------------LWAALHLT
             :.   ::   *       ::. .:* . *         .          :

BPI_HUMAN    TPPHLSVQPTGLTFYPAVDVQAFAVLPNSSLASLFLIGMHTTGSMEVSAESNRLVGELKLDRLLLELKHSNIG--PFPVELLQDIMNYIVPILVLPRVNEKLQKGFPLPTPARVQLYNVV
LBP_BOVIN    SAPCLNFSPGNLSTAAQMEIEAFVLLPNSVKEPVFRLSVATNVSAMLTFNTSKITGFLEPGKIQVELKESKVG--RFNVELLEALLNYYLLNNFYPKVNDKLAEGFPLPLLRKIQLYDPI
c30974_g1    KIPNVTSKTSGVSLLIECNIEMFVDLANGSSVFALSLGLTITADLHFNFKETNLTVNSTFVRLKASLVRSAIGDITKNIQGLQFYLDFFVNGMIIRKINDRGVKGFPLPVNDGLKLVNPR
c24158_g1    ------------------------------------------------------------------------------------------------------------------------
c28966_g1    RPPFTNTNTSGSFFTLYLNG------TIKDRSSIYEVGLHVTGEFKIQEKKRILSGSVSNLRFELTHCAENTP--KDLKEIIVPSLFSERVQVAKEVSESFMNDMAPLPLIPTVKIINER
c25445_g1    SSPQINTTVNSSLVVFKMNANFTPTCSVNEKSETFSVDLEIVGDFYLEEENWEVSGKVKNLRIEP-FFTDDIP--EKIKSLVLPVLKTHDSQIINQLNE-FLDKIIPLPKYSYVHFTNNQ
```

| | | aa | %I | %S | %I | %S | E-value | PSI-BLAST E-value | E-value | E-value |
|---|---|---|---|---|---|---|---|---|---|---|
| BPI_HUMAN | LQPHQNFLLFGADVVYK----------------------- | 456 | **100** | **100** | 43.0 | 79.5 | 2e-72 | 2e-39 | 2e-15 | 7e-04 |
| LBP_BOVIN | LQIHKDFLFLGTNVRYLRV--------------------- | 456 | 43.0 | 79.5 | **100** | **100** | 3e-73 | 1e-37 | 3e-14 | 2e-14 |
| c30974_g1 | IRSGKSFTLVSTDISYTPSEDPLEQEEKLIFFGNRKIPIV | 481 | 28.3 | 64.3 | 29.1 | 63.6 | | ----- | ----- | ----- |
| c24158_g1 | ---------------------------------------- | 248 | N/D | N/D | N/D | N/D | ----- | | ----- | ----- |
| c28966_g1 | VKYGQGYIVFECDLLEKE---------------------- | 434 | 20.4 | 58.6 | 19.1 | 54.1 | ----- | ----- | | ----- |
| c25445_g1 | FLRKEGYTVFESDIKV------------------------ | 436 | 19.3 | 53.7 | 16.9 | 53.2 | ----- | ----- | ----- | |

## B) L-amino-acid oxidase

```
OXLA_BUNMU   MNVFSIFSLVFLAAFGSCADDRRSPLEECFREADYEEFLEIARNGLKKTSNPKHVVVVGAGMAGLSAAY-VLEKAGHRVTLLEASDRVGGRANTYRDEKEGWYVNMGPMRLPERHRIVRTYIAKFGLKLNEFF
OXLA_CALRH   MNVFFMFSLLFLAALGSCADDR-NPLAECFQENDYEEFLEIARNGLKATSNPKHVVIVGAGMAGLSAAY-VLAGAGHQVTVLEASERPGGRVRTYRNEEAGWYANLGPMRLPEKHRIVREYIRKFDLRLNEFS
c29905_g1    ------------------------------------------------MAEVDADVIIIGAGISGLCGAKSLQDKHGLRVLVLEARDRVGGRTLTSKDPLYGYYDLGGTYIGATQTHIMN-LVKELGLDLYQTN
c10621_g1    ------------------------------------------------MVQSVVDVIVIGGGISGLCAAK-LLKEYGFDVLVLEARDRVGGRTETLEDPLFKYIDLGGSYVGADKHRILR-IAEELGVKTYPVE
                                                              ..   .*:::*.*::**..*   :    *   * :*** :* ***. *  .:     :    *.   . : :*:.    ::.:

OXLA_BUNMU   QENENAWYFIRNIRKRVWEVKKDPGVFKYPVKPSEEGKSASQLYRESLKKIIEELKRTNCSYILDKYDSYSTKEYLIKEGNLSRGAVDMIG--DLLNEDSSYYLSFIESLKNDDLFSYEKRFD-----EISGG
OXLA_CALRH   QENDNAWYFIKNIRKKVGEVKKDPGLLKYPVKPSEAGKSAGQLYEESLGKVVEELKRTNCSYILNKYDTYSTKEYLIKEGDLSPGAVDMIG--DLLNEDSGYYVSFIESLKHDDIFAYEKRFD-----EIVDG
c29905_g1    TEGCDIHLFLGIMKTISHDIPLGTS-FLGALDINNFWQAIEKMSKDVPLQAPWLATKAKEWDKMTFKQFIDEKCWTRYGRAVASAFVNVLLSSEPSDISLLFFLWFLRSGGGVIRN-WEDSLGGAQEYKIVGG
c10621_g1    CKGKTTDHLY--AKVRQHDGSFSTWNPLAIIDYWNVAYTLDKMCDTVPVDQPWEASRAIEWDHITAEEFFNKVCWTSYCKKRMVQNTHHALAVELWEISLLNYLWNRKVGEGVLKHSSADDVE--DEKQFIGG
              :.      :    :    :    ..      :.  :   :  ::        .      .::        :    :    .    :             ..     :   :  .   ::    .           . .       :: .*

OXLA_BUNMU   FDQLPKSMHQDIA-EMVHLNAQVTKIQHDAEKVRVAYQTPAKTLSYVTADYVIVCATSRAVRRISFEPPLPSKKAHALRSIHYKSATKIFLTCTQKFWEADGIHGGKSTTDLPSRFIYYPNHNFTSG--VGVI
OXLA_CALRH   MDKLPTAMYRDIQ-DKVHFNAQVIKIQQNDQKVTVVYETLSKETPSVTADYVIVCTTSRAVRLIKFNPPLLPKKAHALRSVHYRSGTKIFLTCTTKFWEDDGIHGGKSTTDLPSRFIYYPNHNFTNG--VGVI
c29905_g1    AQGICSKLAERIGSDNILFNSDVTHIDQTQSEYVTVR---CLDGSVYKAKCVISTAPPALLNRIRFTPFLPALKFQGSHRIPVGSIIRTVSVYENSFWREKDLTGYFLTDDGFGGPVTEAVEYSARDGNYPAL
c10621_g1    AQQISNKLKERLD-DKVILNSPVQQVKQ-ENDHVIVT---CKNRNSYQSKYVISAVPQALLSKISFEPPLPALKNQLIQRFPMGSSIKTFAFYETAFWRSKGLNGAILSD--FG-PIHTSMDDTKPGSNLPVI
              : : . : . :   : : :*: * ::.:   ..    .      .        :. **    ..    :   * * * * . * :   : .     *   : .          **. ..: *    :      .   :   .  .       .    .:

OXLA_BUNMU   VAYVLADDSDFFQALDIKTSADIVINDLSLIHQLPKNEIQALCYPSLIKKWSLDKYTMGALTSFTPY-QFQDYSETVAAPVGRIYFAGEYTARVHG-WLDSTIKSGLTAARDVNRASQKPSRIHLISDNQL-
```

```
OXLA_CALRH      IAYGIGDDANFFQALDFKDCADIVFNDLSLIHQLPKKDIQSFCYPSVIQKWSLDKYAMGGITTFTPY-QFQHFSDPLTASQGRIYFAGEYTAQAHG-WIDSTIKSGLRAARDVNLASENPSGIHLSNDNEL-
c29905_g1       LGFIIGDKARQFTTLPKDRRKTMVCEQFAKIYDS-QEALNVSQR--EDLHFLSDLKT------------------------------------------------------------------------
c10621_g1       MGIITGDQARYFCNKTQEERKQAICEQYANVFGC-KEALNPVHY--LDKDWLGDEYSGGCYVGIMPCGVMTKYGKALRCPIGRIHFAGTETATISAGYMDGAVQAGERAAREILSKFGKIAEQDVRDVKDVV
                :.   .*.:  *     .     : :: : :.   :: ::         .:  *  :

                                                                                           PSI-BLAST
                                                          aa    %I    %S    %I    %S   E-value  E-value
OXLA_BUNMU      ------------------------------------------ 499   100   100   75.2  90.8  9e-10    1e-24
OXLA_CALRH      ------------------------------------------ 498   75.2  90.8  100   100   5e-03    1e-13
c29905_g1       ------------------------------------------ 400   19.7  42.9  17.8  43.8           -----
c10621_g1       TPRRVTFGERCLPTVPVFVVGSAAVLALGGYLSFRYWVNHNV 509   20.0  48.7  19.5  477   -----
```

## C) Lysozyme c-1

```
LYSC1_ANOGA     MKVFSTVLLAIVACCAV-AEAKTFGKCELAKALANNGIAKASLPDWVCLVQNESAFSTSATNKNKNGSTDYGIFQINNKYWCDSGYGSND----------CKIACKNLLNDDITDDIKC
c21127_g1       MNLFPIKLVAVVLLIVEGSKAATKTKCQVVSALRAQGVPDGDLRDWLCLVKHESSFRYDVTGTNSN-SIDYGIFQLNDGYWCDRPSGTTSSTCWRLNTFGCDVSCSNLLNSDISDDANC
                *::*.   *:*:*    .   ::* *  **::..**  :*:....* **:***::**:*   ..*..*.* * ******:*: ****    *:..          *.::*.****.**:** :*

                                                       PSI-BLAST
                                               aa   %I   E-value
LYSC1_ANOGA     AKLIHKRHGFNAWYGWKNHCNGKKLPN--VSSCF     120  100   2e-32
c21127_g1       AVRIKNCKGFSAWYGWQNHCSDVSGAEYAYSSC-     130  43.2
                *  *:: :**.*****:***.. . .:   ***
```

**Figure S8) Antimicrobials.** The sequences used in the alignment in A) correspond to Bactericidal permeability-increasing protein (Uniprot: P17213, BPI_HUMAN) from *Homo sapiens* and Lipopolysaccharide-binding protein (Uniprot: Q2TBI0, LBP_BOVIN) from *Bos taurus*; in B) correspond to L-amino-acid oxidase from *Bungarus multicinctus* (Uniprot: A8QL51, OXLA_BUNMU) and from *Calloselasma rhodostoma* (Uniprot: P81382, OXLA_CALRH); in C) correspond to Lysozyme c-1 (Uniprot: Q17005, LYSC1_ANOGA) from *Anopheles gambiae*.

## A) Acetylcholinesterase

```
ACES_BUNFA      MPSCQPGKMPAPWPWWLQLLLCIPSCVAVLPGRAGELKVSTQTGSVRGLSLPVLDGHVS-AFLGIPFAEPPLGRMRFLRPEPVKPWQH---VLDATSYKPACYQMVDTSYPGFQGTEMWNPNRGMSE
c31683_g4       ---------------MFLSLFLSSCLIFLVNAVQYTPVVDTEGGKIRGFLGKLTTGHVVRKYLGIPFAKADR----FKPPTNATKWSD---VKNMTTFGKECPQ--APNAM---------SGKDISE
c30026_g1       MPYFN-------VVKYLVGLLVVIVSVTLGQEVNFTQVVTTEYGPVRGKITLLQNGLEASSYLAFPFAQATR----FERPTAPKRWTT---PINATEHGNMCPQFLIPGLP---------PTMKPDE
c32036_g1_i3    ---MMFLSSKCVVVFLSVHLFHDIVLAYPRNPRRVLTQQGYVSGIRQNIQINKAQSKTIYKFLGVPYVEKPIGDLRFKPPQALSNRNSSDYNYNATSLRSVCMQGENYFDSIRGAWPEFDPKRDMSE
                                 *:                    *  :.       .     :*..*:.:       *  *    .          : *      * *                   .    .*

ACES_BUNFA      DCLYLNIWVP--SPRPKDAPVLVWIYGGGFYSGAASLDVYDGRFLTYTQNVILVSLSYRVGAFGFLGLPGSPEAPGNMGLLDQRLALQWIQNNIHPFGGNPRAVTVFGESAGAASVGMHLLSTQSRT
c31683_g4       DCLNLNVFVP--DNAPSGLPVMVWVHGGAYIFGSN--RIYDGSFISSLGNVIVVAINYRLTVFGFLTTGENGDLKGNYGMLDQVQALRWVKRNIASFGGDPNKVTIFGESAGGYSVSLLMLSPLTKG
c30026_g1       DCLTISVYAPAYANETSNLPVLQWIHGGGYMFGFS--ALYDASVLATERNVIVVASQYRVGAFAWLSTG-TKDLQGNLGMFDMVNALQWTKRNVKSFGGNPDQVTLIGQSAGGIAVSTLMLSPLTEG
c32036_g1_i3    DCLYLNVYTPS-TTGTRKYPVIVHIHGGSYVVGTPTRDISPGEYLP-TRDVVLVTIQYRLGTFGFFTTG-DSEAPGNVGLLDQVEALKWVQQNIEGFGGDKNSVTLLGESAGGSSVVLHYLSPLSKG
                *** :.::.*     .    **:  ::**.:  *     :  .  :.   :*::*: .**: .*.::       :  ** *::*    **:* :.*:  ***:   **::*:***. :*    **. :.

ACES_BUNFA      LFQRAILQSGGPNAPWATVTPAESRGRAALLGKQLGCHFNNDSELVSCLRSKNPQELIDEEWSVLPYKSIFRFPFVPVIDGDFFPDTPEAMLSSGNFKETQVLLGVVKDEGSYFLIYGLPGFSK--D
c31683_g4       LFKNVIMQSGAATATWAAVSYEQMINITKAFGKRIDCTNLG--AMEECLKTKPTMEMIKHTTNDFS--------FAPVIDKHFLPDSPSVLLQKGQFEKYKVMVGVNNDEG-TLMTNAVPGMASGLN
c30026_g1       LYQNVIIMSGYANMILAVQKPEEALEVAREFGEHLGCTDMS--SLKECAKQKTMQEIIKAQSNVAPKE--FYARFSPVVDGEFLLDVPTNLIAEGKFRKANVMVCFTDDDG-GTLIQKAPGLFNGLN
c32036_g1_i3    LFHRGIAVSGVDLSPFAYKPKSAVLKASNSFVKKVNCTKQSSRETVRCLRSLPSTTFLQDFA---------YWKFQPFVDQHFLPDEPKTLRKNGKFHKLPLISGFVSQEGSFVVEHLAQYFGNTTH
                *::. *  **       *           :  : :::.*    .      * :       ::.                    * *.:* .*: * *  :  .*:*.:  ::  . .::*   :       :  .  .

ACES_BUNFA      NESLISR--ADFLEG----VRMSVPHANDIATDAVVLQYTDWQDQDNREKNREALDDIVGDHNVICPVVQFANDYAKRNSKVYAYLFDHRASN----LLWPPWMGVPHGYEIEFVFGLPLNDSLNYT
c31683_g4       VTNGIKR--VIFEGAVRSDQLKSVFNQKPPLPAAMIYRYTDWTNVSDPYRNRRMFMDMMADTMFVAPAIQSAKAFVNHTLPTYFYQLQYRADFGSFGR-VPSWVGAYHGADIPYVFGLPLSLNQLYT
c30026_g1       TDEGVTR--EAFLN--YTQRTFFVRDQNPEIIAAIVQEYTDWTDVDNINKTRRSVFDLTTDIGFRIPGLVASQAFAKKNMNTFVYQFDYKVPKMFDGNPRPEWMGAYHGADLFYLFGPSNWFTQLVS
c32036_g1_i3    TAKGFSKYLDYLLANEYKRKQQPKQDESGVAHNALEFQYTPWDDLNDQSLFREKLADMFSDYNFIAPTHASLTFHSDHGAPTYMFEFAHRSQY----HPKPKWMGVIHEDTTAYKFGLPLLNSKTKL
                 . ..:    :              . .     *:  .** * : .:     *. . *:  *  .  *        . .:   .: : : ::           * *:*. *     : ** .    .

                                                                                                                                   aa   %I    %S
ACES_BUNFA      PQEKE---LSRRMMRYWANFARTGNPTDPADKSGAWPTYTASQPQYVQLNTQPLATQPSLRAQICAFWNHFLPKLLNATVDPPRADRRRRSARA-------------------- 578  100   100
```

```
c31683_g4       KEGN----FSREIIKLWTNFAKTGNPNNPESIQTPWPQYDLNNQQYIALKPT-MAIETKLRAEHVAFWNKFIPDVLRSMPKPCEEPSDANKITTNIVVVIFAIIAAQLGTGYFD- 521 31.1  58.7
c30026_g1       TFGNESDQFGRLFREFIGNFAATGNPNPSNSNQ--WPKFTTDEQKVLLMHPN-PTVTENLVADKVAFWDEFIPKLQRKETPSVEKPKDQ-DYSLVAIIVMTVIIGVLLIILLIKW 516 28.7  56.8
c32036_g1_i3    KFDDNDKKVSDMVVTMFTNFAKFGNPTPRRVHGLKWDNFDSSKKAYLKINQN-PFMTSKYHPLRMAFWNSYFPTLLPEKPLLGAESVVSDTAGNRPKPN---------------- 556 30.1  59.3
                  .    ..  .     ***  ***.        *  :  .:   : ::        .  .   ***: ::* :
```

| | | PSI-BLAST | |
|---|---|---|---|
| | E-value | E-value | E-value |
| ACES_BUNFA | 4e-101 | 2e-82 | 2e-86 |
| **c31683_g4** | | ------ | ------ |
| **c30026_g1** | ------ | | ------ |
| **c32036_g1_i3** | ------ | ------ | |

## B) Venom phosphodiesterase

```
PDE2_CROAD      MIQQKVLFISLVAVTLGLGLGLGLKESVQPQA-----------------------------------------QSWSCSKLRCGEKRIANVLCSCSDDCLEKKDCCTDYKSICKGETSWL
PDE1_CROAD      MIQQKVLFISLVAVTLGLGLGLGLKESVQPQVSCRYRCNETFSKMASGCSCDDKCTERQACCSDYEDTCVLPTQSWSCSKLRCGEKRIANVLCSCSDDCLEKKDCCTDYKSICKGETSWL
c27095_g1       ------------------------------------------------------------------------------------------------------------------------

PDE2_CROAD      KDKCASSGATQCPAGFEQSPLILFSMDGFRAGYLENWDSLMPNINKLKTCGTHAKYMRAVYPTKTFVNHYTIATGLYPESHGIIDNNIYDVNLNLNFSLSSSTARNPAWWGGQPIWHTAT
PDE1_CROAD      KDKCASSGATQCPAGFEQSPLILFSMDGFRAGYLENWDSLMPNINKLKTCGTHAKYMRAVYPTKTFVNHYTIATGLYPESHGIIDNNIYDVNLNLNFSLSSSTARNPAWWGGQPIWHTAT
c27095_g1       -----------------------------------------------------------------------------------------------------------------------M

PDE2_CROAD      YQGLKAATYFWPGSEVKINGSYPTIFKNYNKSIPFEARVTEVLKWLDLPKAK------------RPDFLTLYIEEPDTTGHKYGPVSGEIIKALQMADRTLGMLMEGLKQRNLHNCVNLI
PDE1_CROAD      YQGLKAATYFWPGSEVKINGSYPTIFKNYNKSIPFEARVTEVLKWLDLPKAK------------RPDFLTLYIEEPDTTGHKYGPVSGEIIKALQMADRTLGMLMEGLKQRNLHNCVNLI
c27095_g1       KNGLVAASYFFVGSEVPIDGMLPNFTTYYNQSHPFEARVKKVLEWLDLPVQGSHVDPSTDDGKRRPDFITLYFHEPDSGGHLYGPDSSKVDNITRYVDKMIGLLFDGLEERSLKDKVNVI
                 :** **:**: **** *:*  *.: . **:* ******.:**:*****             ****:***:.***: ** *** *.:: :  : .*: :*:*::**::*.*:: **:*

PDE2_CROAD      LLADHGMEEISCD--RLEYMANYFNNVDFFMYEGPAPRIRSKNVPKDFYTFDSEGIVKNLTCRKPKQYFKAYLSKDLPKRLHYANNIRIDKVNLMVDQQWMAVRDKKFTRCKG--GTHGY
PDE1_CROAD      LLADHGMEEISCD--RLEYMANYFNNVDFFMYEGPAPRIRSKNVPKDFYTFDSEGIVKNLTCRKPKQYFKAYLSKDLPKRLHYANNIRIDKVNLMVDQQWMAVRDKKFTRCKG--GTHGY
c27095_g1       MLADHGMAATNCTGKKVTFLDEYGVTLNDIFSLQKWGGAFMSLSPKRGGSLNKTEILSRIQCKS--RYMRVFAKEELPKRLHYSYGSRIGDIIVMPEDGWLVGTNSSIP-CYGNIGYHGY
                :******   .*   :: :: :*  .:: ::          .  **   :::.  *:..: *:.  :*::.: .::*******: . **..: :* :: *:.  :..:. * *  * ***

PDE2_CROAD      DNEFKSMQAIFLAHGPGFNEKNEVTSFENIEVYNLMCDLLKLKPAPNNGTHGSLNHLLKNPFYTPSPAKEQS----SPLSCPFGPVPSPDVSG---------CKCSSITELEKVNQRLNL
PDE1_CROAD      DNEFKSMQAIFLAHGPGFNEKNEVTSFENIEVYNLMCDLLKLKPAPNNGTHGSLNHLLKNPFYTPSPAKEQS----SPLSCPFGPVPSPDVSG---------CKCSSITELEKVNQRLNL
c27095_g1       DSMAESMRGLFVAHGPSFKKGVVVDHFRNTEIYNMITSLLEIPPAPNDGMPGSLQHLLRSTVDAPKPIIQGDGKLFDPETCKYPGKKVAADRRDCRFCVCPYCDLKKDPEVEALDDLLDL
                *.  :**:.:*:****.*::   *  *.* *:**:: .**:: ****:*  ***:***:... :*.*  : .    .* :* :      .             *. .. .*:* ::: *:*

PDE2_CROAD      NNQAKTESEAHNLPYGRPQVLQNHSKYCLLHQAKYISAYSQDILMPLWSSYTIYRSTSTSVPPSASDCLRLDVRIPAAQSQTCSNYQPDLTITPGFLYPPNFNSSNFEQYDALITSNIVP
PDE1_CROAD      NNQAKTESEAHNLPYGRPQVLQNHSKYCLLHQAKYISAYSQDILMPLWSSYTIYRSTSTSVPPSASDCLRLDVRIPAAQSQTCSNYQPDLTITPGFLYPPNFNSSNFEQYDALITSNIVP
c27095_g1       SSEQVSQYQSHNLPWGLPQGGAGEGG-CILNQEEYINGFSTYLRVPLWVGYRLDGEKAGQRIPRQ-NCFRHDVRLTVNQTSTCTHYYRSGMDRGHLVPNADFDYNATAALNTFILSNIAP
                ..:  :: ::****:* **   ...  *:*:* :**..:*  : :*** .* :  ..: .  *   :*:* ***:.. *:.**::*  .      ::  .:*: .     :::* ***.*

PDE2_CROAD      MFKGFTRLWNYFHTTLIPKYARERNGLNVISGPIFDYNYDGHFDSYDTIKQHVN--NTKIPIPTHYFVVLTSCENQIN---TPLNCLGPLKVLSFILPHRPDNSESCADTSPENLWVEER
PDE1_CROAD      MFKGFTRLWNYFHTTLIPKYARERNGLNVISGPIFDYNYDGHFDSYDTIKQHVN--NTKIPIPTHYFVVLTSCENQIN---TPLNCLGPLKVLSFILPHRPDNSESCADTSPENLWVEER
c27095_g1       QYHIFNAGDWAILEDYVRDLAINFTTVYVISGSIFDENADGLRDEDKSVTRWIKNQTNTIAIPTHFYKIIVRCDETKTPYYKVPGCDGRLDVMSFILPHN--NKKHCWFQSFKEYLLDN-
                 :: *.     :    : . * : . : ****.*** * **  *. .::.: ::  ...*.****:: ::. *::  .   .  .* * *.*:******.  *.: *   * ::  :::
```

| | | aa | %I | %S | PSI-BLAST E-value |
|---|---|---|---|---|---|
| PDE2_CROAD | `IQIHTARVRDVELLTGLNFYSGLKQPLPETLQLKTFLPIFVNPVN` | 787 | 26.9 | 50.2 | 4e-105 |
| PDE1_CROAD | `IQIHTARVRDVELLTGLNFYSGLKQPLPETLQLKTFLPIFVNPVN` | 828 | 25.6 | 47.8 | 9e-105 |
| **c27095_g1** | `----SASVRDIEKLTGSTFFSGLPP--LEQARLKTFTPVKLWL--` | 630 | **100** | **100** | |
| | `    :* ***:* *** .*:***     *  :**** *: :` | | | | |

**C) Phospholipase B**

```
PLB_CROAD       MIRFGNPSSSDKRRQRCRSWYWGGLLLLWAVAETRADIHYATVYWLEAEKSFQIKD-VLDK-NGDAYGYYNDAIQSTGWGILEIKAGYGNQPISNEILMYAAGFLEGYLTASHMSDHFANLFPLMIKN--
T1DLW3_CROHD    MIRFGNPSSSDKRRQRCRSWYWGGLLLLWAVAETRADIHYATVYWLEAEKSFQIKD-VLDK-NGDAYGYYNDTIQSTGWGILEIKAGYGNQPISNEILMYAAGFLEGYLTASHMSDHFANLFPLMIKN--
PLB_DRYCN       MVRFGSAASSDNRRRRCWSWYWGGLLLLWAVAETRADLHYATVYWLEAEKSFQVKD-LLDK-NGDAYGYYNDTVQSTGWGILEIKAGYGSQLVSNEILMYAAGFLEGYLTASRMSDHVANLYHQMIKN--
c31154_g1       MALFAVG-------------YFFVVVCYCLGSRVQSASLNGTVYVNPIDGSYDVKFGVLDKMNGAAYGTFDDNVMSTGWGELNIVAGKGKK-YQDHEIMYAAGFLEGALTARQINDHYSNIYGIFFKNG-
V9KKP6_CALMI    ----MCVCVRGQGLGLGLGLPLLLVLAAVGVSASARGHLLRSVVLDEHSGRLRVVGGLNPH--SIAWANLTDRIRATGWSFLEIGT---NGKYNDSIQAYAAGVVEAAVTQPLIYMHWMNTMFNYCGPFK
c29069_g1       ---------------MASNALLFLAITASVCCFTIKATTYVSVVLLPDGKTLKVSTGKVFP--NVAWARFTDEIQVSGWSYLEVHS---NGSYPDMLQAKAAGMAEGYITSEMISMAYVNQYKGYCD---
                                         :     .         :*          :         . *:.    * : :**. *:: :   .    :     ***. *. :*   :     *

PLB_CROAD       --VIIEQKVKDFIQKQDEWTRQQIK-NNKDDPFWRNAGYVIAQLDGLYMGN-VEWAKRQKRTPLTDFEISFLNAIGDLLDLIPALHSELRKSDFRSMPDVSRIYQ-WDMGHCSALIKVLPGYENIYFAHS
T1DLW3_CROHD    --VIIEQKVKDFIQKQDEWTRQQIK-NNKDDPFWRNAGYVIAQLDGLYMGN-VEWAKQQKRTPLTYFEISFLNAIGDLLDLILALHSELRKSDFRSMPDVSRMYQ-WDMGHCSALIKVLPGYENIYFAHS
PLB_DRYCN       --VITEQKVKDFMQKQDEWTRQQIK-NNKDDPFWRNAGYIIAQLDGLYMGN-LEWAKRQKRTPLTKFEISFLNALGDLLDLIPALSPESRNNGFLSMSEISKMYE-WDMGHCSALIKVLPGYENIYFAHS
c31154_g1       --DPLVKKVAQFFIDQDKWMRNMITKNSNVSSSWRQMGNILAQFDGLISGY-KVHPATEK--PLDSFAFQVLNGVGDLIDLRTALSPETMP-DWDSMSEQQILSQIFTRGHCSALIKVLPAYENIFASHA
V9KKP6_CALMI    YKTKYCEKLRNYIETNLAWMERQMV-KAKEDEYWYQIRLTLLQLQGLEDSYNDRVSFPVGKFSINPFGFILFQMGGDLEDLETALND------------TNDGVVTGSGSCSALIKLLPGNKELLVAHD
c29069_g1       GESDFCDNLRKFVKENNDWMEEKIA-SSPSDDYWYQVKLILSQLNGLKIGYGLNTSFPA----ISDLEFFMFQASGDLEDLGNVLKK------------KKPHHVVGSGSCSALIKLLPNNKELFISHV
                      .:: .:.  :  * .. :  .   .  * :    : *::**  .     .       :  : : .::  *** **  .*                        * ******:**  :::  :*

PLB_CROAD       SWFTYAATLRIYKHLDFRITDPQTKTG-----RASFSSYPGLFGSLDDFYILGSGLIMLQTTNSVFNLSLLKKVVPE-SLFAWERVRIANMMADSGKTWAETFEKQNSGTYNNQYMILDTKKIKLQRS-L
T1DLW3_CROHD    SWFTYAATLRIYKHLDFKITDPQTKTG-----RASFSSYPGFLVSLDDFYILGSGLIMLQTTNSVFNLSLLKKIVPE-SLFAWERVRIANMMADSGKTWAETFEKQNSGTYNNQYMILDTKKIKLQRS-L
PLB_DRYCN       SWFTYAATLRIYKHLDFRIIDPQTKTG-----RASFSSYPGLLASLDDFYILGSGLIMLQTTNSVFNISLLQQVVPE-SLFAWERVRIANMMADSGKTWAQTFKKQNSGTYNNQYMILDTKKIKLRRS-I
c31154_g1       SWFSYSAMLRIYKHYNFNIKDTSTGAQ-----RMSFSSYPGFLESLDDFYIMDSGLILLQTTNNVFNKSLYEFVNPQ-SLLAWHRVRLSNLVARSGKEWAETFKLYNSGTYNNQYMILDLKLVELNKS-L
V9KKP6_CALMI    TWSNYQTMLRIIKKYNFSFSYSANGSVPVPGNVQSFSSYPGVIFSGDDFYILSSGLVTLETTIGNNNPDLWKFVQPVGSVMEWLRNIVANRLAKNGEQWASIFKRFNSGTYNNQWMIVDYNYFIPGRTDI
c29069_g1       TWNSYIGMLRVFKLYDFPYK-TKQGTEVIPGHRQSFSSSPGNLLSGDDFYLLSSGMVSQETTIGNSNKDLWKYVTPKNIVFEWMRVMVANSLARSSKEWTYMFSRFNSGTYNNQWMVLDYKLFEPGQP-I
                :* .*   **: *  :*    .   :        **** ** : * ****::.**::  :** .  * .* : : *   :: * *   ::* :* ..: *:  *. *******:*::*  : .   :. :

PLB_CROAD       EDGTLYIIEQVPKLVKYSDQTKVLR-NGYWPSYNIPFDKEIYNMSGYGEYVQRHGLEFSYEMAPRAKIFRRDQGKVTDMESMKFIMRYNNYKEDPYAKHN-------PCNTICCRQDLDRRTPVP-----
T1DLW3_CROHD    EDGTLYIIEQVPKLVKYSDQTKVLR-NGYWPSYNIPFDKEIYNMSGYGEYVQRHGLEFSYEMAPRAKIFRRDQGKVTDMESMKFIMRYNNYKEDPYAKRN-------PCNTICCRQDLDRRTPVP-----
PLB_DRYCN       EDGTLYIIEQVPNLVEYSDQTTILR-KGYWPSYNIPFHKVIYNMSGYREYVQKYGLDFSYEMAPRAKIFRRDQGKVIDIESMKRIMRYNNYKKDPYTKHN-------PCNTICCRQDLYYMTPVP-----
c31154_g1       HDGALWVVEQIPSYVASGDQTAILR-LGYWPSYNVPFYEKIYNLSGYPDFVRKHGTSYSYELAPRAKIFRRDQTTVNDMESMKHIMRYNDYKHDPYAFNN-------PMNAICSRGDLN-TNPIA-----
V9KKP6_CALMI    KEKLLTVLEQIPGQIVVRDKTELLMRQGYWPSYNIPYFEEIFNLSGNLPLVAQYGDWFTYDKNPRAKIFRRNQTMVTDMASMIKLMRYNDYLHDPLSKYPGCDPSSNGENSISSRSDLNPANGTYLFPAL
c29069_g1       KPGTLSILEQIPGITESADMSVHLEEQRYWASYNIPYFPLIYNQSGFVANRKAHGSWFDYYLSPRGQIFKRDQGKVVDLDSMMKLMRYNDYKNDPLSKCN-CTPPYSAENGISARCDLNPADGKYPFGAL
                .   * ::**:*      * : *     **.***:*:   *:* **       :*  : *   **.:**:*:*  * *: **  :****:* .** :            * *..* **

                                                                                             PSI-BLAST
                                                                        aa      %I     %I    E-value      E-value
PLB_CROAD       ----AGCYDSKVADISMAAKFTAYAINGPPVEKGLPVFSWVHFN-KTKHQGLPESYNFDFVTMKPVL-   518    52.3   33.2    1e-179       6e-85
T1DLW3_CROHD    ----AGCYDSKVADISMAAKFTAYAINGPPVEKGLPVFSWVHFN-KTKHQGLPESYNFDFVTMKPVL-   518    52.5   33.0    5e-177        N.D
PLB_DRYCN       ----AGCYDSKVADINMAAKFTAYAINGPPVEKGLPIFSWVHFN-ETTHQGLPESYNFDFVTMKPVL-   518    53.2   33.0    2e-176        N.D
c31154_g1       ----DGCYDAKVTDFFMANSLVSSAISGPTAQD-QPPFHWSQYPFPDTHNGMSDLFNFDFVKMKPKL-   522    100    34.2                 -----
V9KKP6_CALMI    EHRSHGGTDMKVTSFEMYKTFEMIAISGPAWDQVPPFQWSKSSYSGLIHMGHPDLWKFPPVMVRWS--   540    36.2   47.8    3e-61        9e-124
c29069_g1       GHREHGGTDCKITSLELFKKFQCRAVSGPTHDQQPVFQWSKTEWK--KPLGHPDKFDFEPITVSWDKN   525    34.2   100     -----
                     *  * *::.: :   .:   *:.**. :.                 * .: :.* : :
```

**D) Lysosomal acid lipase/cholesteryl ester hydrolase**

```
LICH_CROAD      ---------MWRLIIIAIL-FQGLVNSAMLERRKRGVD-------PETAMNISEIILFRGYPSEEYEVVTGDGYILCLNRIPYGKI-SQKTKEP-KPAVFLQHGLLADGSNWVTNLDYNSLGFALADAGFD
LICH_MACFA      -------MKMRFLGLVVCL-VLWTLHSEASGGKLTAVN-------PETNMNVSEIISYWGFPSEEYLVETEDGYILCLNRIPHGRK-NHSDKGP-KPVVFLQHGLLADSSNWVTNLANSSLGFILADAGFD
c22855_g1       ---------MFTLVILALLGFASARQVNFLVRQDQHVGH----ELPEVSMNVSQMIIYNGYPVENHEVTTDDGYILSLQHIPHGRQ-DKYKDVPSKKVVFLQHGLLASATNWVTNLPNQSFAFVLADQGFD
c28203_g1       MLCTNSASTATALLICMLLAFGPLQAVCRCINNTDLSKPGCIKVDPDVTRNVTQLIKSRGFPVESYEVTTEDGFILGVQRIPHGKKGVKTN--GSKPVVFLQHGLLADATNWVTEWEDDSFGYILADQGFD
c29969_g1       --------MSRALLVLVIFSITSHLVLGLPFLYKDKK---VEARHPDIDRNASQLIQNRGYPVEEHYVTTKDGYVLNLQRIPHGRNHQRNTLAGPKPVAFLQHGLLMDSTNWILNSANDSLGYILADEGFD
                            * :   : .                        *:   * :::*   *:* *.: * * **::* :::**:*:   :      * ..******* ..:**: :   .*:.: *** ***

LICH_CROAD      VWLGNSRGNTWSQKHINYTIKQKEFWMFSFNEMAMYDIPASVNFVLNKTGQEQLFYVGHSQGTTIGFIAFSVLPELAKKIKMFFGLAPVMTVKFSSGGLVKLGELP---EFLLKEIFGTKQIFPQNAVIKW
LICH_MACFA      VWMGNSRGNTWSRKHKTLSVSQDEFWAFSYDEMAKYDLPASINFILNKTGQEQVYYVGHSQGTTIGFIAFSQIPELAKRIKMFFALAPVVSVDFCTSPMAKLGRLP---DLLIKDLFGDKEFLPQSAFLKW
c22855_g1       VWLGNMRGNTYSRRHVRYPVDSDEFWDFSFDEMAKYDLPAMINYVTKVTGQPTLYYAGHSQGTMIGFIEFGRNPELIKKVKAFYALAPVTTVTYMGGALKYLSDLAPELEFLFK-IFGVREFLPSNFLIKW
c28203_g1       VWLGNIRGNTYSRKHVKYTPADRAFWDWSFEEMADYDIPAMINFALKTTGQEQLYYVGHSQGTLVGFLSFSEHPEVAKKVKLFFALAPIYHLNHVTSFVKDAAMTLGPVQELLL-PFGPTQFLPGFLLKQL
c29969_g1       VWLGNIRGNDYSQRHNTMDKNHPNFWDFSWQEMAEIDLPAMIDHVLKITGQQQVFYVGHSQGTLIGFTGFSSNQTLAKRIKTFFALAPVYTVAHCSDIVKGAAYALFPVEHGLQ-NHVSKEFDPSGLVKML
                **:** *** :*::*         ** :*::***  *:** ::.  : ***  ::*.****** :**  *.    : *::* *:.***:  : .  . :   .      :  :   .   :: *   .
```

```
LICH_CROAD     LATHVCG-QVLIDELCGNFFFLLCGFNEKNLNMSRVEIYSTHCPAGTSVQNMLHWSQAVKSGEVRAFDWGSRKENMAHYKQPTPPPYKMERMLVPTALWTGGHDWLSDRKDIAILLTLIPNLIYH--KEIP
LICH_MACFA     LGTHVCT-HVILKELCGNLCFLLCGFNERNLNMSRVDVYTTHSPAGTSVQNMLHWSQAVKFQKFQAFDWGSSAKNYFHYNQSYPPTYNVKDMLVPTAVWSGGHDWLADVYDINILLTQITNLVFH--ESIP
c22855_g1      FAKRVCQSSTILEMACSDFLFLIAGYDKKQLNETRVPVYVSHTPAGTSVKDVIHFAQLREAKKFQMYDYGSKEENQKHYNQDTPPEYNVSSLAVPTYLYWGGKDVLADPTDVKSLMAKLPPKIYLGNKYIE
c28203_g1      TKLGFCG-GKYSEKLCYDAAELIFGFDDKDQNMTRVPVLASHWPAGTSFKNIIHFGQVIYDGQTRRFNYGKWGNRRH-YHQEEPPAYNVSKLTTPTAMFLGQHDSLSVEADVAQLKEEIAEVVTLY-RVIP
c29969_g1      SDTGFCG-GMNSEAFCYKAGETLFGFDSKNLNMTRVPVIVAHWGSGTSFKNMVHFGQMVVSGKCQKYDYGFWNNYWHKYGQFSPPKYQVQKLTTPTVLYSGSNDNLASPLDVHNLKQHIQHVTVHA-EEIQ
                   .*      .  * .    : *::.:: * :** :  :*  :***.::::*:.*     : : :::*   :     * *  ** *::. : .** :: * :* *:    *:  *    :         . *

LICH_CROAD     EWEHLDFIWGLDAPQRMFRDMIQMMHKVQYAH
LICH_MACFA     EWEHLDFIWGLDAPWRLYNKIINLMKKYQ---
c22855_g1      DWEHLDFVWGMDAATEIYEDIIKRMKETDQ--
c28203_g1      EWNHIDFVLGMDAAKVLYNQIVRIIESGL---
c29969_g1      GWNHADFLFGMDAPQRLYSDIIRRMKADSRVL
                *:* **: *:**.  :: .::. :.
```

| | aa | %I | %I | E-value | PSI-BLAST E-value | E-value |
|---|---|---|---|---|---|---|
| LICH_CROAD | 383 | **100** | 65.5 | 1e-136 | 2e-118 | 1e-101 |
| LICH_MACFA | 376 | 65.5 | **100** | 2e-133 | 5e-115 | 3e-108 |
| c22855_g1 | 393 | 47.5 | 47.8 | | ------ | ------ |
| c28203_g1 | 390 | 43.4 | 43.8 | ------ | | ------ |
| c29969_g1 | 391 | 41.8 | 45.8 | ------ | ------ | |

**Figure S9) Other enzymes.** The sequences used in the alignment in A) correspond to Acetylcholinesterase (Uniprot: Q92035, ACES_BUNFA) from *Bungarus fasciatus*; in B) correspond to Venom phosphodiesterase 1 (Uniprot: J3SEZ3, PDE1_CROAD) and 2 (Uniprot: J3SBP3, PDE2_CROAD) from *Crotalus adamanteus*; in C) correspond to Phospholipase B (Uniprot: F8S101, PLB_CROAD) from *Crotalus adamanteus*, Phospholipase B-like (Uniprot: T1DLW3, T1DLW3_CROHD) from *Crotalus horridus*, Phospholipase-B 81 (Uniprot: F8J2D3, PLB_DRYCN) from *Drysdalia coronoides* and Phospholipase B-like (Uniprot: V9KKP6, V9KKP6_CALMI) from *Callorhinchus milii*; in D) correspond to Acid cholesteryl ester hydrolase from *Crotalus adamanteus* (Uniprot: J3SDX8, LICH_CROAD) and from *Macaca fascicularis* (Uniprot: Q4R4S5, LICH_MACFA).

## A) Lectins

### A.1) Techylectin-5B

```
c26308_g1      MRALGFTLILFGLQLLQSEGVQVAKIKKGGNVYVNVVVPHSKKMFG----LMKMLDKKFDRLMKAIGSE---------SGLSCPKMPKN-----CAEVLRCGGRTSKVYTVKPDSK-APF
TL5B_TACTR     MHTVLYSNMKNMFQLLSFLCVSLLVAGDVHHHAACSTVCSLKGILDSVSDLTDLAKERLATLQNPICSKDKAFYMETYTNVTQNKAEKNGLPINCATVYQQGNRTSGIYMIWPLFLNHPI
               *::: :: :   :***.   *.:    .  :  .  .*   * ::.    * .: .:::  * :.* *:          :.::  *  **     ** * : *.*** :* : *     *:

c26308_g1      KVYCDQKTNGGGWAVFQRRKDG---SVNFYRGWTDYKNGFGDLRGEFWLGLEKIYRLTHQTRNRLRVDLEDTSGVRAYAEYDYFAVSSERSKYSLSLGTYTGTAGDSLSYNRGTAFSTKD
TL5B_TACTR     SVFCDMETAGGGWTVIQRRGDFGQPIQNFYQTWESYKNGFGNLTKEFWLGNDIIFVLTNQDSVVLRVDLEDFEGGRRYAEAVEFLVRSEIELYKMSFKTYKGDAGDSLSQHNNMPFTTKD
               .*:** :* ****:*:*** *      ***: * .******:*  ***** : *: **:*    ******* .* * ***    * * ** . *.:*: **.* ****** :.. .*:***

c26308_g1      RDNDKWSSSCAVVYKGGWWFDRCHYANLNGQYLHGKHNKPWRGVVWRNWKGHSYSLKGDELKIRPS----------
TL5B_TACTR     RDNDKWEKNCAEAYKGGWWYNACHHSNLNGMYLRGPHEESAVGVNWYQWRGHNYSLKVSEMKIRPIIFVPGEGLPK
               ******...** .******:: **::**** **:* *::.  ** * :*:**.**** .*:****
```

| | aa | %I | PSI-BLAST E-value |
|---|---|---|---|
| c26308_g1 | 227 | **100** | |
| TL5B_TACTR | 289 | 41.9 | 3e-73 |

### A.2) Galactose-specific lectin nattectin

```
LECG_THANI     MASVPHFTVFLFLACALGIGANVTRRATS---------------SCPKGWTHHGSRCFTFHRGSMDWASAEAACIRKGGNLASIHNRREQNFITHLIHKLSGENRRT
c28373_g1      MRICGKQKVLSFLSRGTNVSIKFSSGYSVGFKGFRAVFSVFEDICPEGWLYFDSSCYRVFKSKSNWSKAREACWEMNGDLVSISNVDEQELISGMTS-WNGE-RNY
               *    : .*: **: . .:. :.:   :                **:** :..* *: ..:.. :*:.*. ** . .*:*.** *  **::*: :    .** *.

LECG_THANI     WIGGNDAVKEGMWFWSDGSKFNYKGWKKGQPDKHVPAEHCAETNFKGAFWNNALCKVKRSFLCAKNL-------------------------
c28373_g1      WIGLNDVEREGEYYWSDERPLDYQNWKDLEPNDDRGRENCVEIDSKNSKWNDDDCRKHYRFICETSRAPTVDASRWLMMGILCALTLSSLY
               *** **. :** ::***   ::*:.**. :*:..   *:*.* : *.: **:   *: :  *:* ..
```

| | aa | %I | PSI-BLAST E-value |
|---|---|---|---|
| LECG_THANI | 159 | **100** | 9e-21 |
| c28373_g1 | 195 | 25.9 | |

### A.3) Veficolin-1

```
FCNV1_VARKO    -----MTAWLDFPLALSPLVVVS----MKGGSFGQGSEANGSPQLTGLSECGADRIFLQGQAGIPGIPGVPGTNGLPGAKGDLGP---QGPPGERGSTG------IPGKAGPKGDKGDQG-----
c56356_g1      MSRITLFAFLIFLVGFFGNVQSQRRKVIRPTKGTRIRPTKGTPPTEASTSCGCCTVQLLGSSGTPGLPGRDGRDGKHGIKGDPGIRGQQGLPGKRGPPGTLPVGPIPFGASSAACTSDTAGHVRY
                    : *:* * :.:   *  .    ::  .  :   ::*:*   . :.**.  : * *.:* **:**  * :*  * *** *    ** **:**..*      **  *.. . ..* .
```

```
FCNV1_VARKO   -------EACS---------LASCQQQEAGAKDCKELLDRGETLT-GWYMIYPTTG---RGMRAYCDMETDGGGWLVFQRRLDGSVDFYRDWEAYKKGFGRQVSEFWLGNDKIHLLTSSGIQQLR
c56356_g1     ETSPNALELCNGVSWLPLVTESKGQSMQNPGRHCLDILNSGQSRGNGLYWIDPSGGSPEDSYQALCDMTTESGGWTLVATKVS--VDFVPISSRFSGRAAQTNND--DAASHIHQALKNVWKEIL
                     * *.         :. *. :  .:.* ::*: *::    * * * *: *    . :* *** *:.*** :.  ::.  ***     . :.   .:  .:   . .:**   ..  :::

                                          PSI-BLAST
                         aa    %I    %S    E-value
FCNV1_VARKO   IDVEDFNNSKTFAK   196   100   100    8e-10
c56356_g1     FRFSHRSDVYVIYN   238   24.2  47.2
              : ... .:  .: :
```

## A.4) Snaclec alboaggregin-B subunit beta

```
c6317_g1      -----------------MKRCSTSCGIGWTPFRSQCYKAINQKKSWNEASQHCKDK--GGNLTSIHSQEENNFLVSMGTSLSISDF-WIGIKTRPTCYRDVLYYSDGTAIKSHIASFKPF
SLBB_TRIAB    MGRFIFGSFGLLVLFLSLSGTGADCPSDWSSYDLYCYKVFQQRMNWEDAEQFCRQQHTGSHLLSFHSSEEVDFVVSKTLPILKADFVWIGLTDVWSACR--LQWSDGTELK---------
                               :.  .:.*  .*:.:   ***.::*: .*::*.*.*:::  *.:* *:**.** :*:**   .:  :** ***:.   :. *  * :**** :*

                                                           PSI-BLAST
                                            aa    %I    %S    E-value
c6317_g1      KYCPCDQQPATCKCVKSCKGENESRIWLFNNCNESLPFICKI--   142   100   100
SLBB_TRIAB    ----YNAWTAESECIASKTTDN---QWWTRSCSRTYPFVCKLEV   146   26.8  50.0   6e-13
                   :  .* .:*: * . :*    *  ..*..: **:**:
```

## B) Translationally-controlled tumor protein homolog (TCTP)

```
TCTP_ANOGA    MKIWKDVFTGDEMFSDTYKVKLVDDVMYEVYGKHVSR---TLGDVQLDGANPSAEEAEEGTESATESGVDIVLNHRLVET-GFSDKKQFTTYLKDYMKKLVTRLEE--KSPGEVEVFKTN
TCTP_PLUXY    MRIYKDIITGDEMFSDTYKMKLVDEVIYEVTGKLVTR---THGDVQIEGFNPSAEEADEGTDAATESGVDIVLNHRLMETYAFGDKKSYTLYLKDYMKKLVAKLEE--TAPDQVDVFKTN
TCTP_IXOSC    MLLFKDILTGDEMFTDSVKYKLVDDCIFEIECEHVTR---KVGEVALDGANPSAEEVEEGTEEGTESGLDLVLNMRLVET-GFS-KTDYKNYLKTYTKALMDKWKEDGKSEAEVNEAKSK
c7095_g1      MLLYQDIFTGKDILTDSYKMKLVDDFVYVVEANYGSKSGDQFGDEMFGGNPSAEEAGEDLEEAGSKSGFDVILNHEELEE--YNEQPSLKQFLK-LLKKIIAFAKEKIPKERFDGVFKPK
              * :::*::**.::::*: * ****: :: :  :  ::     *:  : *  .: *  ::  : .::**.*::** . :*   :. : . . :**   * ::   :*           *.:

                                                                    PSI-BLAST
                                                     aa    %I    %S    E-value
TCTP_ANOGA    INKVMKDLLG--RFKDLQFFTGE-SMDCEGLIAMLEYR-DIDGESVPVLLCFKHGLEEEKF-   171   34.4  65.6   4e-17
TCTP_PLUXY    MNKVMKDILG--RFKELQFFTGE-SMDCDGMVAMCEYR-DINGVSTPVMMFFKHGLLEEKF-   172   33.7  65.2   6e-16
TCTP_IXOSC    LTEAVKKVLP--RIGDMQFFLGE-SSNPDGIVALLEYRPNKSGGETPVVMFFKHGLLEEKQ-   173   30.6  58.9   2e-07
c7095_g1      MTEFFTSGPGKEKFSNFQIFFSSGYISEEGDVKAAPVLVDADETGLKAKIYYFKDLMEEVKM   179   100   100
              :.: ...     :: ::*:* ..   . :* :       : .     . : : :.* **
```

## C) CAP superfamily (cysteine-rich secretory proteins, antigen 5, and pathogenesis-related 1 proteins (CAP))

## C.1) Ectin

```
ECT_ACRMI     --MMQASFSICILSFY-LLSFCHGAPLPAFLRSVLS-----GNGMKEESRVLKRSAPVMQDEIPVCAQNQTDRYSSSSRLCRLVKDLGF------CDFDDLYQTVLQSCP-IGCGFCRVE
c22587_g1_i1  MNFLLDSFSKDALEAHNKYRDMHQVPSLKWSRSLAKQAEAWAQTLAKESCLRNGDTDDGENLYGVTGKSDLKGHEAVDKWYDEVRNYNFRRPGFQSDAGHFTQIVWKSVKEVGVGKAKTP
               ::  ***   *. :      * .*   : **: .     .: : :** : : .:   ::   * .:.: . :.: .:    *:: .*       .* ..: * * :*   :* * .:.

ECT_ACRMI     DGNWSVWGAWSP------------CSATCGDGQRSRSRSCTN--------PPPSGGGADCLGVSQEIEDCNRRSCEGIGGWSNWGQWSACSESCNIGIQARTRTCTNPP-----------
c22587_g1_i1  DGKVFVVARYRPPGNMANRFEQNVFPKASESSQSSSSRVYTTRIEKQEAEPVRSGRSERTRDRFNNDDDDFFKDFKDMGIGRKGGKTETVTHTKGPGTTVVTKTFTTDDGTGVRTVRKEV
              **:  * . : *               . :. ..* * **  *.         *  ** .     .  :: :*   :. :.:*   : *: .: :.: . *   . *:* *.

ECT_ACRMI     -PTIPEGACEGFS-----FETQICSTSGCNVSASVS-----------TAAATTSPVSSTAQTQIGPTVVSLTAKQQACLDAHNAKRAIHGSPPLEWDFTLAMNADEWANELAVTRQLEHD
c22587_g1_i1  HTSSHNGSDEDFGSRRRKWDDDDFFGKPRDLNANVRSRWDDKRDKSPSSDRARPPSSRTGGASPRSSSSSDKSFKDQCLSSHNKYRAMHSATALKWANDLARDAEKYAEKLARSGTLRHS
               .:  :*: *.*.      :: :     .  ::.*.*            ::  : .* * *. :.  .:  * .: :: **.:**  **:*.:..*:*   ** :*:::*::** :  *.*.
                                                                                                                                 PSI-BLAST
                                                                                                                 aa   %I    %S   E-value
ECT_ACRMI     PNIMNE--GENLFKSAGALECV--DAVERWFLEGKDYDYEDDNKLDDDTSNFTQLVWRNTTRVGVATVVEVVSEGSVETYIVARYTPPGNIEGKFEENVIKPSAEAL   379  100   100
c22587_g1_i1  SKSEREDTGENLAMFSGDYDSAGDRASDMWYEEVKDYNFSRKG-YQGGSGHFTQLVWKGSKELGMG---RAKTPDGRCTYVVARYRPAGNIVNYMDENVSPKRR---   428  21.8  48.9  6e-16
              .:  .*  ****   :*  :..   * : *: * ***::. ..  :..:.:******:.:..:*:.   .. : ..   **:**** *.*** . ::***
```

## C.2) Golgi-associated plant pathogenesis-related protein 1

```
GAPR1_HUMAN   MGKSASKQFHNEVLKAHNEYRQKHGVPPLKLCKNLNREAQQYSEALASTRILKHSPESSRGQCGENLAWASYDQTGKEVADRWYSEIKNYNFQQPGFTSG
c22343_g1_i1  --------------MLINLYRVMHGAPPLFWNEQLEKESQAWAEQLAQKGTLEHDTSMKDGENIAKLPASNSDVMN--AVDAWYDEEKMFDYKNPGFSKA
                             * **  **.***   ::*::*:* ::* **..  *:*... . *:   :*. :. *  .  ..* **.* * :::::***:..
```

```
                                                                                     PSI-BLAST
                                                                       aa    %I      E-value
GAPR1_HUMAN     TGHFTAMVWKNTKKMGVGKASASDGS-SFVVARYFPAGNVVNEGFFEENVLPPKK-  154   100     3e-26
c22343_g1_i1    TGHFTQLAWKGSKEMGLGIAPSKDKSEYYIVARFRPSGNVQGK--FVENVGPKVST  138   35.3
                ***** :.**.:*:**:* *.:.* *  ::***: *:*** .:  * *** *  .
```

### C.3) Venom allergen 3

```
c18947_g1_i2    MNGGGKNYVLSVSLSNATNTTDNATSNSCNMPTCAPPTSVPTSAASSASSSGPSNATSGPTTSSSHASSAPTSGASASTATGAPSSGGSSGSNDCQQKALAEHNAKRSVHNSPTMTIDPQ
VA3_SOLRI       -------------------------TNYCNLQSCKRNNAIHTMCQYTSPTPGPMCLEYS-NVGFTDAEKDAIVNKHNELRQRVASGKEMRGTN----------GPQPPAVKMPNLTWDPE
                                         :* **: :*   .:: * .  ::.:.**     . ... :.*.. .  .   .    ..*.    *:*          ..: .. : *.:* **:

c18947_g1_i2    LNAAAQAYAEKLATMKDIIHSPMNERQGQGENLAQRCSLPDTGFDCKSATDMWYNEVKKYD--WGSPGYTDG-----TGHFTQLVWKGTTKLGIGKAPFVNEKNLNCYVIVGRYKMAGNV
VA3_SOLRI       LATIAQRWANQCTFEHDACRN--VERFAVGQNIAATSSSGKNKSTLSDMILLWYNEVKDFDNRWISSFPSDGNILMHVGHYTQIVWAKTKKIGCGRIMFK-EDNWNKHYLVCNYGPAGNV
                * : ** :*:: :  :*  :.   ** . *:*:*  .*  ..    ..   :******.:*  * *.  :**     .**:**:**  *.*:* *:  *  *.* * : :* .*  ****

                                               PSI-BLAST
                                 aa    %I    %S    E-value
c18947_g1_i2    KGQFPDNVQKGSYVARRSLE    211   100   100
VA3_SOLRI       LGAQIYEIKK----------    253   25.2  44.4   7e-23
                 *    :::*
```

### D) Acrohargin I

```
                                                                                                                          PSI-BLAST
                                                                                                           aa    %I       E-value
c25993_g1_i1      MNQILNIFLVLGMIIYSVESRN--------KACLDACFGDYSSCQMSTSCYDQASCAKCNALYSTCQQS-CGDKRGYVPEAVIDL[RR]RLRLGQ   64    100
comp58542_c0      MNQILNIFLVLGMIIYSVESKD--------KACLDACFATYMRCQLSNACYDKPSCRQCNTTYRTCVNS-CAKKRSYVPEAVIDL[RR]QLRLGD   64    64.1       nd
comp66995_c0      MNQILNIFLVLGMIIYSVESKD--------KACLDACFATYMRCQLSNACYDKPSCRQCNTTYRTCVNS-CAKKRSYVPEAVIDL[RR]QLRLGD   64    64.2       nd
U-AITX-Aeq5a      MNQVMTIFLVLGVIVYSVESSSTPDG--TWVKCRHDCFTKYKSCQMSDSCHDEQSCHQCHVKHTDCVNTGCP----------------------   50    23.9       8e-15
U-AITX-Aeq5b      MNQVMTIFLVLGVIVYSVESSLTPSSDIPWEKCRHDCFAKYMSCQMSDSCHNKPSCRQCQVTYAICVSTGCP----------------------   50    23.3       9e-14
                  ***::.******:*:*****            * . **  *  **:* :*::: ** :*:. :  * .: *
```

### E) Three-finger toxin-like

```
                                                                                                                                                   PSI-BLAST
                                                                                                                                  aa    %I         E-value
c27526_g1       MKLTLFFLVVLAVA-IPIAFSLKCRICA--GKICTGPTTCPTG-DTCFTT-TYKNTSSVVKGCVTASACNQTMSMCQKYP-NYCVAECCTTDGCN---SSGGFVQINVAMVTLMAFFASIFYLFSH   70    100
XP_020916494    MKLALLCLAVFAVV-IPSVFSIKCYNCV--LSVCKETSCLGVG-DRCIHT-SYKNSSKVVKSCATESLCNTTKSACDKVK-NYCTTSCCMTDGCNKGDSGVAALQFNYTMLAFMALLSALLSFVKF   70    48.6       3e-23
3NO23_NAJAT     MKTLLLTLVVVTIVCLDLGYTLTCLICP--EKYCNKVHTCLNGEKICFKK-YDQRKLLGKG---YIRGCADT---CPKLQ-NRDVIFCCSTDKCNL--------------------------------   65    34.3       0.024
3NO21_NAJAT     MKTLLLSLVVVTIVCLDLGYTLTCLICP--EKYCNKVHTCLNGEKICFKK-YDQRKLLGKR---YIRGCADT---CPVRK-PREIVECCSTDKCNH--------------------------------   65    32.4       0.014
3LK6_BUNMU      MKTLLLSLVVVTIVCLDLGYTRTCHIST--S---STPQTCPKGQDICFRK-TQCDKFCSIRGAVIEQGCVAT---CPEFRSNYRSLLCCRTDNCNP--------------------------------   66    33.3       0.012
JN112568        --MQLFGPLIFLVT-LSAAFGLRCHSCS--GKSCTGPMTCPQGTDRCAIA-ESNG--AVAKSCMLSALCVSP--------------IKCCETDLCNSATPTGSSVLLLLVSSGIITLFL--------   62    34.2       5e-05
LYNX1_MOUSE     -MTHLLTVFLVALMGLPVAQALECHVCAYNGDNCFKPMRCPAMATYCMTTRTYFTPYRMKVRKSCVPSCFET--VYDGYSKHASATSCCQYYLCN-GAGFATPVTLALVPALLATFWSLL------   72    33.8       0.022
                    *:   :. :  :       *  .                   *                     *  .               **    **
```

**Figure S10) Other Proteins.** The sequences used in the alignment in A.1) correspond to Techylectin-5B (Uniprot: Q9U8W7, TL5B_TACTR) from *Tachypleus tridentatus*, in A.2) to Galactose-specific lectin nattectin (Uniprot: Q66S03, LECG_THANI) from *Thalassophryne nattereri*, in A.3) to Veficolin-1 (Uniprot: E2IYB3, FCNV1_VARKO) from *Varanus komodoensis* and A.4) to Snaclec alboaggregin-B subunit beta (Uniprot: P81116, SLBB_TRIAB) from *Trimeresurus albolabris*; in B) correspond to Translationally-controlled tumor protein homolog from *Anopheles gambiae* (Uniprot: Q7QCK2, TCTP_ANOGA), *Plutella xylostella* (Uniprot: Q60FS1, TCTP_PLUXY) and *Ixodes scapularis* (Uniprot: Q4PLZ3, TCTP_IXOSC); in C.1) correspond to Ectin (Uniprot: B3EWZ8, ECT_ACRMI) from *Acropora millepora*, C.2) to Golgi-associated plant pathogenesis-related protein 1 (Uniprot: Q9H4G4, GAPR1_HUMAN) from *Homo sapiens* and C.3) to Venom allergen 3 (Uniprot: P35779, VA3_SOLRI) from *Solenopsis richteri*; in D) correspond to U-AITX-Aeq5a (Uniprot: Q3C258, ACR1_ACTEQ) and U-actitoxin-Aeq5b (Uniprot: Q3C257, ACR1A_ACTEQ) from *Actinia equina*, and comp58542_c0 and comp58542_c0 from *Anthopleura elegantissima*; in E) correspond to uncharacterized protein (RefSeq:XP_020916494) from *Exaiptasia pallida*, Probable weak neurotoxin NNAM3 (Uniprot: Q9YGI1, 3NO23_NAJAT) and Probable weak neurotoxin NNAM1 (Uniprot: Q9YGI2, 3NO21_NAJAT) from *Naja atra*, Kappa-6-bungarotoxin (Uniprot: Q9W729, 3LK6_BUNMU) from *Bungarus multicinctus*, Ly-6/neurotoxin-like protein (JN112568) from *Epinephelus coioides* and Ly-6/neurotoxin-like protein 1 (Uniprot: P0DP60, LYNX1_MOUSE) from *Mus musculus*.
